# An Automated, Open-Source Workflow for the Generation of (3D) Fragment Libraries

**DOI:** 10.1021/acsmedchemlett.2c00503

**Published:** 2023-05-02

**Authors:** Tom Dekker, Mathilde A. C. H. Janssen, Christina Sutherland, Rene W. M. Aben, Hans W. Scheeren, Daniel Blanco-Ania, Floris P. J. T. Rutjes, Maikel Wijtmans, Iwan J. P. de Esch

**Affiliations:** † Amsterdam Institute of Molecular and Life Sciences (AIMMS), Vrije Universiteit Amsterdam, De Boelelaan 1108, 1081 HZ Amsterdam, The Netherlands; ‡ Institute for Molecules and Materials, Radboud University, Heyendaalseweg 135, 6525 AJ Nijmegen, The Netherlands

**Keywords:** FBDD, library design, cheminformatics, KNIME, 3D fragments, cyclopropane

## Abstract

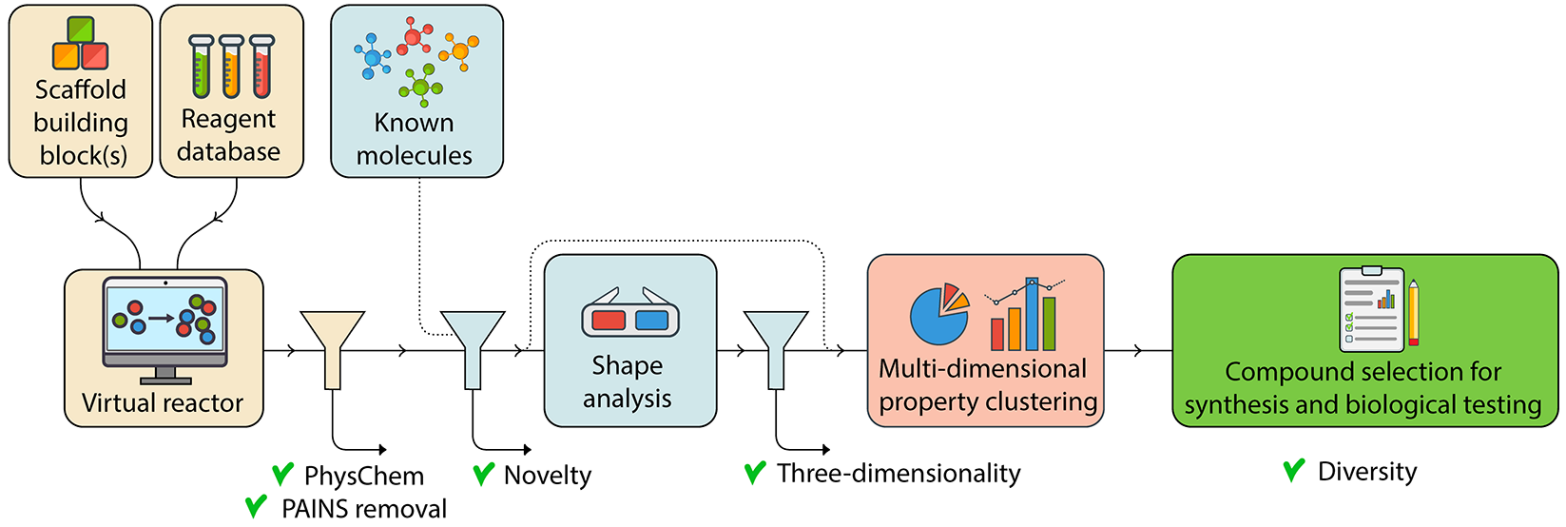

The recent success of fragment-based drug discovery (FBDD) is inextricably linked to adequate library design. To guide the design of our fragment libraries, we have constructed an automated workflow in the open-source KNIME software. The workflow considers chemical diversity and novelty of the fragments, and can also take into account the three-dimensional (3D) character. This design tool can be used to create large and diverse libraries but also to select a small number of representative compounds as a focused set of unique screening compounds to enrich existing fragment libraries. To illustrate the procedures, the design and synthesis of a 10-membered focused library is reported based on the cyclopropane scaffold, which is underrepresented in our existing fragment screening library. Analysis of the focused compound set indicates significant shape diversity and a favorable overall physicochemical profile. By virtue of its modular setup, the workflow can be readily adjusted to design libraries that focus on properties other than 3D shape.

Fragment-based drug discovery (FBDD) is proving itself as a productive drug discovery approach that is leading to a growing list of clinical candidates and six approved drugs as of November 2022.^1,2^ Compared to high-throughput screening (HTS), FBDD utilizes smaller molecules—termed fragments—as screening compounds. These small entities typically bind with relatively low affinity and activity to drug targets. Therefore, more sensitive screening methods than routine biochemical assays, e.g., using biophysical techniques, are often required to detect binding.^3,4^ While the throughput of these assays is limited, the chance of finding a fragment hit is considerably higher, as these compounds have a lower molecular complexity.^5^ Fragment libraries typically consists of a few thousand screening entities,^6,7^ and their construction should proceed with great care to ensure chemical diversity and appropriate (physico)chemical properties.^6,8^ Therefore, libraries are typically designed to broadly comply to the Rule of Three (Ro3), an empirical guideline that defines ranges of desirable physicochemical properties for fragments.^9,10^ Furthermore, it is important that (sub)structures that are associated with adverse properties such as assay interference (i.e., PAINS^11^), aggregation, and reactivity are avoided.^6^ Another key aspect in fragment library design is that any screening hit can be followed up by amenable chemistries that allow a swift and efficient hit to lead optimization; the extent to which a fragment lends itself for this process was recently coined “fragment sociability”.^6,12,13^ For this reason, it is important to develop the chemistries around the central scaffold of the fragment prior to compiling fragment screening libraries.

In recent years, the development of chemistries and libraries of 3D molecules has been receiving increasing attention.^14−17^ This trend is generally acknowledged to find its origin in the “Escape from Flatland” publications by Lovering et al., which showed that the fraction of sp^3^-hybridized carbon atoms (Fsp^3^) in drug candidates correlates with higher clinical success rates, improved solubility, and reduced promiscuity.^18,19^ It is noted that, despite the fact that Fsp^3^ remains a popular metric for assessing compound libraries, other metrics such as principal moment of inertia (PMI) and plane of best fit (PBF) are also frequently used to assess three-dimensionality; however, these two metrics do not necessarily correlate with Fsp^3^ and thus do not have the same predictive value.^14,20,21^ While most fragment libraries are currently dominated by flat molecules, the incorporation of 3D character into screening fragments would also ensure that the resulting leads and clinical candidates have this characteristic. Although it is widely acknowledged that the higher complexity of 3D screening compounds will reduce hit rates,^5^ potential advantages can be gained, including an increase in novelty and diversity for the resulting (hit) compounds.

To guide the library design process, a wide variety of computer-aided drug design (CADD) tools exists, including MOE,^22^ DataWarrior,^23^ and Pipeline Pilot.^24^ For our work, we aimed to develop a dedicated library design tool with the following requirements: (i) scalable, i.e., enabling the design of large diverse libraries but also small and focused sets of screening compounds; (ii) an open-soure computational workflow that can readily be shared; (iii) the ability to apply a wide variety of filters, for example, physchem properties, novelty, and costs; (iv) the ability to also consider 3D character of the representative compounds. To the best of our knowledge, such automated workflows for the design of (3D) fragment libraries have not been reported. Here we construct a workflow using the open-source platform Konstanz Information Miner (KNIME)^25^ to navigate the different properties and requirements during (3D) fragment library design. KNIME relies on so-called “nodes” that are assigned with specific tasks and that can be connected in sequence to construct a workflow comprising successive steps toward a final output. The availability of a broad spectrum of cheminformatics nodes—often freely available^26−31^—makes the platform particularly suitable for fragment library design^32^ and other medicinal chemistry purposes.^33−37^ Our workflow is made freely available and is readily customizable. It uniquely generates virtual combinatorial fragment libraries and subsequently selects compounds for synthesis based on diversity—as determined by calculated physicochemical properties and molecular fingerprints—while ensuring compound novelty, synthetic feasibility, and three-dimensional character. The workflow can be configured such that it can be employed for the design of larger libraries of 100–1000 compounds or for much smaller and focused sets of screening compounds to incorporate in structurally diverse fragment libraries. Here we describe the library design workflow and demonstrate the application in the design and subsequent synthesis of a small and focused library based on the cyclopropane scaffold, which we have previously identified to be underrepresented in our fragment library.^38^

The general workflow is divided into three stages: (i) the generation of a virtual library followed by (ii) novelty and shape analysis and (iii) compound selection from this library (Figure 1A). Many of the components of the workflow are modular and can be adjusted according to the preferences of the user. At the first stage, any reagent database(s) can be imported and subsequently reacted *in silico* with the predefined scaffold building block(s) in a combinatorial fashion by selecting the desired synthetic transformation from a predefined list. Any reactions that have not yet been configured in the current version of the workflow can be added by the user to the list in the form of a SMARTS query. Prior to this “reactor” step, appropriate reagents (i.e., having the required functional group for the specified transformation and no interfering functional groups) are selected, and if the imported reagent database(s) requires further curation, a broad filter on physicochemical properties can be specified. The filter should be kept broad to build in some margin for changes of properties after the *in silico* chemical transformation while allowing for removal of reagents that are predestined to be filtered out in later steps due to highly undesirable properties. Selection of the allowed protecting groups is also possible at this stage. Any reagent that bears one of the selected protecting groups is “virtually deprotected”, and this information is appended. This prevents skewing of calculations due to the presence of the protecting group that will not be present in the final fragment and allows selection of protecting groups that are compatible with the envisioned synthetic strategy. Finally, after the “reactor” step, the virtual library is curated further by removal of fragments with physicochemical properties outside the specified range (e.g., Ro3) as well as compounds that present PAINS motifs^11^ and/or bear undesirable functional groups.^39^

**Figure 1:**
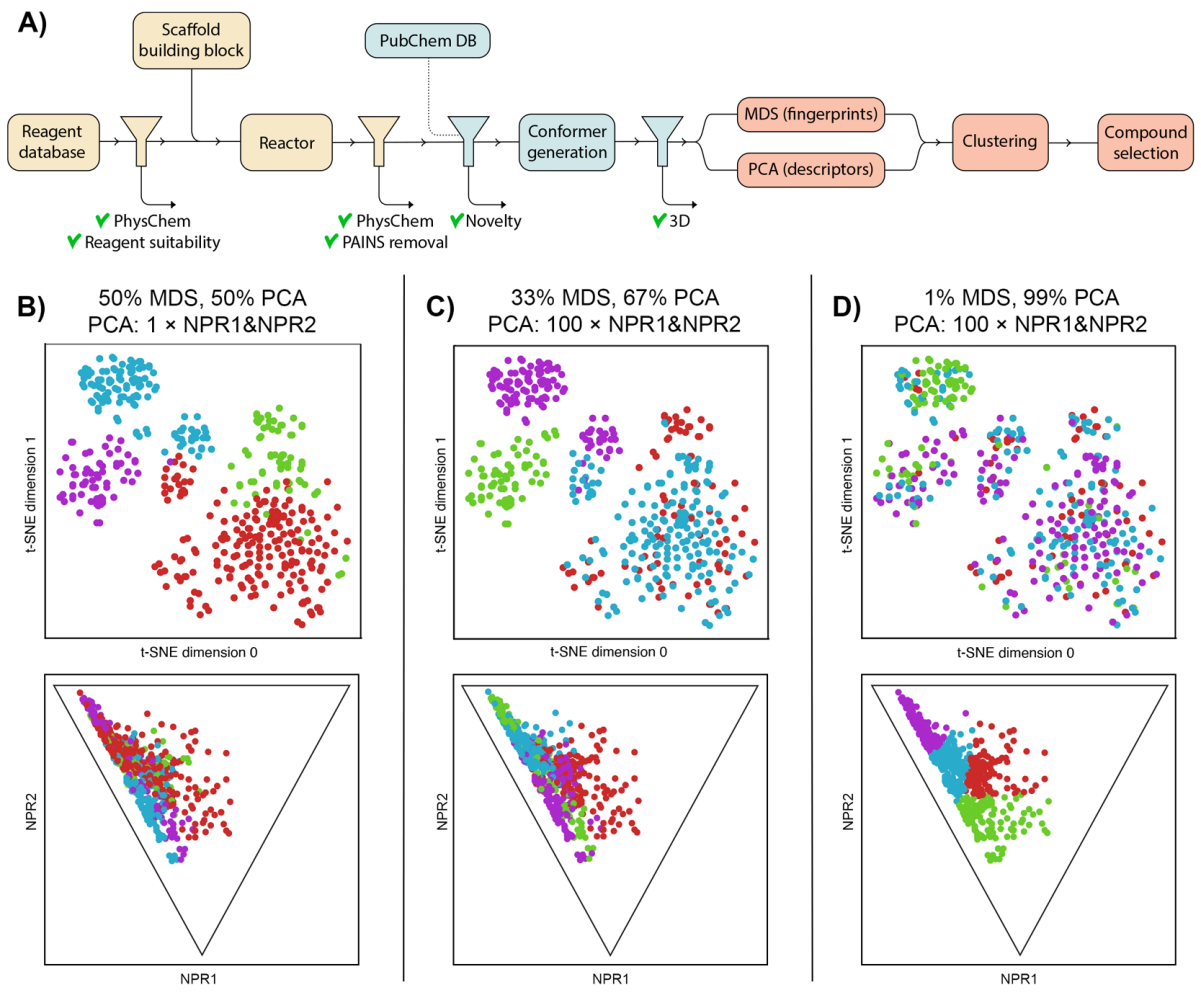
Overview of the workflow and clustering approach. (A) Schematic representation of the workflow. The three stages ((i) the generation of a virtual library, (ii) novelty and shape analysis, and (iii) compound selection from the library) are represented with different colors. (B–D) Exemplary comparison of three different settings in the clustering step where increasing weight is given to PMI values NPR1 and NPR2. In each panel, a set of compounds (i.e., the virtual library from which compounds **6a**–**f** were selected) was clustered into four clusters using MDS/PCA/*k*-means as described in the text. The top graph shows chemical space visualized by *t*-SNE from the calculated fingerprints, whereas the bottom graph shows the corresponding PMI plot. (B) PCA and MDS received equal weight; NPR1 and NPR2 did not receive extra weight. (C) PCA received twice the weight of MDS; NPR1 and NPR2 received 100-fold weight in PCA. Fingerprint diversity is largely conserved while clustering becomes more apparent in the PMI plot. (D) PCA received 100-fold weight; NPR1 and NPR2 received 100-fold weight. Clustering is effectively dominated by PMI values.

The second stage commences with retrieval of all molecules encompassing the common scaffold(s) of the generated library, or alternatively all molecules with a Tanimoto similarity score higher than the user-defined value, from the PubChem database using the PubChem Power User Gateway (PUG).^40^ The PubChem database includes >110 million unique compounds from different sources, including patents and literature. The retrieved compound database is then compared with the virtually generated library, and compounds that have already been reported (the sources can be selected, e.g., patents and/or publications) are marked for removal. Additionally, databases with (commercially) available compounds can also be fed into the workflow with the same purpose. This contributes to assuring novelty among compounds suggested by the workflow. Next, conformations up to a specified conformational energy are generated for the entire library using the RDKit conformer generator, and only diverse conformers are preserved. For the conformations generated, PMI and PBF values are calculated. Thereafter, the metric and cutoff value that will be used to filter for 3D character can be chosen, as well as whether the 3D values are calculated as averages of all conformers or from the single conformer with the lowest conformational energy. Frequently, only the global-minimum conformer is considered, but this is rarely the biologically relevant conformation,^41^ and—depending on the flexibility of the compound—different conformations of the same molecule can possess highly dissimilar PMI/PBF values. Therefore, careful consideration of these parameters is essential for an accurate assessment of the 3D character of the structures comprising the database.

With the resulting virtual library at hand, the final stage is concerned with compound selection from this library. In order to maximize diversity (and thus maximally cover the intended chemical space), both molecular descriptors and fingerprints were included in the clustering approach (for a comparison, see Figure S1).^42^ Tanimoto distances are calculated from the fingerprints and subjected to multidimensional scaling (MDS).^43^ Given the popularity^44,45^ of the *t*-distributed stochastic neighbor embedding (*t*-SNE) method for the purpose of dimensionality reduction, this was implemented as an alternative to MDS. The MDS (or *t*-SNE) dimensions are subsequently normalized to unit variance of the first dimension. Molecular descriptors are normalized using *Z*-score normalization, subjected to PCA, and also normalized to unit variance of the first component. By normalizing the MDS dimensions and PCA components to unit variance of the first dimension or component, molecular fingerprints and molecular descriptors receive equal weight in the subsequent clustering step.^42^ This approach allowed for the inclusion of 3D metric values in the PCA and subsequent clustering, with the option of giving increased weight to the (3D) properties. It is therefore possible to adjust the balance between fingerprint-based diversity and (3D-)descriptor-based diversity (as illustrated in Figure 1B–D). For clustering, the *k*-means algorithm was employed, although other algorithms can easily be implemented with the available KNIME nodes. Each compound is then assigned a “cluster score”, defined as the distance to the centroid of the corresponding cluster. We noticed that compounds with high cluster scores would typically bear substituents solely composed of carbon atoms. While these are arguably fair representatives for any given cluster and may indicate satisfactory clustering performance, selecting these centroids does not necessarily result in library members that are maximally diverse and attractive in terms of physicochemical properties. To introduce more variety in the physicochemical properties during the picking of cluster representatives, a secondary score is therefore assigned to each compound. This score is a weighed function of the number of hydrogen-bond donors (HBD), hydrogen-bond acceptors (HBA), number of aromatic rings, and halogen presence (1 or 0 for presence or absence, respectively) (see the Supporting Information for more details). All structures are ranked according to their cluster score and secondary score, and the relative weight of these two scores can be defined. From each cluster the compound with the highest summed ranking is selected.

To demonstrate the use of our workflow, we designed a set of fragments based on the 1,2-disubstituted cyclopropane scaffold. The principle of 3D fragments relies on scaffolds that are able to position exit vectors out of plane and into three-dimensional space. The rigidity and high sp^3^ character of small (hetero)aliphatic rings such as the cyclopropane ring allow for such geometries, and we have previously reported that these rings are underrepresented in our fragment library.^38^ Upon 1,2-disubstitution of the cyclopropane ring, *cis* and *trans* diastereomers can be obtained, which both possess distinct 3D properties due to the different relative orientations of the two exit vectors in the two respective isomers. As a result, the *cis* and *trans* isomers represent complementary scaffolds that serve as interesting starting points for 3D fragment synthesis. We therefore designed and subsequently synthesized a set of *cis*- and *trans*-1,2-disubstituted cyclopropanes. Recently, Chawner et al. also reported the synthesis and analysis of a focused 3D fragment library based on the cyclopropyl moiety,^46^ to which our work can be considered complementary in terms of the design approach and (properties of) fragments obtained.

We designed our library to consist of a first subset with various amide substituents on the “northern” exit vector and a second subset with various ether substituents on the “southern” exit vector (Figure 2A). The application of the workflow (for detailed settings, see the Supporting Information) resulted in the set of compounds in Table 1.

**Figure 2:**
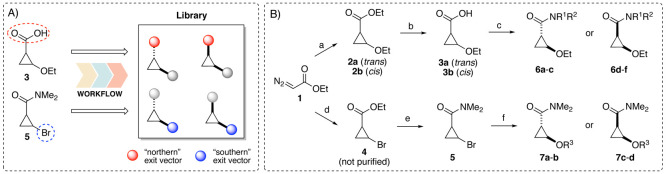
Design and synthesis of the cyclopropane library. (A) Building blocks **3** and **5** were decorated using the workflow by derivatization of the synthetic handles (dashed circles), resulting in a library of complementary *cis*- and *trans*-cyclopropane fragments with varying “northern” and “southern” exit vectors. (B) Synthetic routes toward the cyclopropane library: (a) ethyl vinyl ether, Rh_2_(OAc)_2_, Et_2_O, rt, 3.5 h, 31% (**2a**) and 40% (**2b**); (b) aq NaOH, EtOH, 80 °C, 1 h, 77–89%; (c) [i] HATU, DIPEA, R^1^R^2^NH, rt, overnight, 6–55%; [ii] for **6b** and **6e**: MeOH, H_2_O, 100 °C, MW irradiation, 8 h, 26–43% over two steps; (d) vinyl bromide, Rh_2_(OAc)_2_, DCE, rt, 3 days; (e) [i] aq NaOH, EtOH, 80 °C, 1 h; [ii] SOCl_2_, rt, overnight; [iii] Me_2_NH·HCl, DIPEA, THF, rt, 2.5 h, 12% from **1**; (f) KOH, 18-crown-6, R^3^OH, rt, 2 h, 3–62%. Except for **6b**, all final compounds are racemates.

**Table 1:**
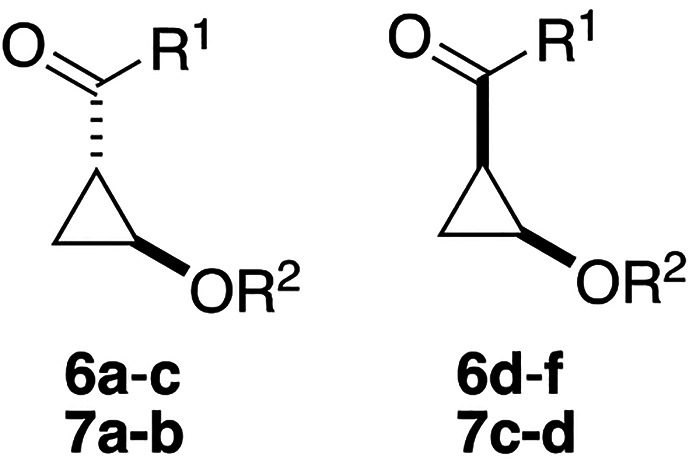

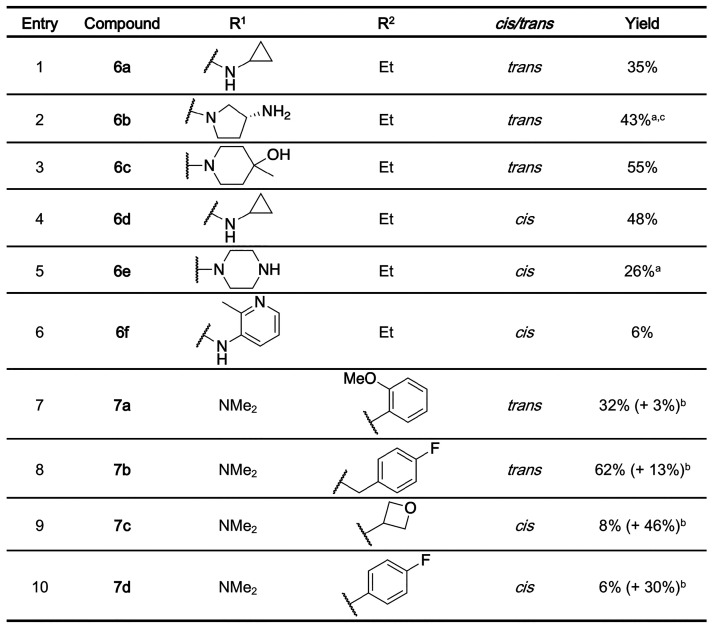
Compounds Comprising the Cyclopropane Library and the Corresponding Isolated Yields (Except for **6b**, All Final Compounds Are Racemates)

a Isolated yield over two steps including a Boc deprotection.

b The isolated yield for the opposite diastereomer is given between brackets.

c Obtained as a mixture of two enantiomerically pure diastereomers in a 1:1 ratio.

Synthesis of carboxamides **6a**–**f** started by a rhodium-catalyzed cyclopropanation from ethyl diazoacetate (**1**, EDA) and ethyl vinyl ether, leading to building blocks **2a** and **2b** in an approximate d.r. of 1:1.4 (^1^H NMR analysis of the concentrated reaction mixture) and isolated as the separated racemic *cis* and *trans* isomers in 31% and 40% yield, respectively (Figure 2B). Ester hydrolysis provided acids **3a** and **3b**, which subsequently afforded final compounds **6a**–**f** following HATU-mediated amide coupling and a Boc deprotection (where applicable). The projected amide couplings proceeded smoothly, except for **6f**. In fact, the workflow had initially suggested the 3-methylpyridin-2-ylamino rather than 2-methylpyridin-3-ylamino substituent, but following troublesome synthesis due to low conversion and side product formation, we reasoned that the second-best-scoring compound of that cluster (i.e., **6f**) might be easier to obtain due to the higher nucleophilicity of the amine. Although synthesis of this compound also proved difficult, we were able to obtain **6f**, albeit in low yield (Table 1, entry 6). Initial attempts toward Boc deprotection under acidic conditions (TFA/CH_2_Cl_2_, HCl/dioxane) resulted in opening of the cyclopropane ring, but utilization of a microwave-assisted approach using boiling water^47^ efficiently provided the unprotected amines without any signs of ring opening. The reactivity of the cyclopropane moiety under acidic conditions was not unexpected, as the combination of an electron-donating and an electron-withdrawing substituent is also leveraged in Lewis acid-catalyzed reactions with donor–acceptor cyclopropanes.^48^ Despite acid instability, the cyclopropanes reported here were unreactive under boiling water conditions and also showed no sign of instability in HBSS buffer for up to 4 weeks, indicating that the compounds are suitable for most fragment screening approaches.

Diversification of the southern exit vector was initially foreseen via alkylation of the alcohol counterpart of ethers **2** or **6**. However, during exploratory work this approach proved challenging, as deprotection of the corresponding benzyl ethers (H_2_, Pd/C) to the intended alcohol resulted in rapid opening of the ring. Therefore, we adapted an alternative approach reported by Banning et al.^49^ that allowed us to maintain a similar degree of “fragment sociability”.^13^ The route started with the synthesis of crude ester **4** from EDA and vinyl bromide, followed by ester hydrolysis, chlorination, and amidation to yield cyclopropane **5**. The final step comprised the projected formal nucleophilic substitution of bromocyclopropane **5** in the presence of 18-crown-6 ether and provided desired compounds **7a**–**d**. Interestingly, this step was reported not to proceed via direct substitution but rather via dehydrobromination and subsequent conjugate addition of the alcohol.^49,50^ As a consequence, the stereochemical identity of **5** (^1^H NMR analysis of the crude reaction mixtures of **4** and **5** both showed an approximate *cis*/*trans* ratio of 1:1.6) is not retained, and the *trans* isomer is formed predominantly. LCMS analysis of the crude reaction mixtures of **7a**–**d** indicated formation of the *cis* and *trans* isomers in approximate ratios of 1:5 to 1:10. Reversed-phase column chromatography ultimately provided the desired compounds—as well as the additional diastereomer—with high diastereomeric purity. Although this approach was sufficient for our small compound set, this strategy would require improvement for the synthesis of larger libraries of *cis*-cyclopropanes.

Analysis of the resulting library shows a favorable physicochemical profile with values spanning the lower ranges within the Ro3 limits for most of the calculated properties (Figure 3A,B). Due to the design of our fragments (see the Supporting Information), only the number of rotatable bonds (nRot) and topological polar surface area (TPSA) slightly exceed the proposed Ro3 cutoffs by maximum calculated values, and in terms of mean calculated values, only nRot exceeds the cutoff. To establish whether these favorable calculated physicochemical properties also translate into high aqueous solubility—a requirement for fragments to be able to be screened at high concentrations that are required in most fragment screening assays and one of the propagated advantages of 3D fragments—we experimentally determined aqueous solubility and/or aggregation by means of nephelometry (Figure 3C). The compounds with the lowest (**6e**) and highest (**7d**) cLogD_7.4_ values were included in these measurements, as well as a set of opposing diastereomers (**6a** and **6d**) that has cLogD values close to the mean value. The compound with the lowest cLogD value (**6e**) did not show any sign of precipitation or aggregation even at the maximum concentration tested (3 mM), and the compound with the highest cLogD value (**7d**) showed no sign of precipitation or aggregation up to 300 μM. Although a direct link cannot be made between cLogD and precipitation and/or aggregation, these measurements indicate that the set of compounds is suitable for screening at concentrations typically used in fragment screening assays. Comparison of the selected *trans* (**6a**) and *cis* (**6d**) compounds reveals that the *cis* isomer shows slightly better solubility than the *trans* isomer, as the *cis* isomer showed no precipitation or aggregation even at the highest concentration measured (3 mM) and the *trans* isomer showed no sign of precipitation or aggregation up to 1 mM. Although our data are limited, it is not unreasonable to postulate that slightly higher polarity of the *cis* isomer (as evidenced by lower retention on the C-18 column^51^) may result in slightly better solubility, despite the fact that both isomers have identical cLogD values (due to stereochemistry typically not being considered in these calculations).

**Figure 3:**
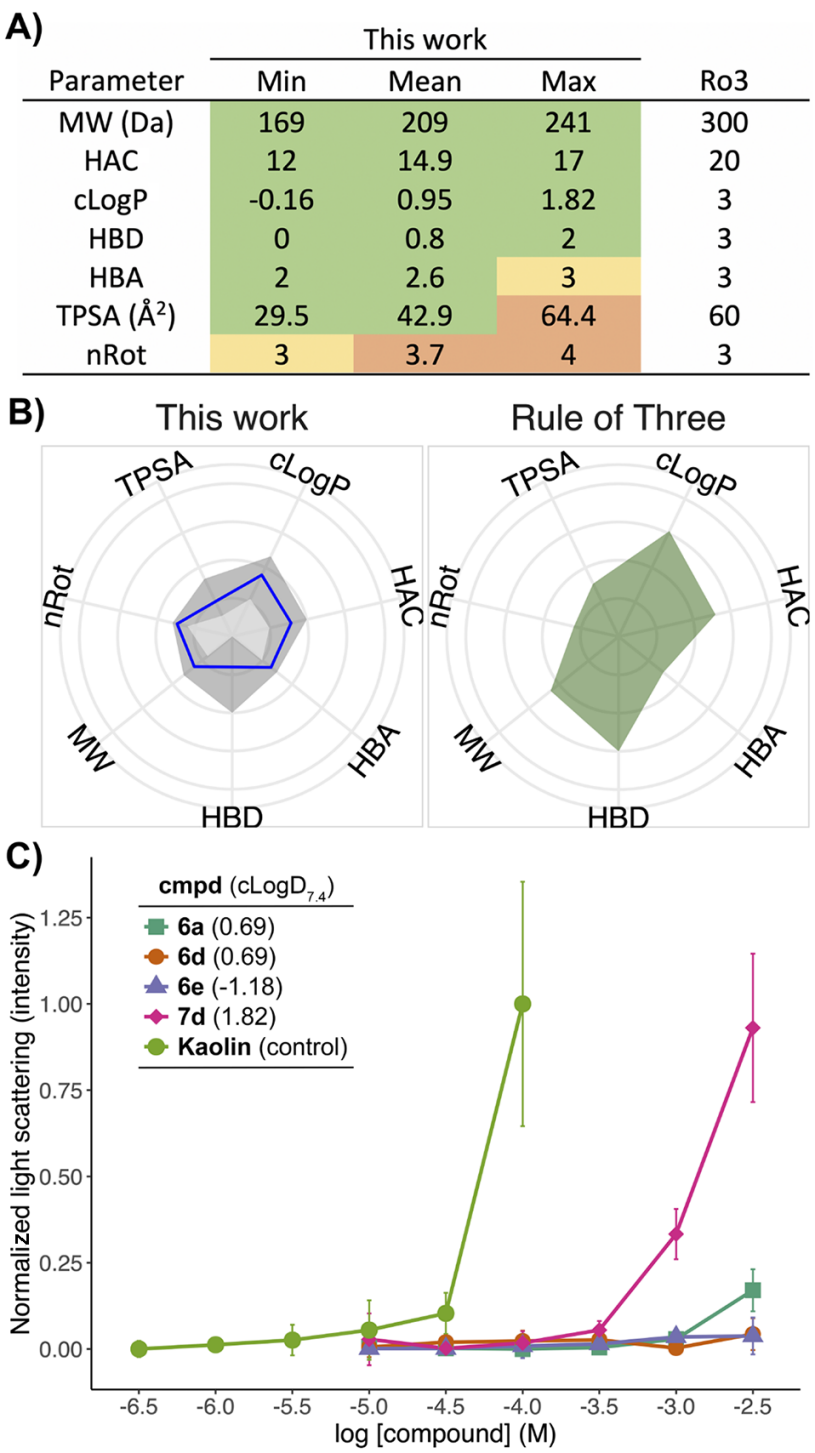
Analysis of the physicochemical properties of the cyclopropane library. (A) Calculated physicochemical values, expressed as minimum, mean, and maximum values. Cells are colored according to their adherence to the Ro3 (right-most column, supplemented with a heavy-atom count (HAC) limit of ≤20): higher (orange), equal to (yellow), or lower (green) than the Ro3 limits. (B) Radar plots depicting the distribution of physicochemical properties of the cyclopropane library and the Ro3 limits (in an identical representation as in our recent 3D fragment libraries review^14^). Axes were scaled as follows:^14^ cLogP, [−1.9; 4.5]; HAC, [7; 27]; HBA, [0; 8]; HBD, [0; 4]; MW, [95; 455 Da]; nRot, [0; 10]; TPSA, [10; 140 Å^2^]. Mean values are depicted by the blue line. Ranges, defined by the minimum and maximum values, are defined by the gray areas. (C) Solubility/aggregation analysis of selected fragments by nephelometry.

Next, a PMI plot was generated to analyze the three-dimensional character of the library (Figure 4A). As per design, all compounds possess average ∑NPR values above the 1.07 cutoff proposed by Firth et al.^21^ The anticipated complementarity of the *cis* and *trans* scaffolds also becomes apparent in the PMI analysis, as the two sets of diastereomers populate different areas in the plot. Closer analysis of the generated conformations of one set of opposing diastereomers (i.e., **6a** and **6d**; Figure 4B,C) shows that the *trans* isomer adopts a more linear conformation in comparison to the *cis* isomer, consistent with the PMI analysis that indicated a more rodlike and disclike character for the *trans* and *cis* isomers, respectively. This also explains the slightly higher average ∑NPR value for the *cis* series, that as a result had a higher percentage of compounds passing the 3D filter (∑NPR ≥ 1.07) in the workflow; 84% and 55% of generated *cis* and *trans* compounds in the virtual library passed the filter, respectively (Figure S2). Although the *cis* scaffold is thus considered to generally have a higher 3D character in terms of PMI, it could be argued that it is shape complementarity, rather than high ∑NPR values alone, that should play a key role in 3D fragment library design. A comparison of the current library with synthetic^14,16^ and commercial^52^ 3D/Fsp^3^-rich fragment libraries shows that it fares well in terms of shape diversity and that it possesses moderate ∑NPR values (Figure 4D).

**Figure 4:**
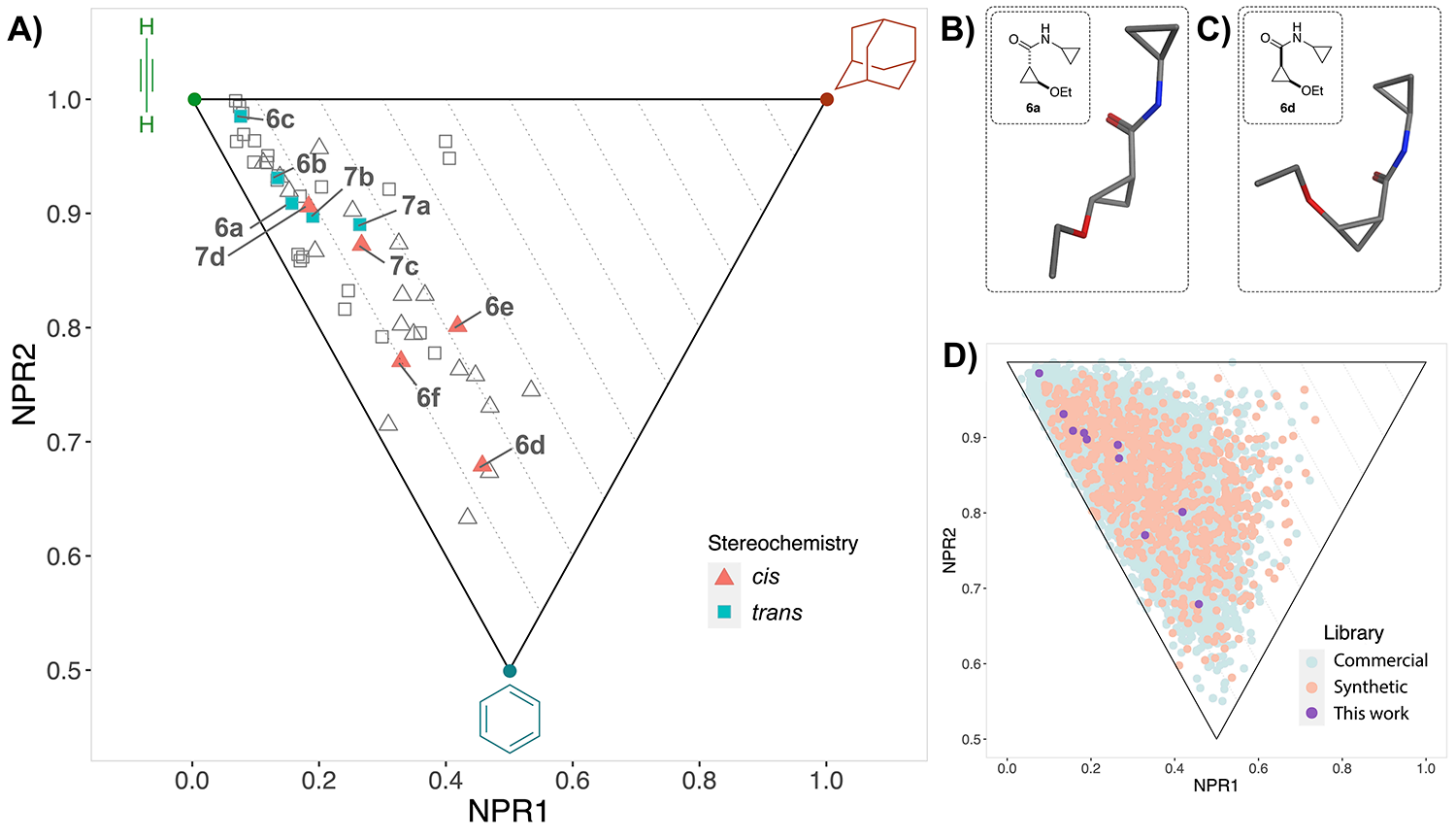
Shape analysis of the cyclopropane library using the commonly used PMI plots^20^ (e.g., Chawner et al.^46^). (A) PMI plot of the cyclopropane library. Open data points represent PMI values of individual conformations (Δ*E*_max_ ≤ 5 kcal·mol^–1^, RMSD > 0.1). Average PMI values per compound are plotted as solid data points. (B, C) Global minimum conformations of opposing diastereomers **6a** and **6d**, made with MOE software (v2019.0104). (D) PMI-based comparison of the current library to synthetic^14,16^ and commercial^52^ 3D/Fsp^3^-rich fragment libraries.

In conclusion, we have constructed an automated workflow for the design of 3D fragment libraries. This workflow ensures that novel and three-dimensional fragments are selected as library members while also ensuring diversity in terms of physicochemical properties, molecular fingerprints, and shape. We demonstrated its application in the design of a focused 10-membered fragment library based on the cyclopropane scaffold, which we have previously shown to be underrepresented in our libraries.^38^ The designed set comprised novel and diverse *cis*- and *trans*-1,2-disubstituted cyclopropanes, for which PMI analysis showed complementarity in terms of shape due to the different relative orientations of the exit vectors in the two respective isomers. Analysis of both calculated and experimentally determined physicochemical properties indicate that the library fares well in this regard, spanning the lower ranges within the Ro3 limits and showing aqueous solubility at concentrations relevant for fragment screening assays. The workflow can be broadly applied to designing small to large compound libraries.

## Supporting Information Available

- Computational methods, experimental methods, Figures S1 and S2, URL to KNIME workflow, and ^1^H and ^13^C NMR spectra (PDF)

## Abbreviations

3D: three-dimensional
CADD: computer-aided drug design
cLogD: calculated log *D*
cLogP: calculated log *P*
EDA: ethyl diazoacetate
FBDD: fragment-based drug design
Fsp^3^: fraction of sp^3^-hybridized carbon atoms
HATU: *O*-(7-azabenzotriazol-1-yl)-*N*,*N*,*N*′,*N*′-tetramethyluronium hexafluorophosphate
HAC: heavy-atom count
HBA: hydrogen-bond acceptor
HBD: hydrogen-bond donor
HBSS: Hanks’ Balanced Salt Solution
HTS: high-throughput screening
KNIME: Konstanz Information Miner
MDS: multidimensional scaling
MOE: Molecular Operating Environment
MW: molecular weight
nRot: number of rotatable bonds
PAINS: pan-assay interference compounds
PBF: plane of best fit
PCA: principal component analysis
PMI: principal moment of inertia
PUG: PubChem Power User Gateway
Ro3: Rule of Three
TPSA: topological polar surface area
*t*-SNE: *t*-distributed stochastic neighbor embedding
