# Supplementary material for: An Automated, Open-Source Workflow for the Generation of (3D) Fragment Libraries
Source: ACS Med Chem Lett. 2023 May 2;14(5):583–90. doi: 10.1021/acsmedchemlett.2c00503 (PMC10184156; doi:10.1021/acsmedchemlett.2c00503)

SUPPORTING INFORMATION

An automated, open-source workflow for the generation of (3D) fragment libraries

Tom Dekker†, Mathilde A.C.H. Janssen‡, Christina Sutherland†, Rene W.M. Aben‡, Hans W. Scheeren‡, Daniel Blanco-Ania‡, Floris P.J.T. Rutjes‡, Maikel Wijtmans† and Iwan J.P. de Esch†,*

† Amsterdam Institute of Molecular and Life Sciences (AIMMS), Vrije Universiteit Amsterdam, De Boelelaan 1108, 1081 HZ Amsterdam, The Netherlands.
‡ Institute for Molecules and Materials, Radboud University, Heyendaalseweg 135, 6525 AJ Nijmegen, The Netherlands

* Corresponding author: Iwan J.P. de Esch; Email: i.de.esch@vu.nl

Table of Contents

General **S2**
Computational methods **S2**
  Figure S1 .... **S2**
  Settings used in workflow S3
  Secondary scoring function S3
  Figure S2 .... **S4**
Experimental methods **S5**
  Nephelometry S5
  Synthetic methods S5
^{1}H- and ^{13}C-NMR spectra of intermediates and final compounds **S14**

General

Figures were made in Adobe Illustrator 2021, MOE v2019.0104, ChemDraw v20.0 or v21.0, and RStudio 2022.02.2 (Build 485) with the ggplot2 package installed.

Computational methods

The workflow was constructed in KNIME v4.3.1 on a 2019 Apple iMac (5K Retina, 3.6 GHz 8-core Intel Core i9, 64 GB RAM, Radeon Pro 580X 8 GB) running MacOS Cataline v10.15.1. RDKit (4.0.1.v202006261025), Erlwood (v4.0.0) and Vernalis (1.28.2.v202101281353) KNIME nodes were used. Additional features were added via Python scripts running Python v3.6.12 in Anaconda v2020.11, with OpenBabel v2.8.1 and RDKit v2020.03.06. For specific details about the workflow, see the KNIME. The workflow can be downloaded from: https://hub.knime.com/tomdekker/spaces/(3D)%20Fragment%20Library%20Design%20Workflow/latest.

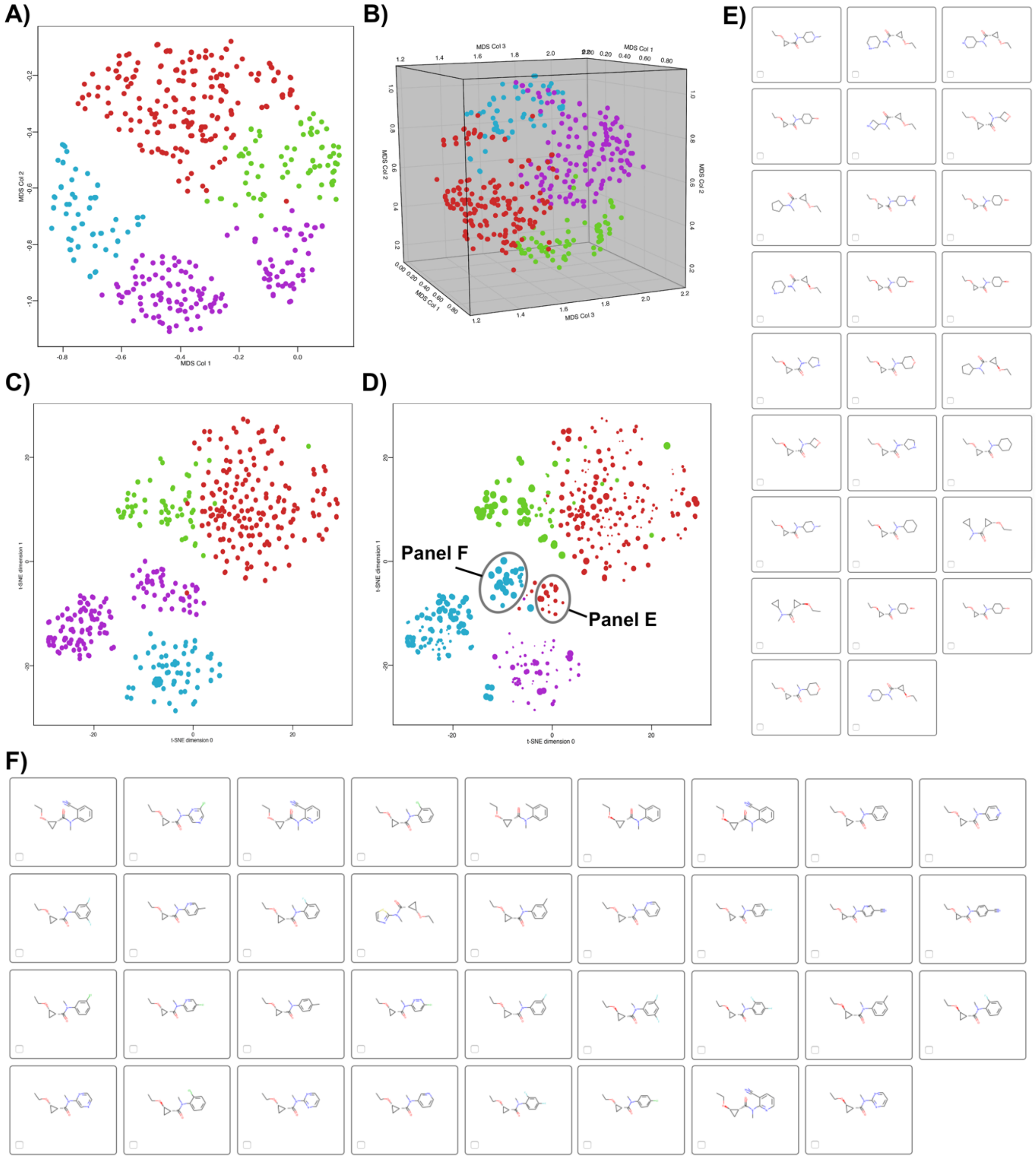

Figure S1. Exemplary depiction/comparison of the clustering method. (A) The virtual library from which **6a–f** were selected was clustered into four clusters using k-means with merely the fingerprints (by reducing the Tanimoto distance matrix into

20 MDS dimensions) as input, and without input from PCA (i.e., chemical descriptors). Colors represent the different clusters. The compounds were plotted using (a separate) MDS in only two dimensions. A significant degree of the information captured in the fingerprints is captured in two dimensions with respect to what is captured in 20 dimensions. (B) Data and clustering identical to panel A, but plotted using MDS in three dimensions. Clustering becomes more apparent. (C) Data and clustering identical to panel A–B, but here the fingerprints are reduced using t-SNE. Contrary to MDS, t-SNE is non-linear, with inter-cluster distance being less relevant and hence clusters becoming more apparent in only few dimensions. When panel A–B and C are compared, it becomes apparent that both methods capture a similar degree of information. (D) Data identical to panel A–C, but the compounds were clustered using the combined MDS/PCA approach (i.e., chemical descriptors were included). A single cluster that was identified by the approaches A–C, is now split into two separate clusters (encircled data points), due to their different physicochemical properties captured in the PCA components. For clarity, the size of the data points corresponds to size of the first PCA component, from which it becomes apparent that the red cluster generally possesses lower values for the first component. This component has significant covariance (coefficient of 0.4–0.5) with sLogP, MW, and HAC. The encircled compounds comprising these two clusters are depicted in Panel (E) and (F).

Settings used in workflow

We designed our library to consist of a first subset with various amide substituents on the "northern" exit vector and a second subset with various ether substituents on the "southern" exit vector (Figure 2A). The first subset was based on amidation of acid **3** and was designed to include three amide compounds per diastereomer type. The second subset involved substitution on bromide **5** by alcohols and comprised two ether compounds per diastereomer type. The corresponding synthetic transformations and building blocks were imported into the workflow, along with our in-house reagent database and a database of commercially available reagents. The latter was filtered to only include relatively inexpensive reagents (≤200 €/g) in order to limit complexity and expenses. Following generation of the virtual combinatorial libraries, filtering was applied on the maximum number of rotatable bonds (nRot; 4), molecular weight (MW; 280 Da), hydrogen bond acceptors (HBA; 4) and donors (HBD; 4), polar surface area (TPSA; 70 $Å^2$), cLogP (3) and the number of aromatic rings (1). Furthermore, a maximum of one or zero additional specified stereocenters was allowed in the first and second subset, respectively, allowing for the use of enantiopure reagents in the first subset only and thereby balancing complexity between subsets. Some of the physicochemical properties (i.e., nRot and TPSA) were deliberately allowed to exceed the Ro3 limits, as there has been debate whether these Ro3 limits are too strictly formulated.[1,2] Using an expanded Ro3 would thereby allow for a broader and more diverse set of fragments. Next, compounds were removed based on occurrence in patents, articles and commercial libraries. To bias for 3D character, diverse conformations (RMSD >0.1) were generated up to 5 kcal·mol^{-1} above the global minimum, and compounds with an average ΣNPR lower than 1.07 (the 3D cut-off proposed by Firth *et al.*[3]) were removed. Clustering was performed per building block/exit vector combination, i.e., **6a**–**c**, **6d**–**f**, **7a**,**b** and **7c**,**d** result from four individual clusterings. Morgan fingerprints (1024 bits, radius = 2) and the outlined molecular descriptors (see main text) were calculated, and subsequently included in MDS and PCA, respectively, where MDS and PCA received equal weighing. All molecular descriptors received equal weight in the PCA. MDS dimensions and PCA components were limited to the number of dimensions that ensured a stress value of <0.05 and an explained variance of >90%, respectively, and used as input for the k-means clustering algorithm. From each cluster, the highest-ranked compound was selected for synthesis, with priority given to in-house reagents up to the 5th ranking compound. See Figure S2 for more details.

Secondary scoring function

The secondary scoring was defined as:
|(1.75*NumHBD+1.25*NumHBA+1*NumAromaticRings+1*has_halogen)/(NumHeavyAtoms)-(SS_balance_value)|
The balancing value (SS_balance_value, default value=0.375) can be changed to include more (higher value) or less (lower value) "interaction". Its weighing with respect to the cluster score can also be configured.

A)

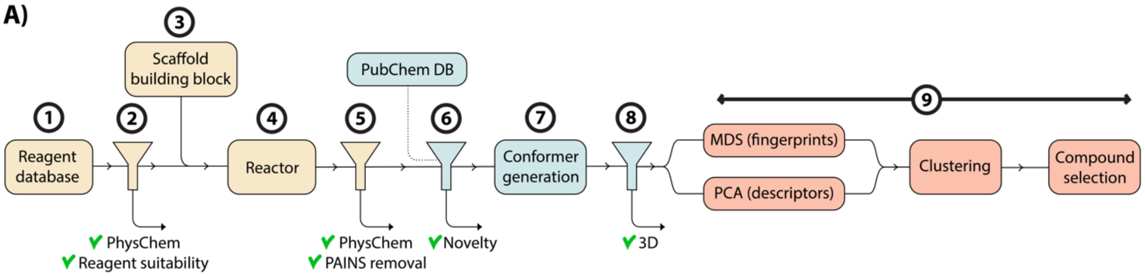

B)

	#1	#2	#3		#4	#5	#6	#7	#8	#9
6a-c	85,963 reagents	4,837 reagents R–NH_2	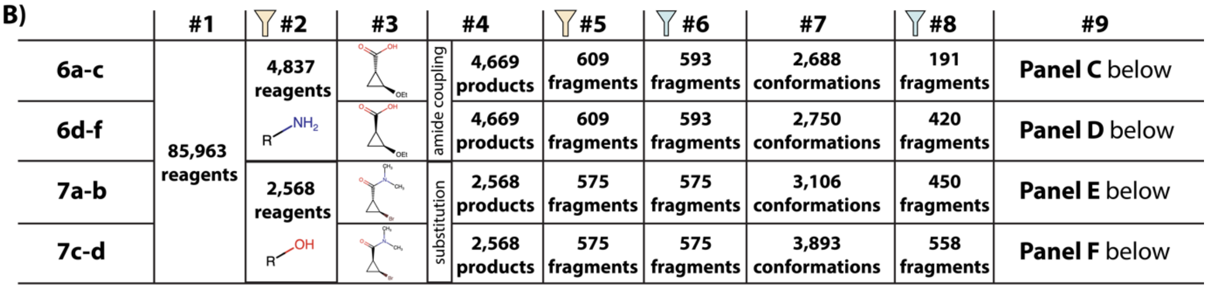	amide coupling	4,669 products	609 fragments	593 fragments	2,688 conformations	191 fragments	**Panel C** below
6d-f					4,669 products	609 fragments	593 fragments	2,750 conformations	420 fragments	**Panel D** below
7a-b		2,568 reagents R–OH		substitution	2,568 products	575 fragments	575 fragments	3,106 conformations	450 fragments	**Panel E** below
7c-d					2,568 products	575 fragments	575 fragments	3,893 conformations	558 fragments	**Panel F** below

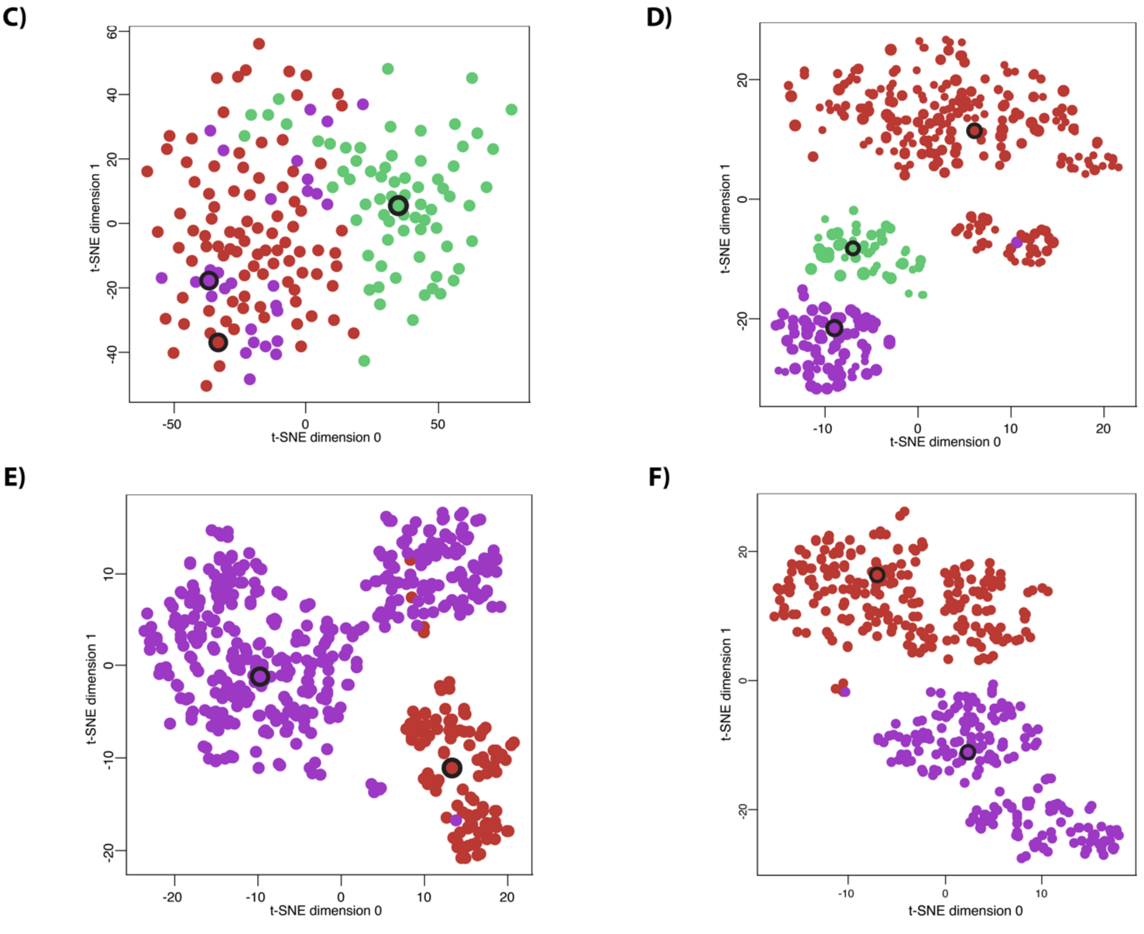

Figure S2. Details on the design of the focused library (**6a–f**, **7a-d**). (A) General overview of the workflow with the different steps being numbered. (B) Table with the output of the different elements/steps as numbered in panel A. (C-F) Clustering and compound selection as referred to in column #9 in panel B. Clustering was performed as described above (i.e., using the combined MDS/PCA/k-means approach) and visualized using two-dimensional t-SNE reduction of the fingerprints. Colors represent the different clusters; compounds selected for synthesis (see main text) are encircled with black.

Experimental methods

Nephelometry

Nephelometry was performed using BMG LABTECH NEPHELOstar Plus equipment. Kaolin was used as the internal standard and compounds were added to HBSS buffer as DMSO stock solution to a final concentration of 1% DMSO and a total volume of 200 µL. Precipitation or aggregation was considered significant when average values exceeded three times the standard deviation of the blanks. Blank values were omitted if they exceeded three times the standard deviation of the 24 blanks that were measured on each 96-well plate. All compounds were tested in triplo and wells of suspected outliers were visually inspected before omitting any outliers. Data was processed in Excel v16.16.27 for MacOS and graphs were made in RStudio 2022.02.2 (Build 485) running the ggplot2 package.

Synthetic methods

General

All reagents have been purchased from commercial suppliers (primarily being Sigma-Aldrich and Combi-Blocks) and used without further purification. THF and DMF were dried by passing through an activated alumina column prior to use. All other solvents used were used as received unless otherwise stated. Hygroscopic reagents (18-crown-6 ether, dimethylamine hydrochloride) were dried by co-evaporation with MeCN prior to use. TLC analyses were performed using Screening Devices or Merck F254 aluminum-backed silica plates and visualized with 254 nm UV light or staining with $KMnO_4$.

LC-MS analysis was carried out on a Shimadzu LC-20AD liquid chromatograph pump system with a Shimadzu M20A photodiode array detector, a Shimadzu LCMS2010EV mass spectrometer and Xbridge C18 column (5 µm, 4.6 × 50 mm) at 40 °C using ESI in positive ion mode. For acidic runs, 0.1% HCOOH in H_2O and 0.1% HCOOH in MeCN were used as eluent A and B, respectively. For basic runs, 0.4% w/v NH_4HCO_3 in H_2O and MeCN and were used as eluent A and B, respectively. The gradient for acidic and basic runs was 5:90:90:5:5% B at t = 0:4.5:6:6.5:8 min. The purity of a compound was determined by calculating the peak area percentage of UV detection at 200 nm. The purity of compounds bearing aromatic groups was also assessed at 230 nm and 254 nm, and such values were reported if the purity was lower at these wavelengths than at 200 nm; Unless stated otherwise, the reported purity was measured at 200 nm. HRMS spectra were determined with a Bruker micrOTOF mass spectrometer using ESI in positive ion mode. Reverse phase column chromatography was performed on Teledyne ISCO CombiFlash Rf 200 equipment with the same solvent systems used for LC-MS measurements. Normal phase flash chromatography was performed on Biotage Isolera or BUCHI Pure C-815 equipment. Preparative HPLC was performed on BUCHI PrepChrom C-700 purification system equipped with a XBridge Prep C18 (5 µm, 19 × 100 mm) column. Pre-packed columns were purchased from Screening Devices (C18 and UltraPure irregular silica) or BUCHI (FlashPure EcoFlex irregular silica). Microwave reactions were carried out using a Biotage Initiator. IUPAC names were generated with ChemDraw Professional 21.0 (PerkinElmer).

Nuclear magnetic resonance (NMR) spectra were determined with a Bruker Avance II 500 MHz or a Bruker Avance III HD 600 MHz spectrometer. Chemical shifts are reported in parts per million (ppm) against the reference compound using the signal of the residual non-deuterated solvent ($CDCl_3$ δ = 7.26 ppm (^1H), δ = 77.16 ppm (^{13}C); DMSO-d_6 δ = 2.50 ppm (^1H), δ = 39.52 ppm (^{13}C); CD_3OD δ = 3.31 ppm (^1H), δ = 49.00 ppm (^{13}C)). NMR spectra were processed using MestReNova 14.0 software. The peak multiplicities are defined as follows: s, singlet; d, doublet; t, triplet; q, quartet; dd, doublet of doublets; ddd, doublet of doublets of doublets; dt, doublet of triplets; dq, doublet of quartets; td, triplet of doublets; tt, triplet of triplets; qd, quartet of doublets; p, pentet; dp, doublet of pentets; br, broad signal; m, multiplet. For NMR listings, in addition to specific instructions that are given by the journal in the guidelines for authors the following additional procedures were used: 1) Multiplicity is not solely reported based on peak shapes, but also distinguishes the coupling to all non-equivalent protons that have similar *J* values; 2) If additional smaller couplings are observed but are too small for accurate quantitation because the precision is smaller than the digital resolution, a symbol ∆ will be used; 3)

The notation 'm' is used in case of obscured accurate interpretation as a result of (i) overlapping signals for different protons, or (ii) a result of overlapping signal lines within the same proton signal; 4) For any rotamers or diastereomers, signals will be listed separately if resolved; 5) NMR signals that could only be detected with HSQC analysis are denoted with a # symbol; 6) NMR signals that could only be detected with HMBC analysis are denoted with a * symbol; 7) If one or more signals remain undetected after extensive 1D and 2D NMR analyses, this will be mentioned. 8) Signals for exchangeable proton atoms (such as NH and OH groups) are only listed if clearly visible (e.g., excluding the use of D_2O or CD_3OD) and if confirmed by a D_2O shake and/or HSQC.

SAFETY STATEMENT

Bromoethene is volatile (b.p. 16 °C) and a possible carcinogen. The appropriate safety measures were used as described in the experimental procedure. No further unexpected or unusually high safety hazards were encountered.

General procedure A – Ester hydrolysis

Aq. KOH (5.0 M, 2.0 eq) was added to a solution of the ester (1.0 eq) in EtOH (0.2 M). The solution was stirred at 80 °C for 1 h in a capped microwave vial. The reaction mixture was diluted with water, transferred to a separatory funnel, neutralized with 1.0 M HCl and extracted thrice with EtOAc. The combined organic phases were dried over Na_2SO_4, filtered and concentrated *in vacuo* to give the desired acid that was of sufficient purity for further reactions.

General procedure B – Amide coupling

HATU (1.2 eq.) and DIPEA (3.0 eq) were added to a solution of a carboxylic acid (1.0 eq) in dry DMF (0.25 M). The reaction vial was flushed with nitrogen gas and the mixture was stirred at rt for 1 h. The amine (1.2 eq) was added. The reaction mixture was stirred overnight at rt and partitioned between sat. aq. Na_2CO_3 (25 mL) and EtOAc (25 mL). The phases were separated, and the aqueous phase was extracted with EtOAc (2 × 25 mL). The combined organic phases were washed with brine, dried over Na_2SO_4, filtered and concentrated under reduced pressure. Subsequent purification by column chromatography or preparative HPLC provided the desired amide.

General procedure C – Boc deprotection

The Boc-protected amine was dissolved in MeOH (1.0 mL). The mixture was diluted with water to 0.03 M and heated at 100 °C for 8 h under microwave irradiation. The reaction mixture was concentrated under reduced pressure. The residue was subjected to reverse phase chromatography (basic mode, 5–50% B). Lyophilization of the relevant fractions provided the desired amine.

General procedure D – Substitution

This procedure was adapted from Banning *et al.*[4] To a vial charged with powdered KOH (3.0 eq), 18-crown-6 (0.10 eq) and the alcohol (2.0 eq), was added a solution of bromocyclopropane **5** (1.0 eq) in THF (0.2 M). The mixture was stirred at rt for 2 h, after which the reaction mixture was partitioned between EtOAc and sat. aq. $NaHCO_3$. The phases were separated, and the aqueous layer was extracted twice with EtOAc. The combined organic layers were dried over Na_2SO_4, filtered and concentrated *in vacuo*. Column chromatography yielded the separated *trans*- and *cis*-isomers.

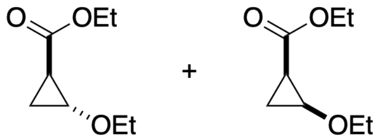

***rac*-Ethyl (1*R*,2*R*)-2-ethoxycyclopropane-1-carboxylate (2a)** and ***rac*-ethyl (1*R*,2*S*)-2-ethoxycyclopropane-1-carboxylate (2b)**

Ethyl vinyl ether (2.40 mL, 25.6 mmol) was dissolved in Et_2O (9 mL) and $Rh_2(OAc)_4$ (12 mg, 28 µmol, 0.2 mol%) was added. The mixture was stirred at rt while a solution of ethyl diazoacetate (1.61 g, 14.1 mmol) in PhMe (12 mL) was added with a syringe pump at a flow rate of 5 mL/h. The solution was stirred for an additional 1 h at rt and subsequently concentrated *in vacuo*. The residue was subjected to normal phase column chromatography (0–15% EtOAc in cHex) to yield the *trans*-diastereomer **2a** as a colorless oil (900 mg, 40%) and the crude *cis*-diastereomer that was purified further with Kugelrohr distillation to yield the *cis*-diastereomer **2b** as a colorless oil (700 mg, 31%). Relative stereochemistry was assigned based on the J_{sum} of the CH-proton(s) on the cyclopropane ring, which is larger for the *cis* isomer in comparison to the *trans* isomer.[5]

trans-Isomer: **^{1}H NMR** (500 MHz, $CDCl_3$) δ 4.17 – 4.06 (m, 2H), 3.67 – 3.52 (m, 3H), 1.75 (ddd, *J* = 9.5, 6.0, 2.0 Hz, 1H), 1.30 – 1.21 (m, 5H), 1.19 (dd, *J* = 7.0, 7.0 Hz, 3H). **^{13}C NMR** (126 MHz, $CDCl_3$) δ 172.9, 66.8, 60.6, 60.5, 21.2, 15.8, 15.1, 14.4. **LC-MS** (acidic): t_R: 3.88 min, purity: >99%, $(M + H)^+$: calc. for $C_8H_{14}O_3$: 159, found: 159.

cis-Isomer: **^{1}H NMR** (500 MHz, $CDCl_3$) δ 4.22 – 4.10 (m, 2H), 3.66 – 3.51 (m, 2H), 3.44 (dq, *J* = 9.5, 7.1 Hz, 1H), 1.69 (ddd, *J* = 8.6, 6.6, 6.6 Hz, 1H), 1.55 (ddd, *J* = 6.5, 6.4, 4.8 Hz, 1H), 1.26 (dd, *J* = 7.1, 7.1 Hz, 3H), 1.18 (dd, *J* = 7.1, 7.1 Hz, 3H), 1.06 (ddd, *J* = 8.6, 6.4, 6.4 Hz, 1H). **^{13}C NMR** (126 MHz, $CDCl_3$) δ 170.3, 66.9, 60.6, 59.0, 20.8, 15.0, 14.4, 13.2. **LC-MS** (acidic): t_R: 3.48 min, purity: 96%, $(M + H)^+$: calc. for $C_8H_{14}O_3$: 159, found: 159.

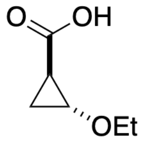

***rac*-(1*R*,2*R*)-2-Ethoxycyclopropane-1-carboxylic acid (3a)**

General procedure A with ester **2a** (300 mg, 1.90 mmol) yielded the title compound as a pale-yellow oil (190 mg, 77%). **^{1}H NMR** (500 MHz, DMSO) δ 3.58 – 3.44 (m, 3H), 1.60 (ddd, *J* = 9.5, 5.9, 2.0 Hz, 1H), 1.14 (ddd, *J* = 9.5, 5.3, 4.2 Hz, 1H), 1.10 (dd, *J* = 7.0, 7.0 Hz, 3H), 1.05 (ddd, *J* = 6.7, 5.6, 5.6 Hz, 1H). **^{13}C NMR** (126 MHz, DMSO) δ 173.3, 65.7, 59.6, 20.4, 14.8, 14.7.

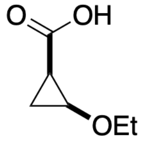

***rac*-(1*R*,2*S*)-2-Ethoxycyclopropane-1-carboxylic acid (3b)**

General procedure A with ester **2b** (300 mg, 1.90 mmol) yielded the title compound as a pale-yellow oil (220 mg, 89%). **^{1}H NMR** (500 MHz, DMSO) δ 3.55 (ddd, *J* = 6.7, 6.7, 4.7 Hz, 1H), 3.49 (dq, *J* = 9.6, 7.0 Hz, 1H), 3.41 – 3.35 (m, 1H), 1.59 (ddd, *J* = 8.4, 6.7, 6.7 Hz, 1H), 1.21 (ddd, *J* = 6.6, 5.1, 5.1 Hz, 1H), 1.06 (dd, *J* = 7.0, 7.0 Hz, 3H), 0.97 (ddd, *J* = 8.4, 6.6, 5.5 Hz, 1H). **^{13}C NMR** (126 MHz, DMSO-d_6) δ 170.9, 65.7, 58.3, 20.1, 14.9, 12.3.

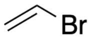

Bromoethene (S1)

Safety warning: Bromoethene is volatile (b.p. 16 °C) and a possible carcinogen. It was kept in solution and handled in a fume hood at all times.

This procedure was adapted from Huang *et al*.[6] A 250 mL reaction flask was charged with powdered KOH (12.7 g, 226 mmol) and EtOH (90 mL). The flask was equipped with a

Vigreux condenser connected to a 100 mL collecting flask. The collecting flask was charged with DCE (3.0 mL) and cooled with liquid N_2. The reaction mixture was stirred at 45 °C and $BrCH_2CH_2Br$ (15.0 mL, 174 mmol) was added dropwise over the course of 1 h. After an additional 2 h at 65 °C, 8.1 g of vinyl bromide (76 mmol, 44%) in DCE was obtained as calculated by ^{1}H NMR peak integration relative to DCE. This solution was used directly in the next step. **^{1}H NMR** (500 MHz, $CDCl_3$) δ 6.45 (dd, J = 15.0, 7.1 Hz, 1H), 5.99 (dd, J = 7.1, 1.9 Hz, 1H), 5.86 (dd, J = 15.0, 1.9 Hz, 1H)

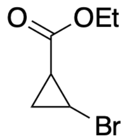

Ethyl 2-bromocyclopropane-1-carboxylate (4)

The catalyst $Rh_2(OAc)_4$ (59 mg, 13 µmol, 0.25 mol %) was added to a flask equipped with a dry ice condenser and charged with bromoethane **S1** (8.1 g, 76 mmol) in DCE (3.0 mL) *(Safety warning: see experimental procedure above)*. The mixture was cooled to −10 °C using an ethylene glycol/dry ice bath. A solution of ethyl diazoacetate (6.0 g, 53 mmol) in PhMe (45 mL) was added with a syringe pump at a flow rate of 1 mL/h. After 20 h, the flow rate was adjusted to 0.5 mL/h. After complete addition, the reaction mixture was filtered over diatomaceous earth and concentrated *in vacuo* *(Safety warning: see experimental procedure above)* to yield a dark yellow oil (6.3 g) that was used in the next step without purification.

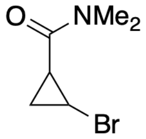

2-Bromo-*N*,*N*-dimethylcyclopropane-1-carboxamide (5)

This procedure was adapted from Prosser *et al*.[7] A solution of the crude ester **4** (1.5 g) in aq. 1.0 M NaOH (12 mL) was heated at reflux for 30 min. The solution was cooled and partioned between Et_2O (50 mL) and 1.0 M NaOH (40 mL). The aqueous layer was neutralised with 3.0 M HCl and extracted with Et_2O (3 × 50 mL). The combined organic layers were dried over Na_2SO_4, filtered and concentrated *in vacuo* to give a dark yellow oil (900 mg). To 800 mg of this oil was added $SOCl_2$ (0.80 mL, 11 mmol), and the resulting mixture was stirred at rt overnight. Subsequently, the suspension was transferred to a stirring suspension of $Me_2NH{\cdot}HCl$ (0.99 g, 12 mmol) and DIPEA (4.2 mL, 24 mmol) in THF (16 mL) at rt. The mixture was allowed to stir at rt for 2.5 h, after which it was partitioned between EtOAc (50 mL) and 0.5 M HCl (50 mL). The layers were separated and the aqueous layer was extracted with EtOAc (2 × 50 mL). The combined organic layers were dried over Na_2SO_4, filtered and concentrated *in vacuo*. Kugelrohr distillation of the residue (8 mbar, 190 °C) yielded the title compound as a yellow oil as a mixture of diastereomers in an approximate *cis*/*trans* ratio of 1:1.6 (300 mg combined, 1.56 mmol, 12% extrapolated yield from **1**).

trans-Isomer: ^{1}H NMR (500 MHz, DMSO-d_6) δ 3.22 (ddd, J = 7.7, 4.6, 3.2 Hz, J_{sum} = 15.5 Hz, 1H), 3.13 (s, 3H), 2.83 (s, 3H), 2.43 (ddd, J = 9.3, 6.0, 3.2 Hz, 1H), 1.41 (ddd, J = 7.5, 5.8, 5.8 Hz, 1H), 1.31 (ddd, J = 9.4, 5.6, 4.6 Hz, 1H).

cis-Isomer: ^{1}H NMR (500 MHz, DMSO-d_6) δ 3.45 (ddd, J = 7.9, 6.7, 5.5 Hz, J_{sum} = 20.1 Hz, 1H), 3.08 (s, 3H), 2.87 (s, 3H), 2.33–2.27 (m, 1H), 1.35–1.28 (m, 2H).

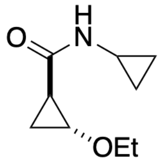

***rac*-(1*R*,2*R*)-*N*-Cyclopropyl-2-ethoxycyclopropane-1-carboxamide (6a)**

General procedure B with carboxylic acid **3a** (50 mg, 0.38 mmol) and cyclopropylamine followed by normal phase column chromatography (25–45% EtOAc in cHex) yielded the title compound as a white solid (23 mg, 35%). **^{1}H NMR** (600 MHz, CD_3OD) δ 3.59–3.53 (m, 2H), 3.50 (ddd, *J* = 6.4, 4.0, 2.1 Hz, 1H), 2.63 (tt, *J* = 7.3, 3.9 Hz, 1H), 1.64 (ddd, *J* = 9.6, 5.9, 2.1 Hz, 1H), 1.19–1.12 (m, 4H), 1.06 (ddd, *J* = 9.4, 5.4, 4.0 Hz, 1H), 0.75–0.66 (m, 2H), 0.52–0.44 (m, 2H). **^{13}C NMR** (151 MHz, CD_3OD) δ 175.7, 67.3, 60.3, 23.4, 23.0, 15.3, 14.7, 6.5, 6.4. **LC-MS** (acidic): t_R: 2.51 min, purity: >99%, $(M + H)^+$: 170. **HRMS**: $(M + Na)^+$ calc. for $C_9H_{15}NO_2$: 192.0995, found: 192.1001.

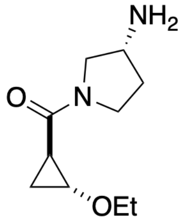

((*R*)-3-Aminopyrrolidin-1-yl)((1*R*,2*R*)-2-ethoxycyclopropyl)methanone and **((*R*)-3-aminopyrrolidin-1-yl)((1*S*,2*S*)-2-ethoxycyclopropyl)methanone (6b)**

General procedure B with carboxylic acid **3a** (100 mg, 0.77 mmol) and (*R*)-3-(Boc-amino)pyrrolidine followed by normal phase column chromatography (0–100% EtOAc in cHex) yielded the corresponding protected amide, which was deprotected following general procedure C to yield the title compound as a colorless oil (60 mg, 39%). Obtained as a mixture of two enantiomerically pure diastereomers in an approximate ratio of 1:1 (^{1}H NMR) that could not be fully discriminated due to overlap of signals, including those of additional rotamers/conformers.

^{1}H NMR (500 MHz, CD_3OD) δ 3.91–3.82 (m, 2H), 3.78–3.67 (m, 1H), 3.66–3.50 (m, 10H), 3.46–3.35 (m, 2H), 3.22–3.15 (m, 1H), 2.26–2.15 (m, 1H), 2.15–2.05 (m, 1H), 1.95–1.81 (m, 3H), 1.79–1.68 (m, 1H), 1.27–1.12 (m, 10H). **^{13}C NMR** (126 MHz, CD_3OD) δ 172.5, 172.3, 67.6, 67.6, 67.6, 61.3, 61.3, 61.2, 55.5, 55.4, 54.5, 52.4, 52.4, 50.8, 46.3, 45.5, 45.5, 34.8, 33.5, 21.8, 21.8, 21.5, 21.5, 15.5, 15.4, 15.4, 15.4, 15.3. **LC-MS** (basic): t_R: 2.08 min, purity: >99%, $(M + H)^+$: 199. **HRMS**: $(M + Na)^+$ calc. for $C_{10}H_{18}N_2O_2$: 221.1260, found: 221.1254.

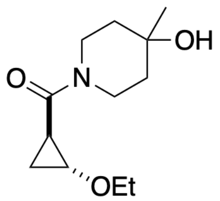

***rac*-((1*R*,2*R*)-2-Ethoxycyclopropyl)(4-hydroxy-4-methylpiperidin-1-yl)methanone (6c)**

General procedure B with carboxylic acid **3a** (50 mg, 0.38 mmol) and 4-methylpiperidin-4-ol followed by preparative HPLC (acidic mode, 5-95% B) and lyophilization of the relevant fractions yielded the title compound as a pale-yellow oil (48 mg, 55%). In NMR analysis, extra signals were observed due to rotamers/conformers.

^{1}H NMR (600 MHz, CD_3OD) δ 4.10–4.00 (m, 1H), 4.00–3.90 (m, 1H), 3.63–3.54 (m, 3H), 3.50 (ddd, *J* = 6.4, 4.0, 2.1 Hz, 1H), 3.24–3.14 (m, 1H), 2.12 (ddd, *J* = 9.5, 5.9, 2.1 Hz, 1H), 1.71–1.64 (m, 1H), 1.64–1.54 (m, 2H), 1.53–1.44 (m, 1H), 1.28–1.22 (m, 3H), 1.21–1.16 (m, 4H), 1.16–1.11 (m, 1H). **^{13}C NMR** (151 MHz, CD_3OD) δ 171.9, 171.8, 68.4, 67.5, 67.4, 61.2, 43.43, 43.40, 40.05, 39.98, 39.91, 39.89, 39.0, 29.92, 29.87, 20.1, 15.36, 15.34, 15.32. **LC-MS** (acidic): t_R: 2.58 min, purity: >99%, $(M + H)^+$: 228. **HRMS**: $(M + H)^+$ calc. for $C_{12}H_{21}NO_3$: 228.1594, found: 228.1595.

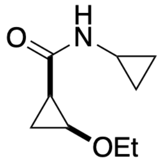

***rac*-(1*R*,2*S*)-*N*-Cyclopropyl-2-ethoxycyclopropane-1-carboxamide (6d)**
General procedure B with carboxylic acid **3b** (50 mg, 0.38 mmol) and cyclopropylamine followed by preparative HPLC (acidic mode, 5-95% B) and lyophilization of the relevant fractions yielded the title compound as a white solid (31 mg, 48%).
^{1}H NMR (600 MHz, CD_3OD) δ 3.56 (dq, *J* = 9.4, 7.0 Hz, 1H), 3.51 (ddd, *J* = 6.6, 6.6, 4.5 Hz, 1H), 3.46 (dq, *J* = 9.4, 7.0 Hz, 1H), 2.64 (tt, *J* = 7.3, 3.9 Hz, 1H), 1.57 (ddd, *J* = 9.0, 6.6 Hz, 1H), 1.38 (ddd, *J* = 6.8, 6.0, 4.5 Hz, 1H), 1.15 (dd, *J* = 7.1, 7.1 Hz, 3H), 0.98 (ddd, *J* = 9.0, 6.6, 6.0 Hz, 1H), 0.74–0.66 (m, 2H), 0.50–0.42 (m, 2H). **^{13}C NMR** (151 MHz, CD_3OD) δ 173.8, 67.6, 59.1, 23.5, 22.6, 15.2, 12.2, 6.5, 6.5. **LC-MS** (acidic): t_R: 2.38 min, purity: >99%, $(M + H)^+$: 170. **HRMS**: $(M + Na)^+$ calc. for $C_9H_{15}NO_2$: 192.0995, found: 192.1001.

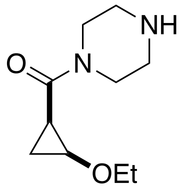

***rac*-((1*R*,2*S*)-2-Ethoxycyclopropyl)(piperazin-1-yl)methanone (6e)**
General procedure B with carboxylic acid **3b** (100 mg, 0.77 mmol) and 1-Boc-piperazine followed by normal phase column chromatography (50-100% EtOAc in cHex) yielded the corresponding protected amide, which was deprotected following general procedure C to yield the title compound as a colorless oil (40 mg, 26%). In NMR analysis, extra signals were observed due to rotamers/conformers.
^{1}H NMR (500 MHz, CD_3OD) δ 3.86–3.65 (m, 3H), 3.61 (ddd, *J* = 6.5, 6.5, 4.1 Hz, 1H), 3.58–3.43 (m, 3H), 2.98–2.73 (m, 4H), 1.95 (ddd, *J* = 8.8, 6.8, 6.8 Hz, 1H), 1.37 (ddd, *J* = 6.3, 6.2, 4.1 Hz, 1H), 1.14 (dd, *J* = 7.1, 7.1 Hz, 3H), 0.92 (ddd, *J* = 8.8, 6.0, 6.0 Hz, 1H). **^{13}C NMR** (126 MHz, CD_3OD) δ 169.7, 67.4, 58.4, 47.1, 46.6, 46.1, 43.6, 21.6, 15.3, 11.4. **LC-MS** (basic): t_R: 1.90 min, purity: >99%, $(M + H)^+$: 199. **HRMS**: $(M + Na)^+$ calc. for $C_{10}H_{18}N_2O_2$: 221.1260, found: 221.1251.

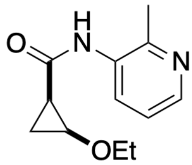

***rac*-(1*R*,2*S*)-2-Ethoxy-*N*-(2-methylpyridin-3-yl)cyclopropane-1-carboxamide (6f)**
General procedure B with carboxylic acid **3b** (75 mg, 0.47 mmol) and 2-methylpyridin-3-amine followed by normal phase column chromatography (0-100% EtOAc in cHex), reverse phase column chromatography (acidic mode, 3-50% B) and lyophilization of the relevant fractions yielded the title compound as a pale-yellow solid (6 mg, 6%).
^{1}H NMR (600 MHz, $CDCl_3$) δ 8.37 (d^{Δ}, *J* = 8.3 Hz, 1H), 8.22 (dd, *J* = 4.8, 1.7 Hz, 1H), 8.21 (s, 1H), 7.14 (dd^{Δ}, *J* = 8.2, 4.8 Hz, 1H), 3.83–3.71 (m, 2H), 3.65 (ddd, *J* = 6.2, 6.2, 4.4 Hz, 1H), 2.46 (s, 3H), 1.76 (ddd, *J* = 10.5, 6.7, 6.7 Hz, 1H), 1.38–1.27 (m, 4H), 1.21 (ddd, *J* = 6.8, 6.7, 4.5 Hz, 1H). **^{13}C NMR** (151 MHz, $CDCl_3$) δ 170.8, 148.0, 144.4, 133.0, 128.9, 121.9, 67.8, 58.0, 22.7, 21.1, 15.14, 15.13. **LC-MS** (acidic): t_R: 1.62 min, purity: >99%, $[M+H]^+$: 221. **HRMS**: $(M + H)^+$ calc. for $C_{12}H_{16}N_2O_2$: 221.1285, found: 221.1277.

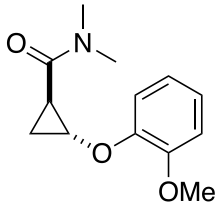

***rac*-(1*R*,2*R*)-2-(2-Methoxyphenoxy)-*N*,*N*-dimethylcyclopropane-1-carboxamide (7a)**

General procedure D with 2-methoxyphenol followed by normal phase column chromatography (0–3% MeOH in CH_2Cl_2), reverse phase column chromatography (acidic mode, 5–95% B) and lyophilization of the relevant fractions provided the title compound as a colorless oil (12 mg, 32%) and the corresponding *cis*-diastereomer as a colorless oil (1 mg, 3%).

Desired *trans*-isomer: **^{1}H NMR** (500 MHz, $CDCl_3$) δ 7.12 (dd, *J* = 7.8, 1.8 Hz, 1H), 7.00–6.87 (m, 3H), 4.14 (ddd, *J* = 6.3, 3.9, 2.2 Hz, 1H), 3.86 (s, 3H), 3.14 (s, 3H), 3.00 (s, 3H), 2.14 (ddd, *J* = 9.7, 6.1, 2.2 Hz, 1H), 1.55–1.49 (m, 1H), 1.45 (ddd, *J* = 9.6, 5.6, 3.9 Hz, 1H). **^{13}C NMR** (126 MHz, $CDCl_3$) δ 170.9, 149.0, 147.8, 121.9, 121.0, 113.8, 111.7, 58.1, 56.0, 37.5, 35.9, 20.0, 15.8. **LC-MS** (acidic): t_R: 3.34 min, purity: >99%, $(M + H)^+$: 236. **HRMS**: $(M + H)^+$ calc. for $C_{13}H_{17}NO_3$: 236.1281, found: 236.1281.

Corresponding *cis*-isomer: **^{1}H NMR** (600 MHz, $CDCl_3$) δ 7.07–7.03 (m, 1H), 6.95 (ddd, *J* = 7.9, 7.8, 1.7 Hz, 1H), 6.89–6.84 (m, 2H), 4.19 (ddd, *J* = 6.6, 6.6, 4.1 Hz, 1H), 3.81 (s, 3H), 3.09 (s, 3H), 2.92 (s, 3H), 2.00 (ddd, *J* = 9.0, 6.8, 6.8 Hz, 1H), 1.80 (ddd, *J* = 6.6, 6.5, 4.1 Hz, 1H), 1.13 (ddd, *J* = 9.0, 6.3, 6.3 Hz, 1H). **^{13}C NMR** (151 MHz, $CDCl_3$) δ 122.6, 120.9, 116.5, 113.3, 56.6, 56.3, 37.3, 35.9, 21.4, 11.7. (three missing signals). **LC-MS** (acidic): t_R: 3.18 min, purity: 93.0%*, $[M+H]^+$: 236. **HRMS**: $(M + H)^+$ calc. for $C_{13}H_{17}NO_3$: 236.1281, found: 236.1274. *Main impurity is the *trans*-diastereomer (LCMS, NMR)

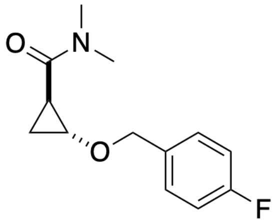

***rac*-(1*R*,2*R*)-2-((4-Fluorobenzyl)oxy)-*N*,*N*-dimethylcyclopropane-1-carboxamide (7b)**

General procedure D with (4-fluorophenyl)methanol followed by normal phase column chromatography (10-85% EtOAc in cHex) provided the title compound as a colorless oil (23 mg, 62%) and the corresponding *cis* diastereomer as a colorless oil (5 mg, 13%).

Desired *trans*-isomer: **^{1}H NMR** (500 MHz, $CDCl_3$) δ 7.36–7.27 (m, 2H), 7.07–6.98 (m, 2H), 4.57 (d, *J* = 11.5 Hz, 1H), 4.49 (d, *J* = 11.6 Hz, 1H), 3.67 (ddd, *J* = 6.3, 3.9, 2.1 Hz, 1H), 3.08 (s, 3H), 2.94 (s, 3H), 1.91 (ddd, *J* = 9.5, 5.8, 2.1 Hz, 1H), 1.32–1.25 (m, 1H), 1.17 (ddd, *J* = 9.3, 5.2, 3.9 Hz, 1H). **^{13}C NMR** (126 MHz, $CDCl_3$) δ 171.4, 162.6 (d, *J* = 246.1 Hz), 133.5 (d, *J* = 3.2 Hz), 130.0 (d, *J* = 8.2 Hz), 115.5 (d, *J* = 21.5 Hz), 72.7, 60.7, 37.4, 35.8, 19.7, 15.3. **LC-MS** (acidic): t_R: 3.62 min, purity: 96.2%, $(M + H)^+$: 238. **HRMS**: $(M + H)^+$ calc. for $C_{13}H_{16}FNO_2$: 238.1238, found: 238.1230.

Corresponding *cis*-isomer: **^{1}H NMR** (600 MHz, $CDCl_3$) δ 7.33–7.27 (m, 2H), 7.05–6.97 (m, 2H), 4.52 (d, *J* = 11.7 Hz, 1H), 4.41 (d, *J* = 11.7 Hz, 1H), 3.58 (ddd, *J* = 6.6, 6.6, 4.2 Hz, 1H), 3.14 (s, 3H), 2.99 (s, 3H), 1.82 (ddd, *J* = 8.9, 6.7, 6.7 Hz, 1H), 1.66–1.63 (m, 1H), 0.91 (ddd, *J* = 8.9, 6.1, 6.1 Hz, 1H). **^{13}C NMR** (151 MHz, $CDCl_3$) δ 168.3, 162.5 (d, *J* = 245.8 Hz), 133.6 (d, *J* = 3.2 Hz), 129.9 (d, *J* = 8.2 Hz), 115.3 (d, *J* = 21.3 Hz), 72.5, 57.3, 37.3, 35.9, 21.4, 11.2. **LC-MS** (acidic): t_R: 3.44 min, purity: 91.4%*, $(M + H)^+$: 238. **HRMS**: $(M + H)^+$ calc. for $C_{13}H_{16}FNO_2$: 238.1238, found: 238.1228. *Main impurity is the *trans*-diastereomer (LCMS, NMR).

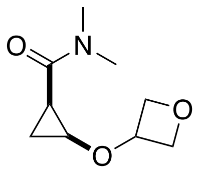

***rac*-(1*R*,2*S*)-*N*,*N*-Dimethyl-2-(oxetan-3-yloxy)cyclopropane-1-carboxamide (7c)**
General procedure D with oxetan-3-ol followed by normal phase column chromatography (10-60% EtOAc in cHex) provided the title compound as a colorless oil (7 mg, 8%). Fractions containing the corresponding *trans*-isomer were pooled and subjected to further purification with reverse phase column chromatography (acidic mode, 5-95% B). Lyophilization of the relevant fractions yielded the *trans*-isomer as a colorless oil (40 mg, 46%).
Desired *cis*-isomer: **^{1}H NMR** (600 MHz, $CDCl_3$) δ 4.78–4.66 (m, 2H), 4.66–4.54 (m, 3H), 3.54 (ddd, *J* = 6.6, 6.6, 4.1 Hz, 1H), 3.16 (s, 3H), 2.99 (s, 3H), 1.81 (ddd, *J* = 9.0, 6.8, 6.8 Hz, 1H), 1.53 (ddd, *J* = 6.4, 6.3, 4.1 Hz, 1H), 0.88 (ddd, *J* = 8.9, 6.1, 6.1 Hz, 1H). **^{13}C NMR** (151 MHz, CDCl3) δ 167.9, 78.7, 78.5, 72.7, 56.2, 37.2, 35.9, 21.1, 10.8. **LC-MS** (acidic): t_R: 1.93 min, purity: >99%, $(M + H)^+$: 186. **HRMS**: $(M + Na)^+$ calc. for $C_9H_{15}NO_3$: 208.0944, found: 208.0935.
Corresponding *trans*-isomer: **^{1}H NMR** (500 MHz, $CDCl_3$) δ 4.81–4.74 (m, 2H), 4.70–4.58 (m, 3H), 3.55 (ddd, *J* = 6.3, 3.9, 2.1 Hz, 1H), 3.16 (s, 3H), 2.94 (s, 3H), 1.96 (ddd, *J* = 9.6, 5.9, 2.2 Hz, 1H), 1.28–1.21 (m, 1H), 1.14 (ddd, *J* = 9.4, 5.2, 3.9 Hz, 1H). **^{13}C NMR** (126 MHz, $CDCl_3$) δ 171.1, 78.7, 78.5, 72.7, 59.0, 37.5, 35.8, 19.2, 15.1. **LC-MS** (acidic): t_R: 2.16 min, purity: >99%, $(M + H)^+$: 186. **HRMS**: $(M + Na)^+$ calc. for $C_9H_{15}NO_3$: 208.0944, found: 208.0939.

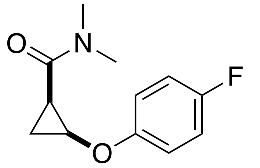

***rac*-(1*R*,2*S*)-2-(4-Fluorophenoxy)-*N*,*N*-dimethylcyclopropane-1-carboxamide (7d)**
General procedure D with 4-fluorophenol followed by normal phase column chromatography (0–3% MeOH in CH_2Cl_2), reverse phase column chromatography (acidic mode, 5–95% B) and lyophilization of the relevant fractions provided the title compound as a white solid (6 mg, 6%) and the corresponding *trans* isomer as a colorless oil (31 mg, 30%).
Desired *cis*-isomer: **^{1}H NMR** (600 MHz, $CDCl_3$) δ 6.98–6.91 (m, 4H), 4.05–3.98 (m, 1H), 3.12 (s, 3H), 2.93 (s, 3H), 2.04 (ddd, *J* = 9.0, 6.9, 6.9 Hz, 1H), 1.73–1.67 (m, 1H), 1.17 (ddd, *J* = 9.0, 6.2, 6.2 Hz, 1H). **^{13}C NMR** (151 MHz, $CDCl_3$) δ 167.3, 157.9 (d, *J* = 238.9 Hz), 154.7 (d, *J* = 2.3 Hz), 116.7 (d, *J* = 8.2 Hz), 115.8 (d, *J* = 23.2 Hz), 55.5, 37.3, 35.9, 21.2, 11.7. **LC-MS** (acidic): t_R: 3.35 min, purity: >99%, $(M + H)^+$: 224. **HRMS**: $(M + H)^+$ calc. for $C_{12}H_{14}FNO_2$: 224.1081, found: 224.1073.
Corresponding *trans*-isomer: **^{1}H NMR** (600 MHz, $CDCl_3$) δ 7.01–6.94 (m, 2H), 6.94–6.88 (m, 2H), 4.07 (ddd, *J* = 6.3, 3.8, 2.2 Hz, 1H), 3.15 (s, 3H), 3.00 (s, 3H), 2.04 (ddd, *J* = 9.8, 6.1, 2.2 Hz, 1H), 1.50 (ddd, *J* = 6.3, 6.3, 5.5 Hz, 1H), 1.34 (ddd, *J* = 9.5, 5.5, 3.8 Hz, 1H). **^{13}C NMR** (151 MHz, $CDCl_3$) δ 170.7, 157.8 (d, *J* = 238.9 Hz), 154.5 (d, *J* = 2.1 Hz), 116.1 (d, *J* = 23.2 Hz), 115.9 (d, *J* = 8.1 Hz), 57.9, 37.5, 35.9, 20.0, 15.6. **LC-MS** (acidic): t_R: 3.75 min, purity: >99%, $(M + H)^+$: 224. **HRMS**: $(M + H)^+$ calc. for $C_{12}H_{14}FNO_2$: 224.1081, found: 224.1074.

References

(1) Jhoti, H.; Williams, G.; Rees, D. C.; Murray, C. W. The 'Rule of Three' for Fragment-Based Drug Discovery: Where Are We Now? *Nat. Rev. Drug Discov.* **2013**, *12* (8), 644. https://doi.org/10.1038/nrd3926-c1.

(2) Köster, H.; Craan, T.; Brass, S.; Herhaus, C.; Zentgraf, M.; Neumann, L.; Heine, A.; Klebe, G. A Small Nonrule of 3 Compatible Fragment Library Provides High Hit Rate of Endothiapepsin Crystal Structures with Various Fragment Chemotypes. *J. Med. Chem.* **2011**, *54* (22), 7784–7796. https://doi.org/10.1021/jm200642w.

(3) Firth, N. C.; Brown, N.; Blagg, J. Plane of Best Fit: A Novel Method to Characterize the Three-Dimensionality of Molecules. *J. Chem. Inf. Model.* **2012**, *52* (10), 2516–2525. https://doi.org/10.1021/ci300293f.

(4) Banning, J. E.; Prosser, A. R.; Rubin, M. Thermodynamic Control of Diastereoselectivity in the Formal Nucleophilic Substitution of Bromocyclopropanes. *Org. Lett.* **2010**, *12* (7), 1488–1491. https://doi.org/10.1021/ol100187c.

(5) Wiberg, K. B.; Nist, B. J. The Nuclear Magnetic Resonance Spectra of Cyclopropane Derivatives. *J. Am. Chem. Soc.* **1963**, *85* (18), 2788–2790. https://doi.org/10.1021/ja00901a026.

(6) Huang, H.-M.; Bellotti, P.; Daniliuc, C. G.; Glorius, F. Radical Carbonyl Propargylation by Dual Catalysis. *Angew. Chem. Int. Ed.* **2021**, *60* (5), 2464–2471. https://doi.org/10.1002/anie.202011996.

(7) Prosser, A. R.; Banning, J. E.; Rubina, M.; Rubin, M. Formal Nucleophilic Substitution of Bromocyclopropanes with Amides En Route to Conformationally Constrained β-Amino Acid Derivatives. *Org. Lett.* **2010**, *12* (18), 3968–3971. https://doi.org/10.1021/ol101228k.

^{1}H- and ^{13}C-NMR spectra of intermediates and final compounds

^{1}H-NMR spectrum of **2a**

^{1}H NMR (500 MHz, $CDCl_3$) δ 4.17 – 4.06 (m, 2H), 3.67 – 3.52 (m, 3H), 1.75 (ddd, J = 9.5, 6.0, 2.0 Hz, 1H), 1.30 – 1.21 (m, 5H), 1.19 (dd, J = 7.0, 7.0 Hz, 3H).

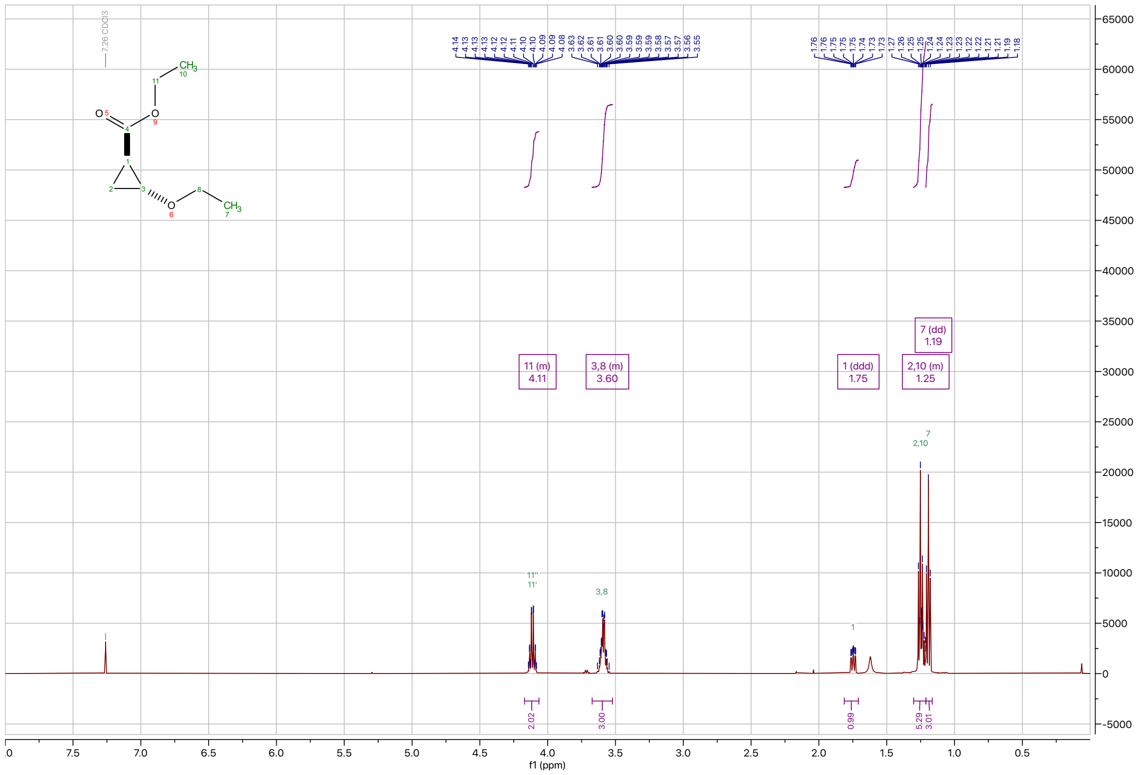

^{13}C-NMR spectrum of **2a**

^{13}C NMR (126 MHz, $CDCl_3$) δ 172.9, 66.8, 60.6, 60.5, 21.2, 15.8, 15.1, 14.4.

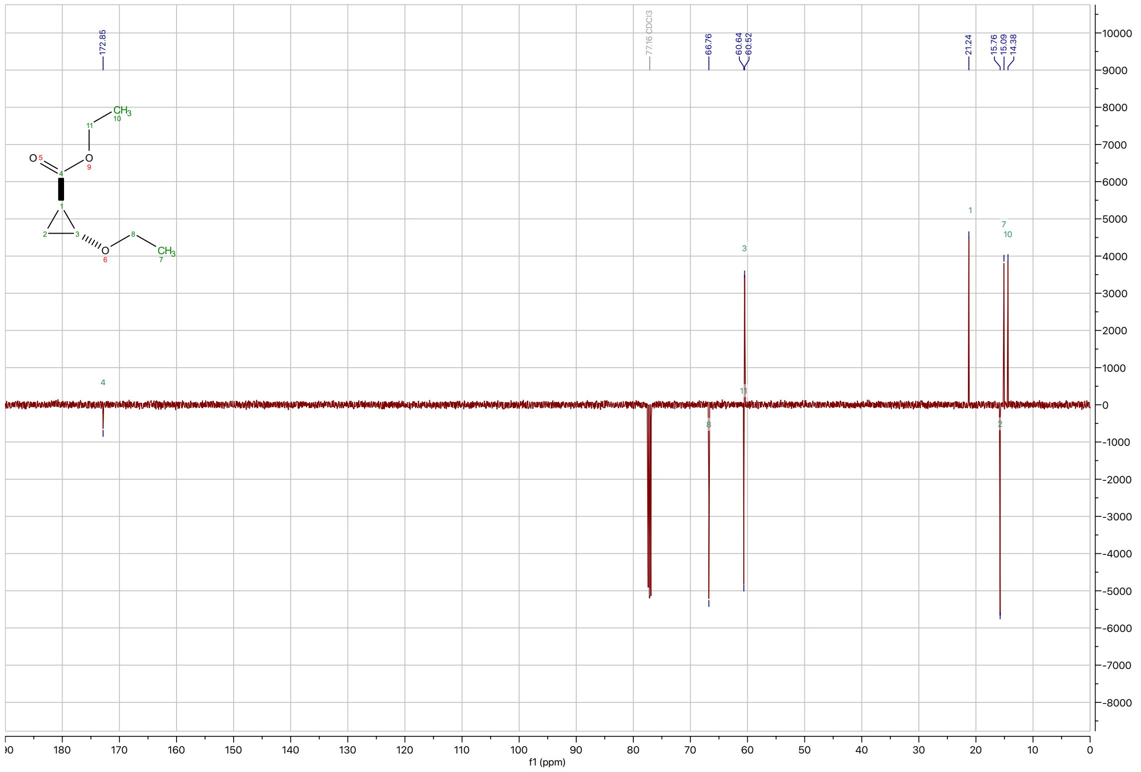

^{1}H-NMR spectrum of **2b**

^{1}H NMR (500 MHz, $CDCl_3$) δ 4.22 – 4.10 (m, 2H), 3.66 – 3.51 (m, 2H), 3.44 (dq, J = 9.5, 7.1 Hz, 1H), 1.69 (ddd, J = 8.6, 6.6, 6.6 Hz, 1H), 1.55 (ddd, J = 6.5, 6.4, 4.8 Hz, 1H), 1.26 (dd, J = 7.1, 7.1 Hz, 3H), 1.18 (dd, J = 7.1, 7.1 Hz, 3H), 1.06 (ddd, J = 8.6, 6.4, 6.4 Hz, 1H).

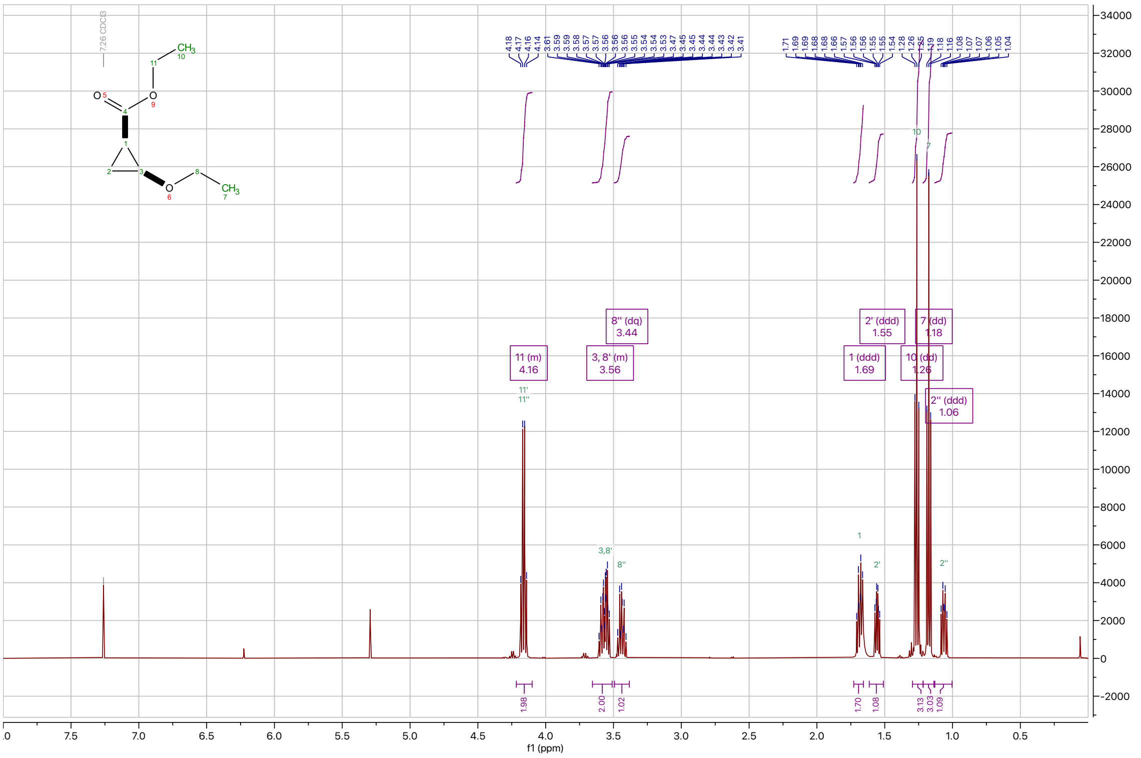

^{13}C-NMR spectrum of **2b**

^{13}C NMR (126 MHz, $CDCl_3$) δ 170.3, 66.9, 60.6, 59.0, 20.8, 15.0, 14.4, 13.2.

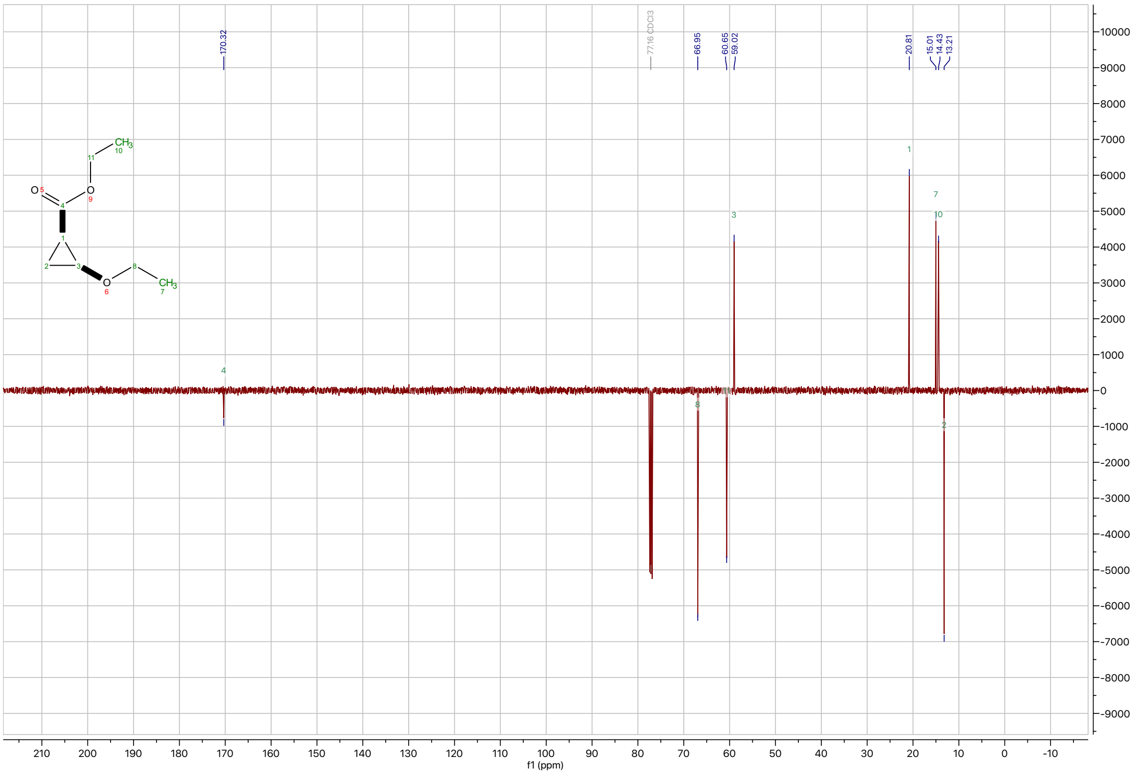

^{1}H-NMR spectrum of **3a**

^{1}H NMR (500 MHz, DMSO) δ 3.58 – 3.44 (m, 3H), 1.60 (ddd, *J* = 9.5, 5.9, 2.0 Hz, 1H), 1.14 (ddd, *J* = 9.5, 5.3, 4.2 Hz, 1H), 1.10 (dd, *J* = 7.0, 7.0 Hz, 3H), 1.05 (ddd, *J* = 6.7, 5.6, 5.6 Hz, 1H).

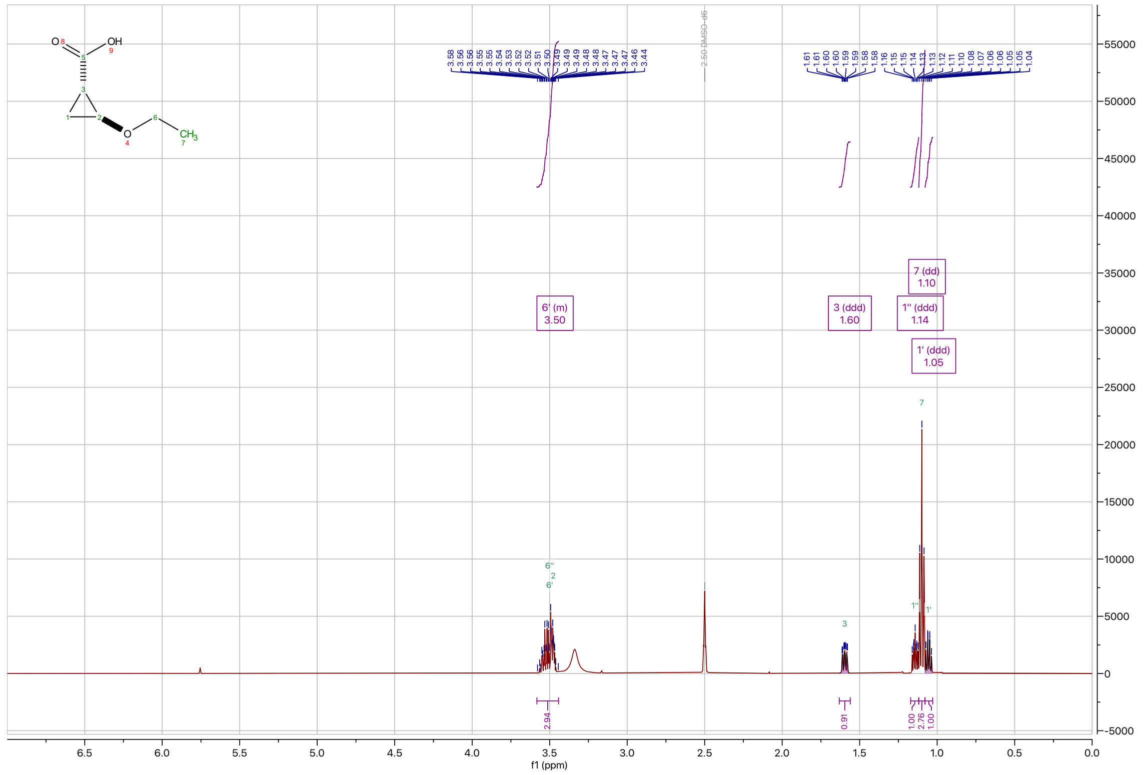

^{13}C-NMR spectrum of **3a**

^{13}C NMR (126 MHz, DMSO) δ 173.3, 65.7, 59.6, 20.4, 14.8, 14.7.

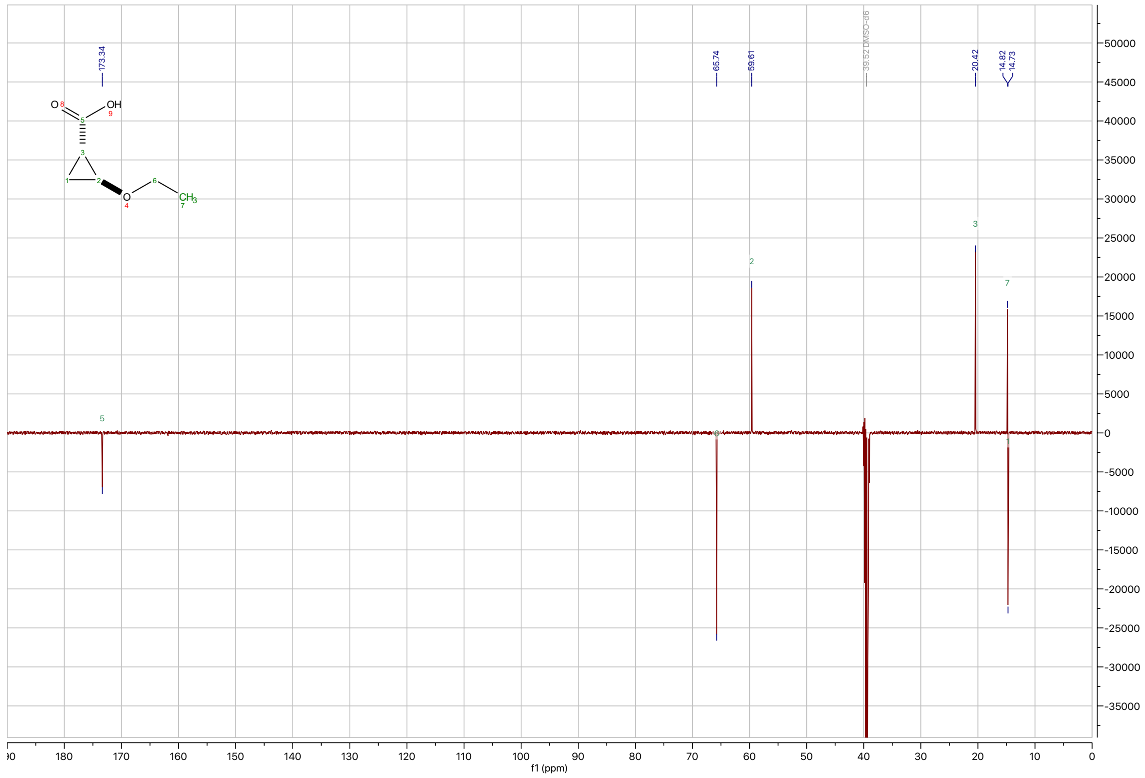

^{1}H-NMR spectrum of **3b**

^{1}H NMR (500 MHz, DMSO) δ 3.55 (ddd, J = 6.7, 6.7, 4.7 Hz, 1H), 3.49 (dq, J = 9.6, 7.0 Hz, 1H), 3.41 – 3.35 (m, 1H), 1.59 (ddd, J = 8.4, 6.7, 6.7 Hz, 1H), 1.21 (ddd, J = 6.6, 5.1, 5.1 Hz, 1H), 1.06 (dd, J = 7.0, 7.0 Hz, 3H), 0.97 (ddd, J = 8.4, 6.6, 5.5 Hz, 1H).

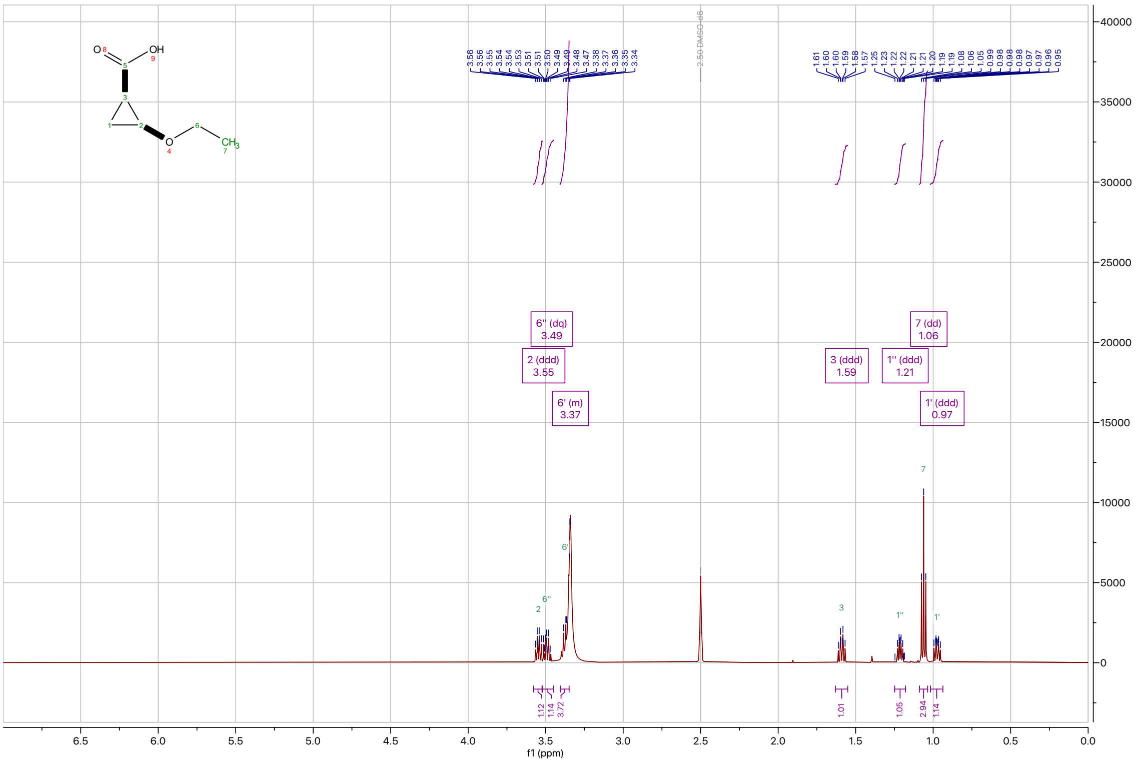

^{13}C-NMR spectrum of **3b**

^{13}C NMR (126 MHz, DMSO) δ 170.9, 65.7, 58.3, 20.1, 14.9, 12.3.

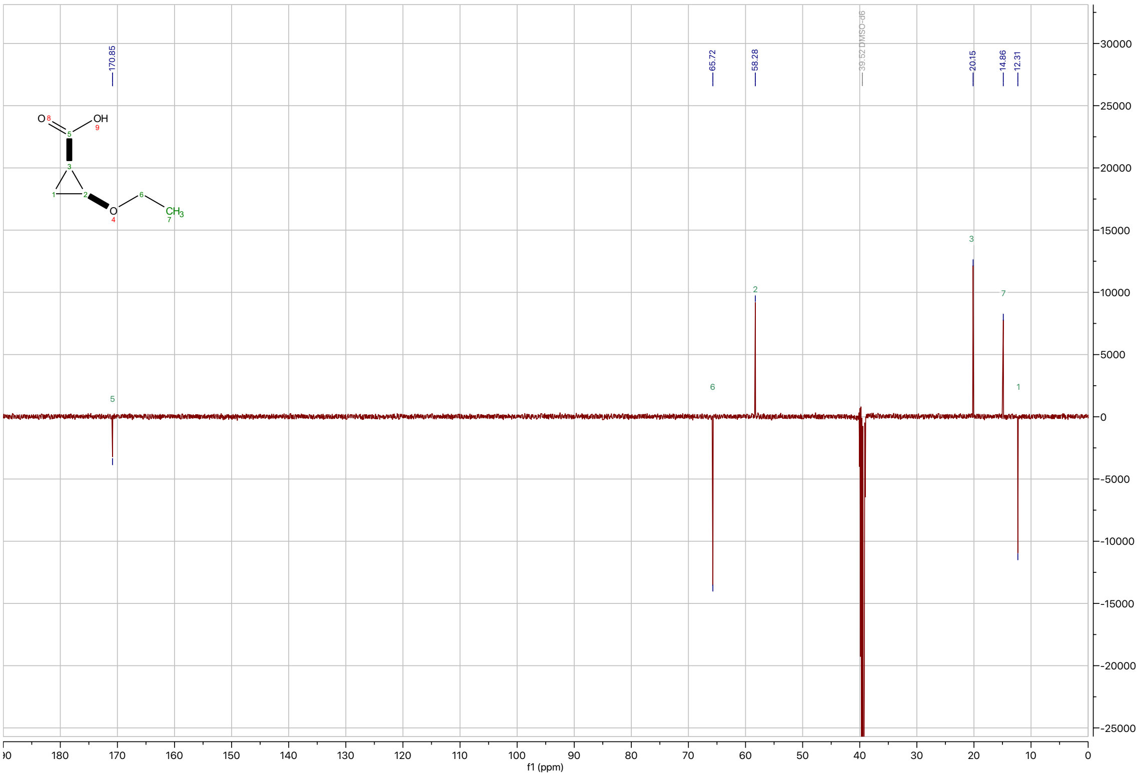

^{1}H-NMR spectrum of **S1**

^{1}H NMR (500 MHz, $CDCl_3$) δ 6.45 (dd, J = 15.0, 7.1 Hz, 1H), 5.99 (dd, J = 7.1, 1.9 Hz, 1H), 5.86 (dd, J = 15.0, 1.9 Hz, 1H)

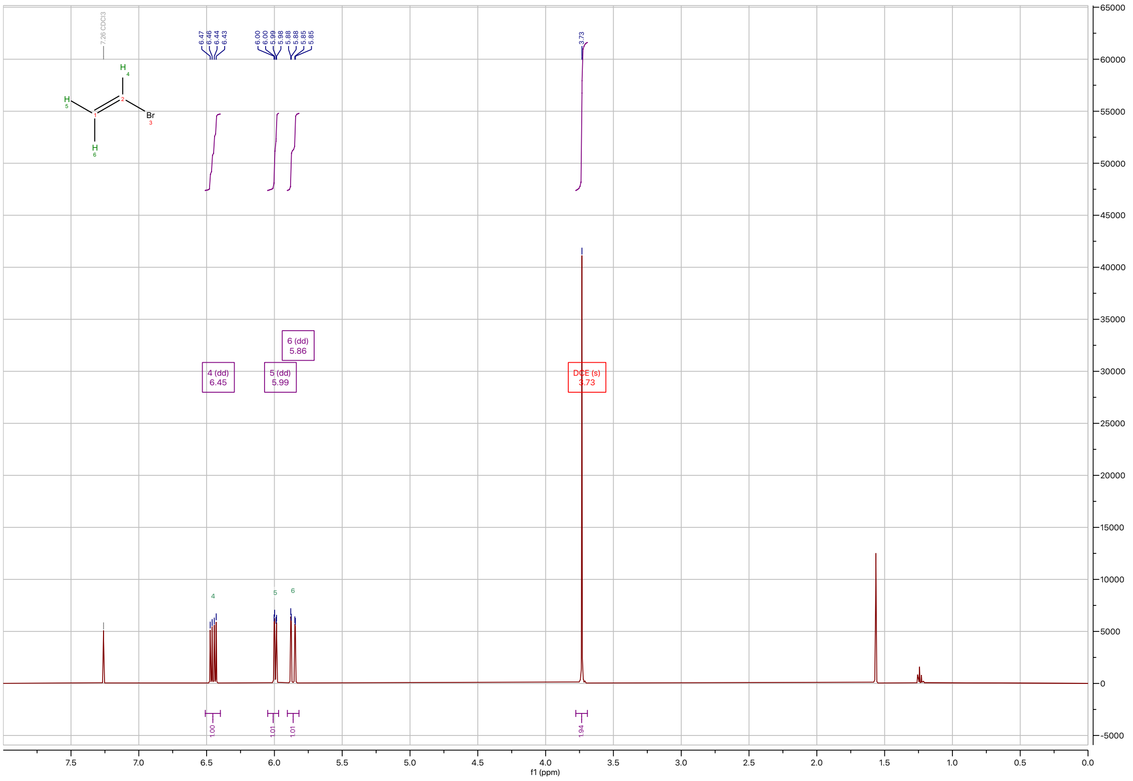

^{1}H-NMR spectrum of **5**

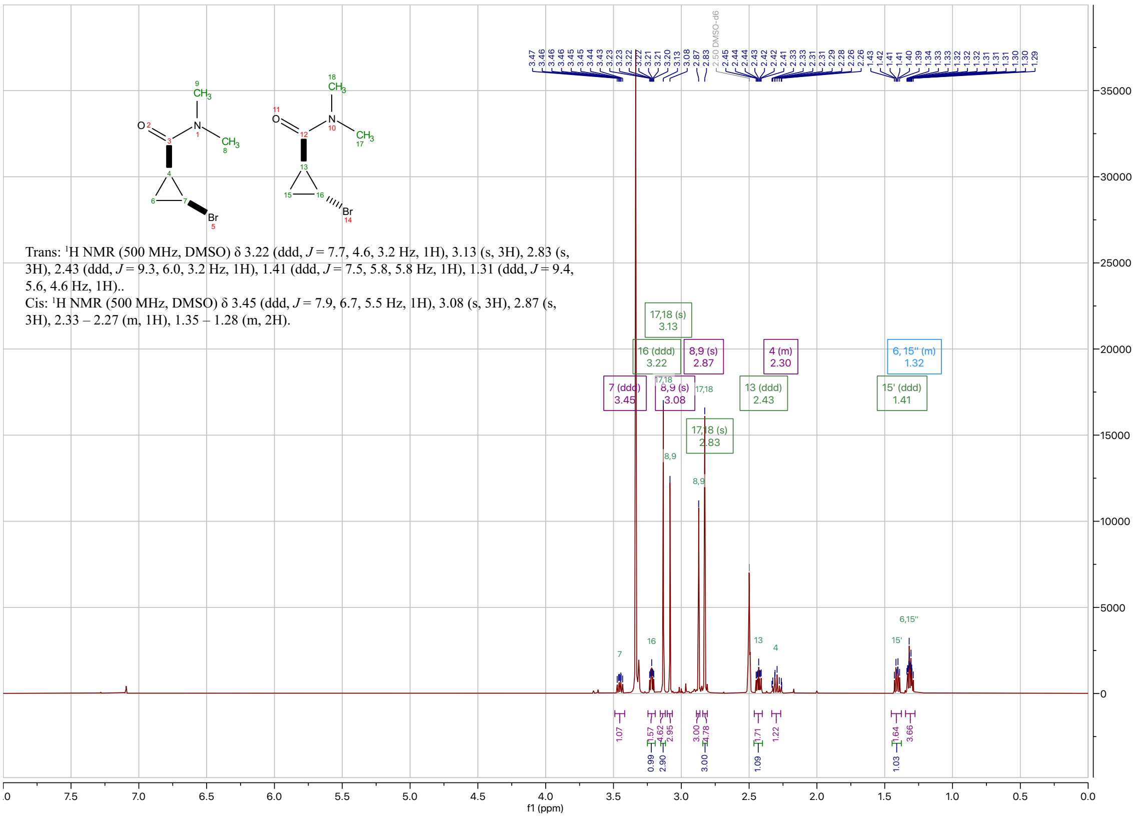

Trans: ^{1}H NMR (500 MHz, DMSO) δ 3.22 (ddd, J = 7.7, 4.6, 3.2 Hz, 1H), 3.13 (s, 3H), 2.83 (s, 3H), 2.43 (ddd, J = 9.3, 6.0, 3.2 Hz, 1H), 1.41 (ddd, J = 7.5, 5.8, 5.8 Hz, 1H), 1.31 (ddd, J = 9.4, 5.6, 4.6 Hz, 1H)..

Cis: ^{1}H NMR (500 MHz, DMSO) δ 3.45 (ddd, J = 7.9, 6.7, 5.5 Hz, 1H), 3.08 (s, 3H), 2.87 (s, 3H), 2.33 – 2.27 (m, 1H), 1.35 – 1.28 (m, 2H).

^{1}H-NMR spectrum of **6a**

^{1}H NMR (600 MHz, CD_3OD) δ 3.59 – 3.53 (m, 2H), 3.50 (ddd, J = 6.4, 4.0, 2.1 Hz, 1H), 2.63 (tt, J = 7.3, 3.9 Hz, 1H), 1.64 (ddd, J = 9.6, 5.9, 2.1 Hz, 1H), 1.19 – 1.12 (m, 4H), 1.06 (ddd, J = 9.4, 5.4, 4.0 Hz, 1H), 0.75 – 0.66 (m, 2H), 0.52 – 0.44 (m, 2H).

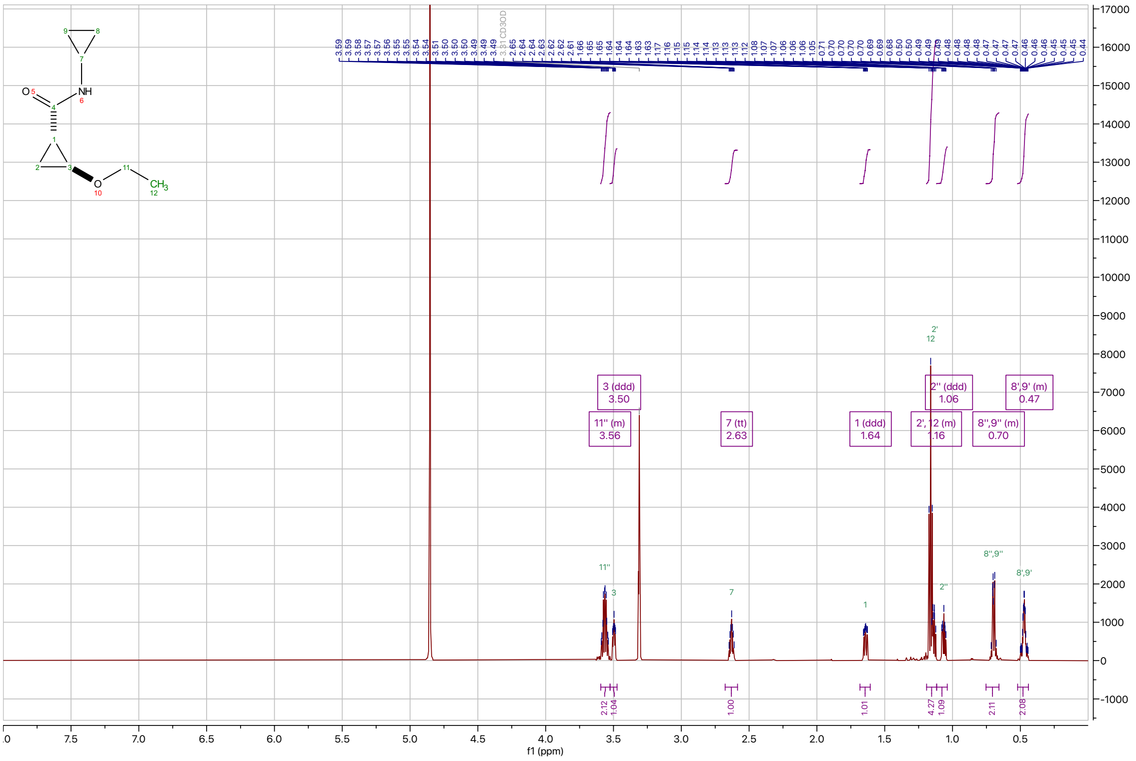

^{13}C-NMR spectrum of **6a**

^{13}C NMR (151 MHz, CD_3OD) δ 175.7, 67.3, 60.3, 23.4, 23.0, 15.3, 14.7, 6.5, 6.4.

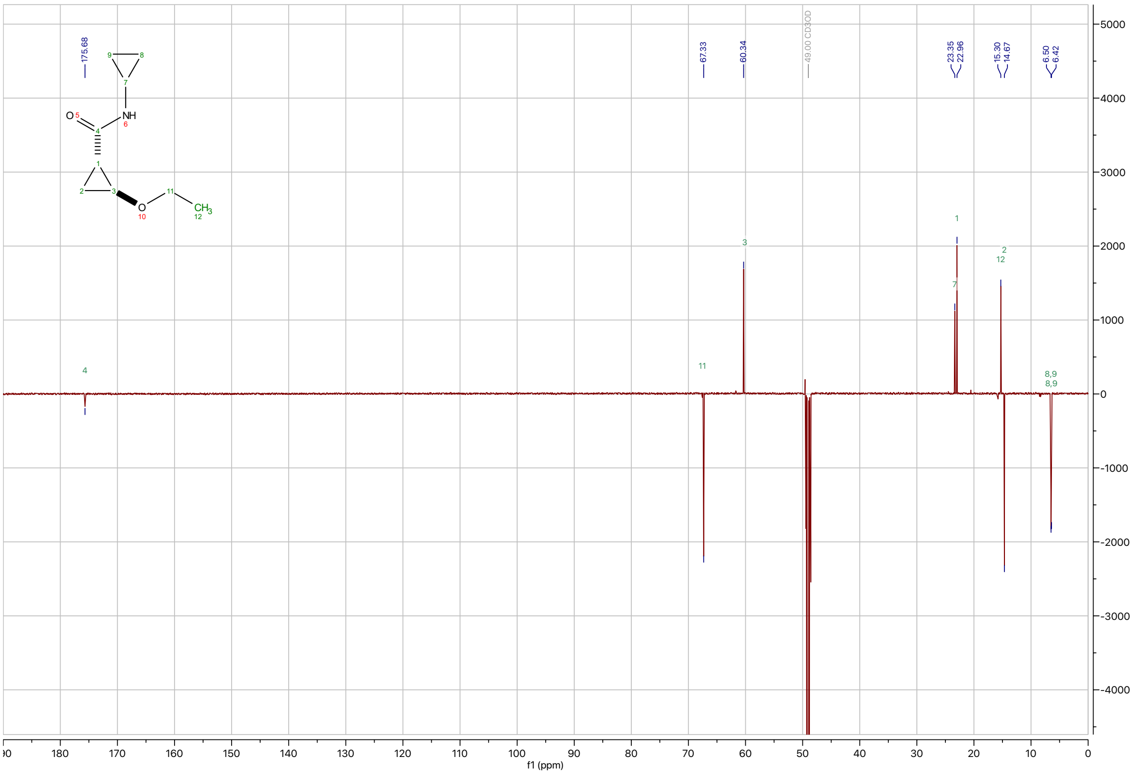

^{1}H-NMR spectrum of **6b**

^{1}H NMR (500 MHz, CD_3OD) δ 3.91 – 3.82 (m, 2H), 3.78 – 3.67 (m, 1H), 3.66 – 3.50 (m, 10H), 3.46 – 3.35 (m, 2H), 3.22 – 3.15 (m, 1H), 2.26 – 2.15 (m, 1H), 2.15 – 2.05 (m, 1H), 1.95 – 1.81 (m, 3H), 1.79 – 1.68 (m, 1H), 1.27 – 1.12 (m, 10H).

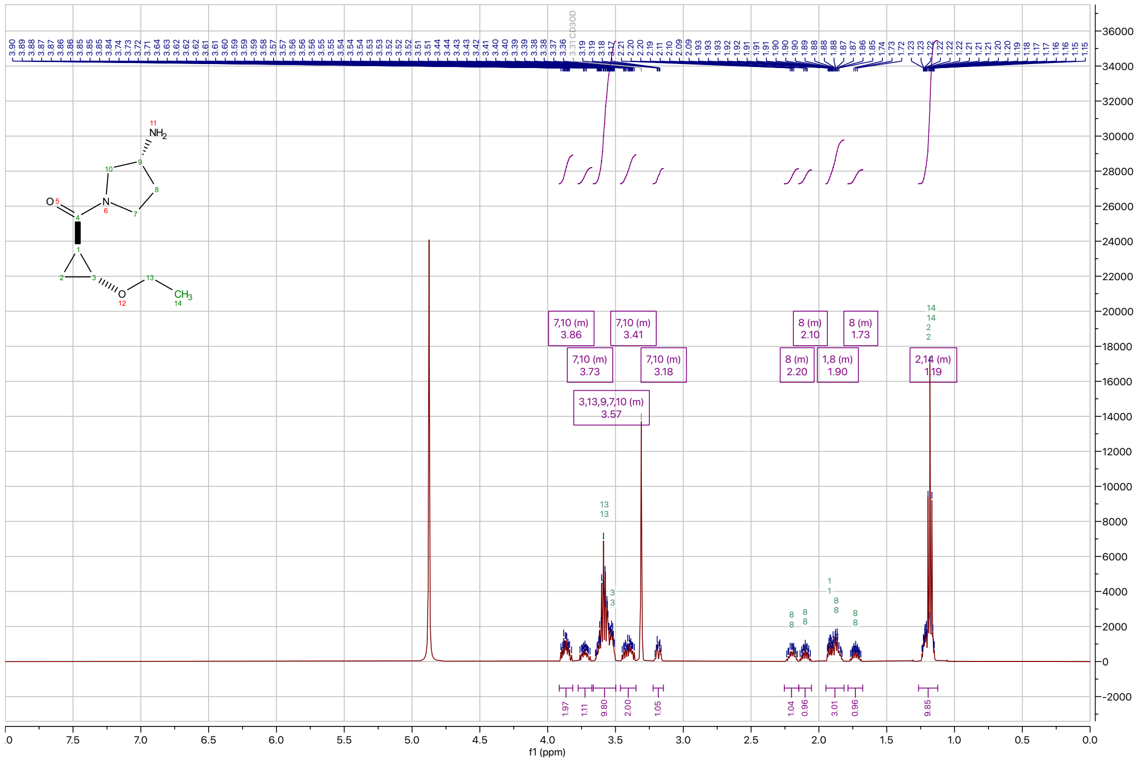

^{13}C-NMR spectrum of **6b**

^{13}C NMR (126 MHz, CD_3OD) δ 172.5, 172.3, 67.6, 67.6, 67.6, 61.3, 61.3, 61.2, 55.5, 55.4, 54.5, 52.4, 52.4, 50.8, 46.3, 45.5, 45.5, 34.8, 33.5, 21.8, 21.8, 21.5, 21.5, 15.5, 15.4, 15.4, 15.4, 15.3.

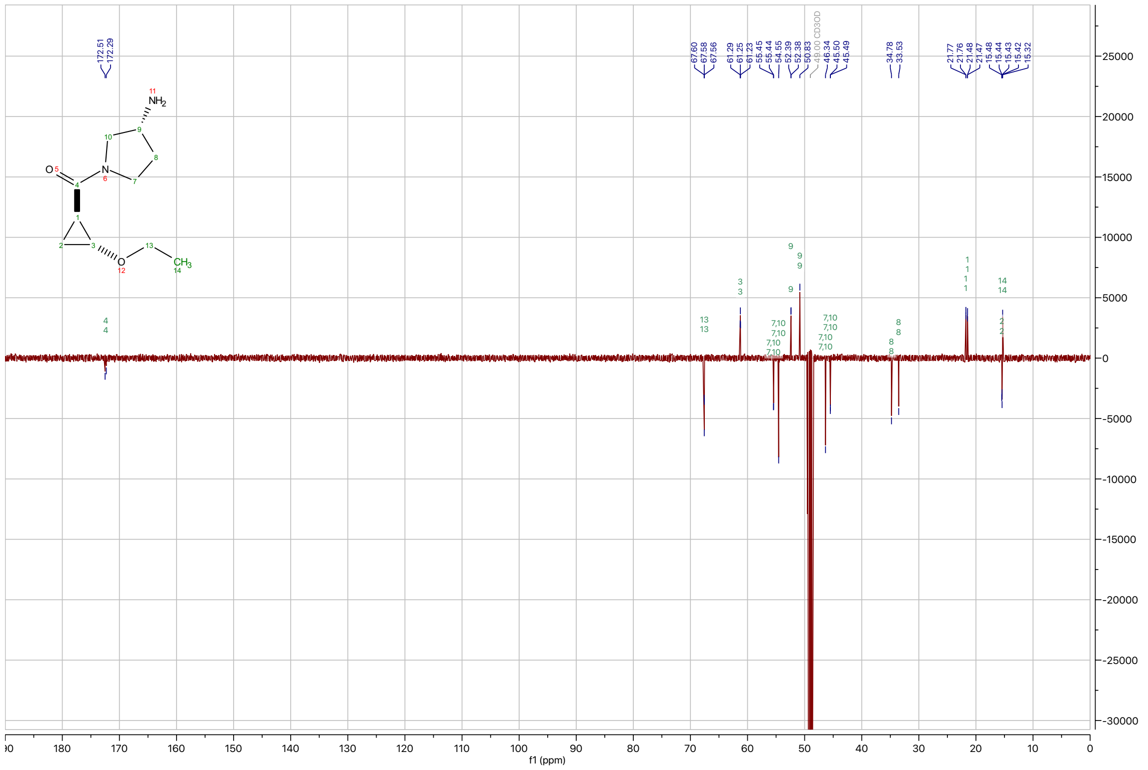

^{1}H-NMR spectrum of **6c**

^{1}H NMR (600 MHz, CD_3OD) δ 4.10 – 4.00 (m, 1H), 4.00 – 3.90 (m, 1H), 3.63 – 3.54 (m, 3H), 3.50 (ddd, J = 6.4, 4.0, 2.1 Hz, 1H), 3.24 – 3.14 (m, 1H), 2.12 (ddd, J = 9.5, 5.9, 2.1 Hz, 1H), 1.71 – 1.64 (m, 1H), 1.64 – 1.54 (m, 2H), 1.53 – 1.44 (m, 1H), 1.28 – 1.22 (m, 3H), 1.21 – 1.16 (m, 4H), 1.16 – 1.11 (m, 1H).

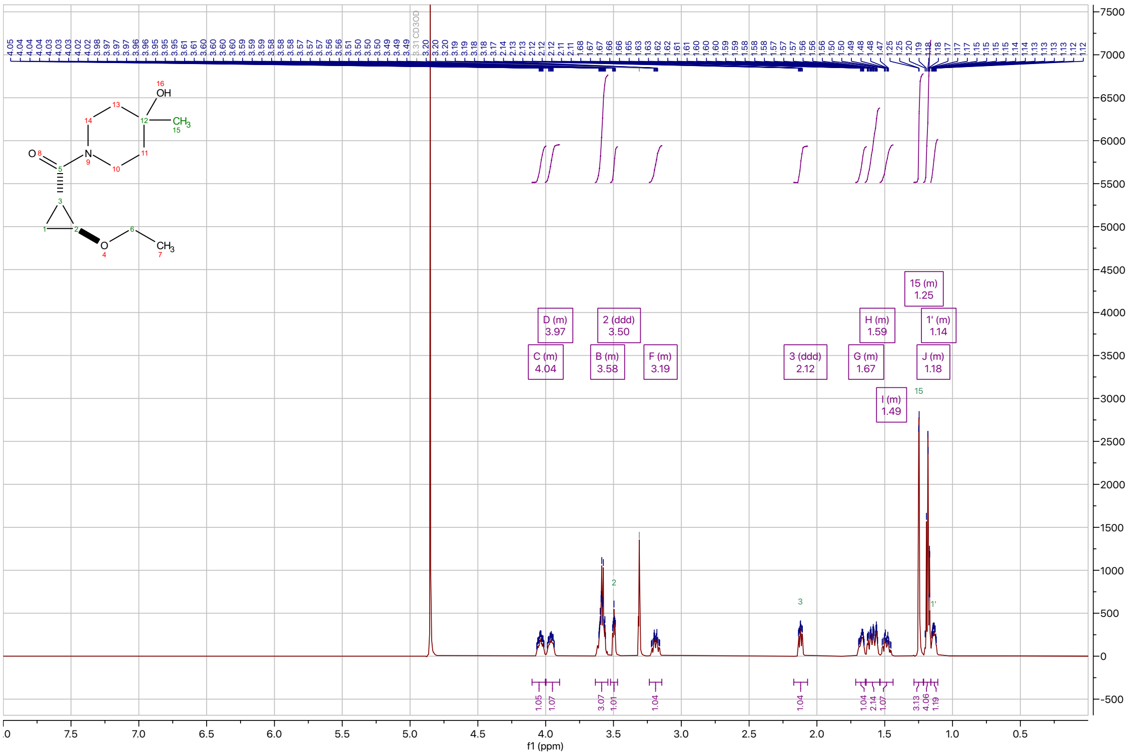

^{13}C-NMR spectrum of **6c**

^{13}C NMR (151 MHz, CD_3OD) δ 171.9, 171.8, 68.4, 67.5, 67.4, 61.2, 43.43, 43.40, 40.05, 39.98, 39.91, 39.89, 39.0, 29.92, 29.87, 20.1, 15.36, 15.34, 15.32.

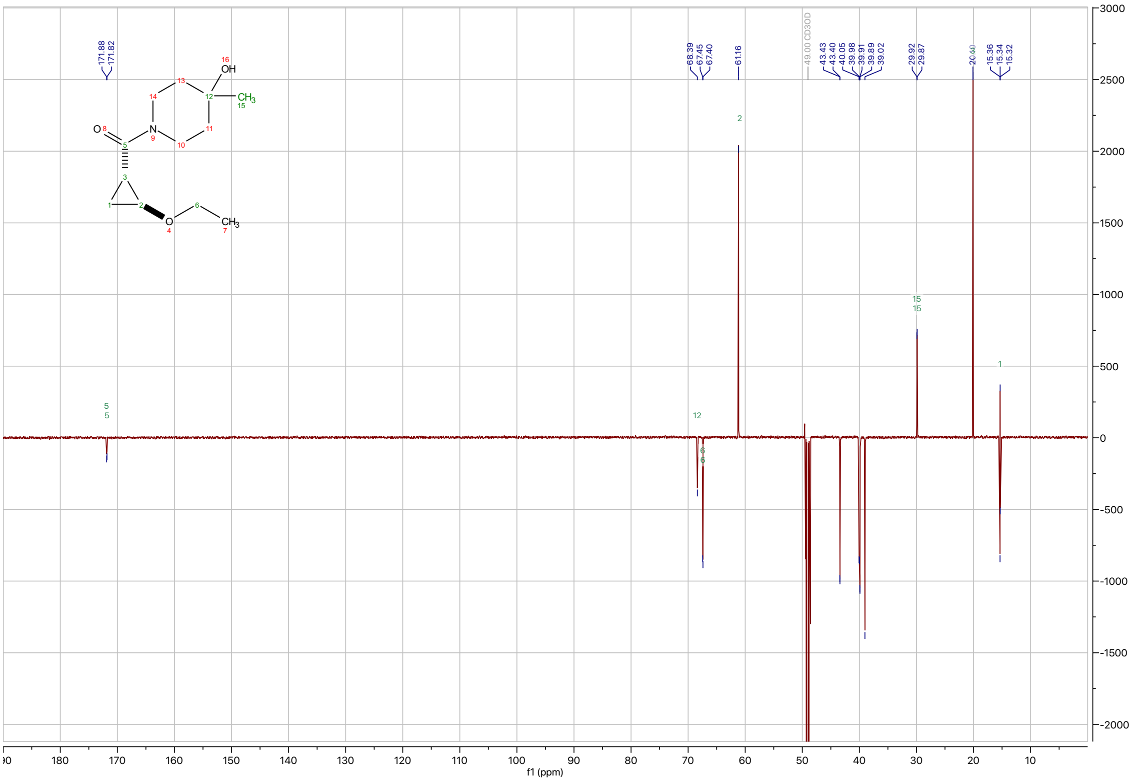

^{1}H-NMR spectrum of **6d**

^{1}H NMR (600 MHz, CD_3OD) δ 3.56 (dq, J = 9.4, 7.0 Hz, 1H), 3.51 (ddd, J = 6.6, 6.6, 4.5 Hz, 1H), 3.46 (dq, J = 9.4, 7.0 Hz, 1H), 2.64 (tt, J = 7.3, 3.9 Hz, 1H), 1.57 (ddd, J = 9.0, 6.6 Hz, 1H), 1.38 (ddd, J = 6.8, 6.0, 4.5 Hz, 1H), 1.15 (dd, J = 7.1, 7.1 Hz, 3H), 0.98 (ddd, J = 9.0, 6.6, 6.0 Hz, 1H), 0.74 – 0.66 (m, 2H), 0.50 – 0.42 (m, 2H).

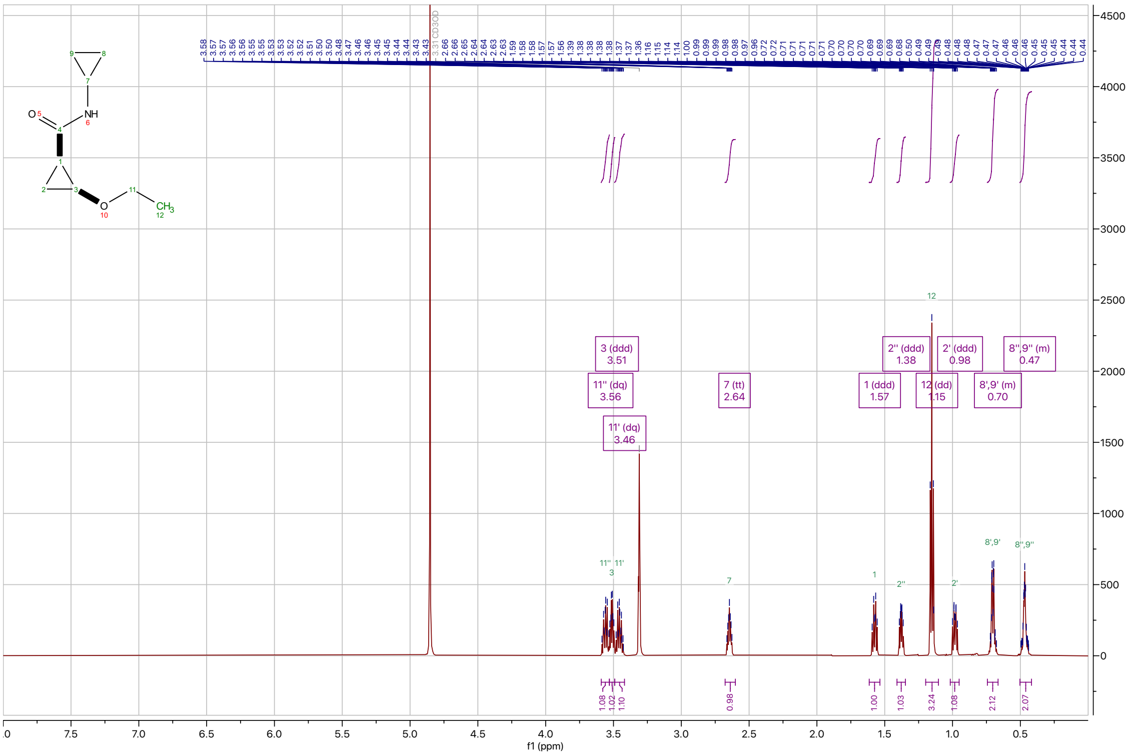

^{13}C-NMR spectrum of **6d**

^{13}C NMR (151 MHz, CD_3OD) δ 173.8, 67.6, 59.1, 23.5, 22.6, 15.2, 12.2, 6.51, 6.50.

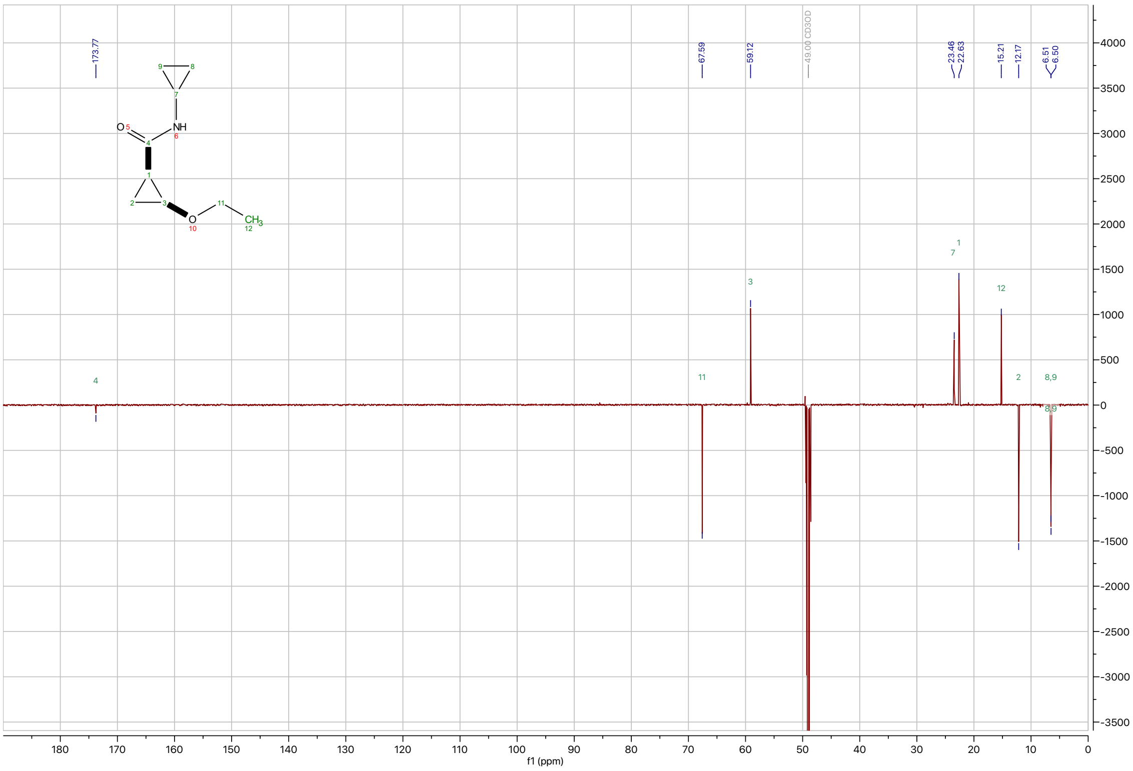

^{1}H-NMR spectrum of **6e**

^{1}H NMR (500 MHz, CD_3OD) δ 3.86 – 3.65 (m, 3H), 3.61 (ddd, J = 6.5, 6.5, 4.1 Hz, 1H), 3.58 – 3.43 (m, 3H), 2.98 – 2.73 (m, 4H), 1.95 (ddd, J = 8.8, 6.8, 6.8 Hz, 1H), 1.37 (ddd, J = 6.3, 6.2, 4.1 Hz, 1H), 1.14 (dd, J = 7.1, 7.1 Hz, 3H), 0.92 (ddd, J = 8.8, 6.0, 6.0 Hz, 1H).

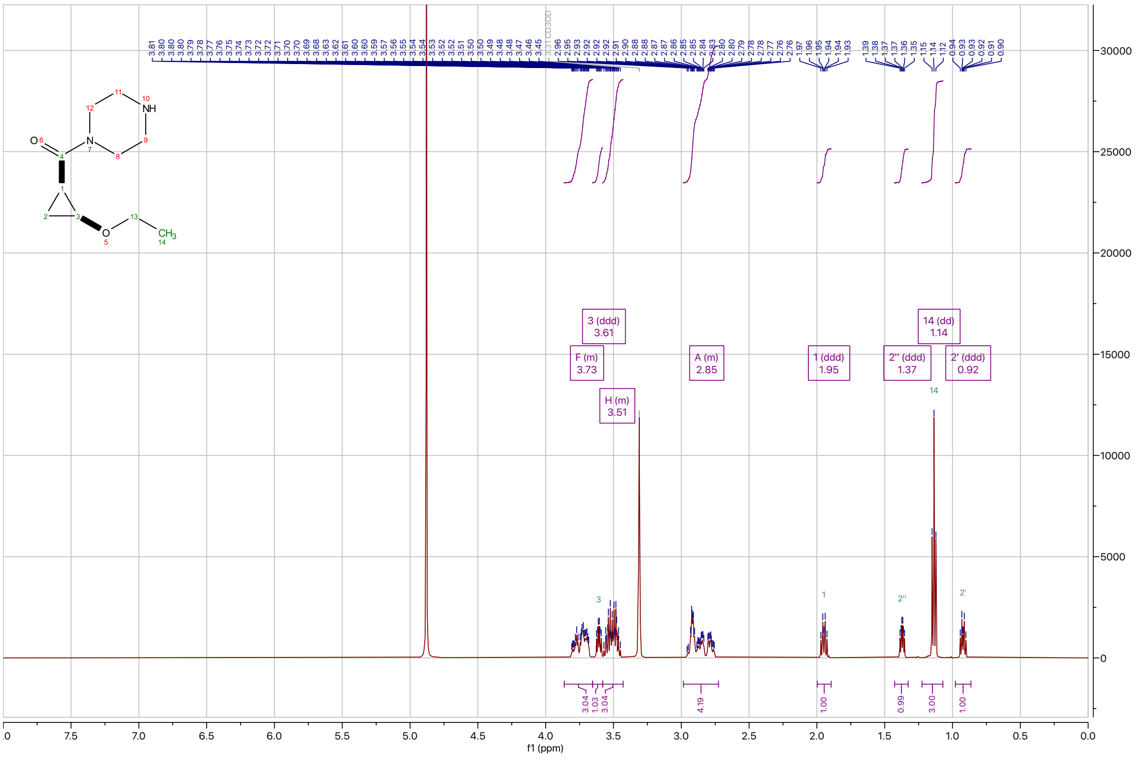

^{13}C-NMR spectrum of **6e**

^{13}C NMR (126 MHz, CD_3OD) δ 169.7, 67.4, 58.4, 47.1, 46.6, 46.1, 43.6, 21.6, 15.3, 11.4.

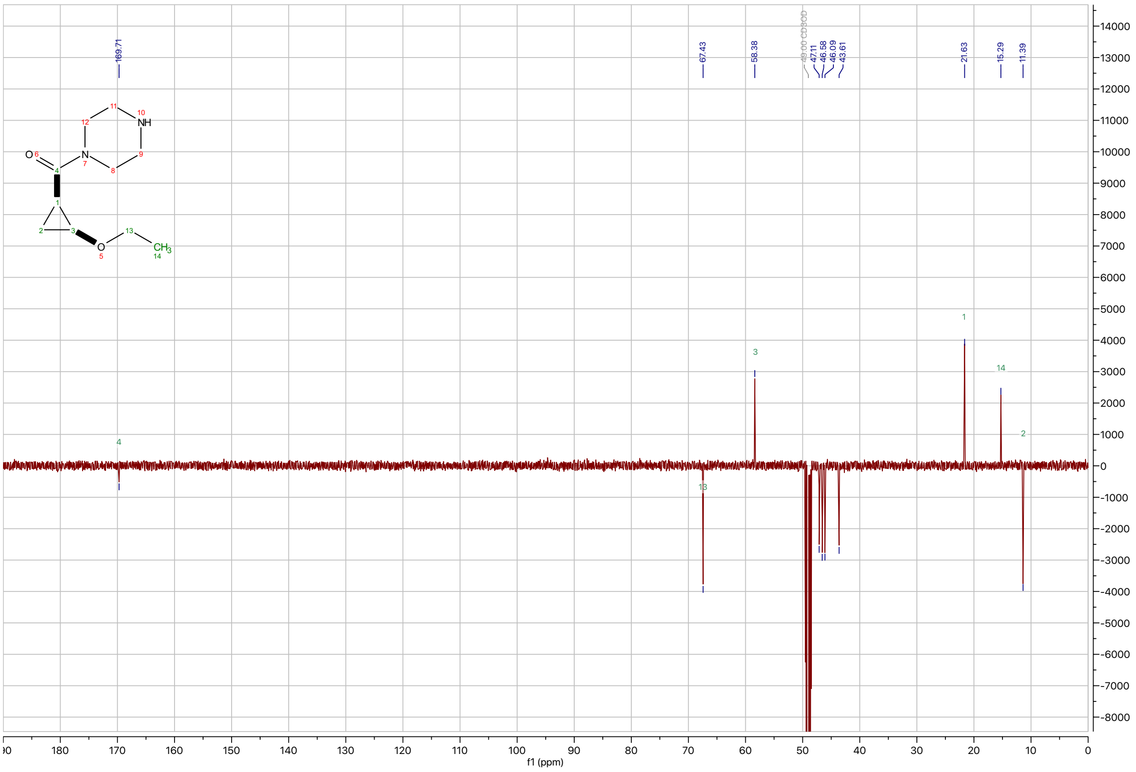

^{1}H-NMR spectrum of **6f**

^{1}H NMR (600 MHz, $CDCl_3$) δ 8.37 (d$^{\Delta}$, J = 8.3 Hz, 1H), 8.22 (dd, J = 4.8, 1.7 Hz, 1H), 8.21 (s, 1H), 7.14 (dd$^{\Delta}$, J = 8.2, 4.8 Hz, 1H), 3.83 – 3.71 (m, 2H), 3.65 (ddd, J = 6.2, 6.2, 4.4 Hz, 1H), 2.46 (s, 3H), 1.76 (ddd, J = 10.5, 6.7, 6.7 Hz, 1H), 1.38 – 1.27 (m, 4H), 1.21 (ddd, J = 6.8, 6.7, 4.5 Hz, 1H).

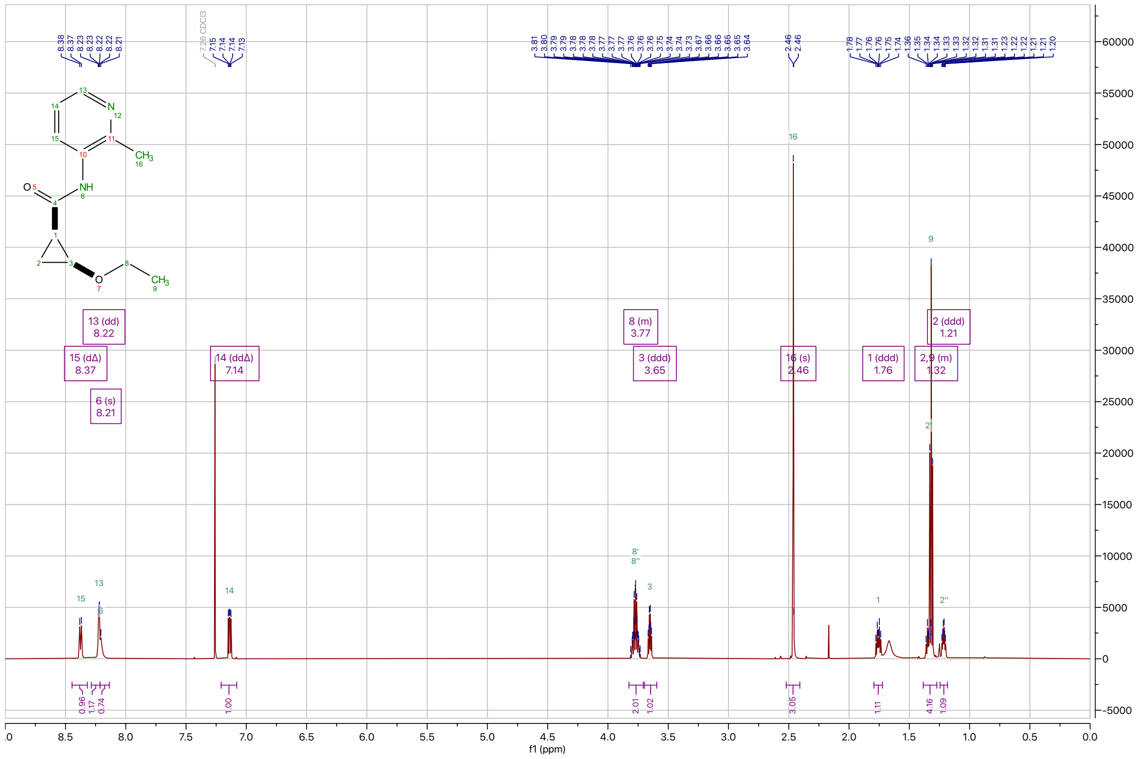

^{13}C-NMR spectrum of **6f**

^{13}C NMR (151 MHz, $CDCl_3$) δ 170.8, 148.0, 144.4, 133.0, 128.9, 121.9, 67.8, 58.0, 22.7, 21.1, 15.14, 15.13.

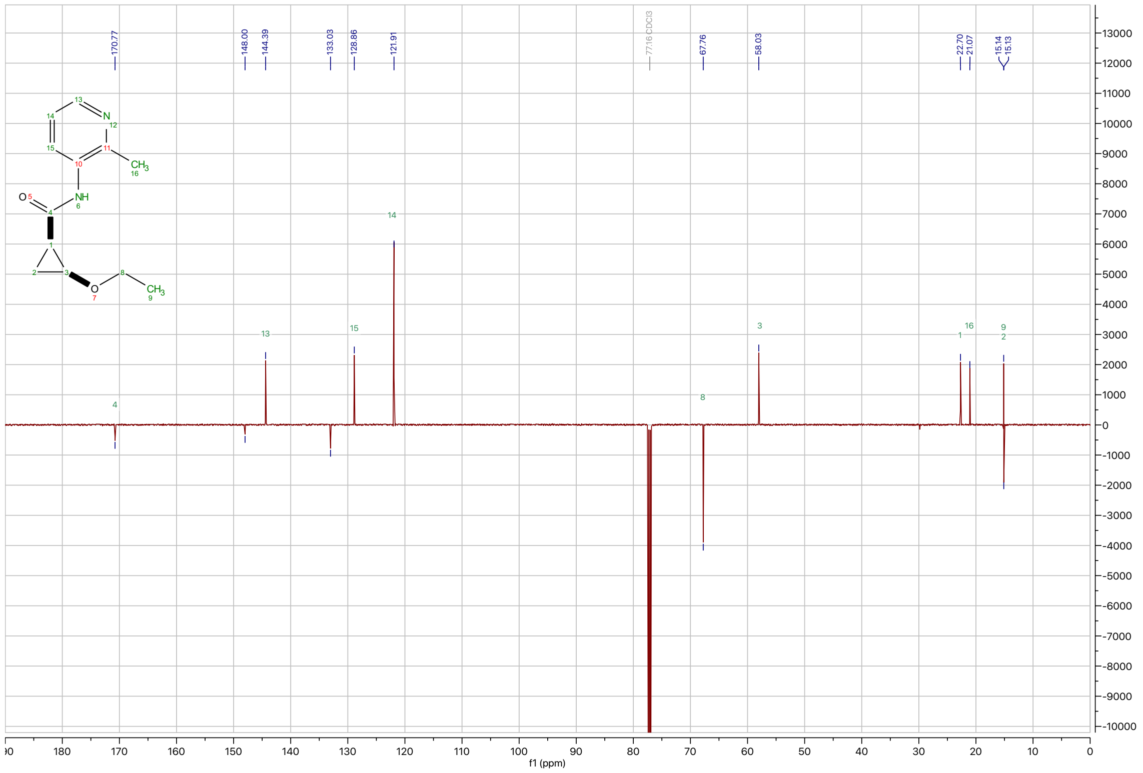

^{1}H-NMR spectrum of **7a**

^{1}H NMR (500 MHz, $CDCl_3$) δ 7.12 (dd, *J* = 7.8, 1.8 Hz, 1H), 7.00 – 6.87 (m, 3H), 4.14 (ddd, *J* = 6.3, 3.9, 2.2 Hz, 1H), 3.86 (s, 3H), 3.14 (s, 3H), 3.00 (s, 3H), 2.14 (ddd, *J* = 9.7, 6.1, 2.2 Hz, 1H), 1.55 – 1.49 (m, 1H), 1.45 (ddd, *J* = 9.6, 5.6, 3.9 Hz, 1H).

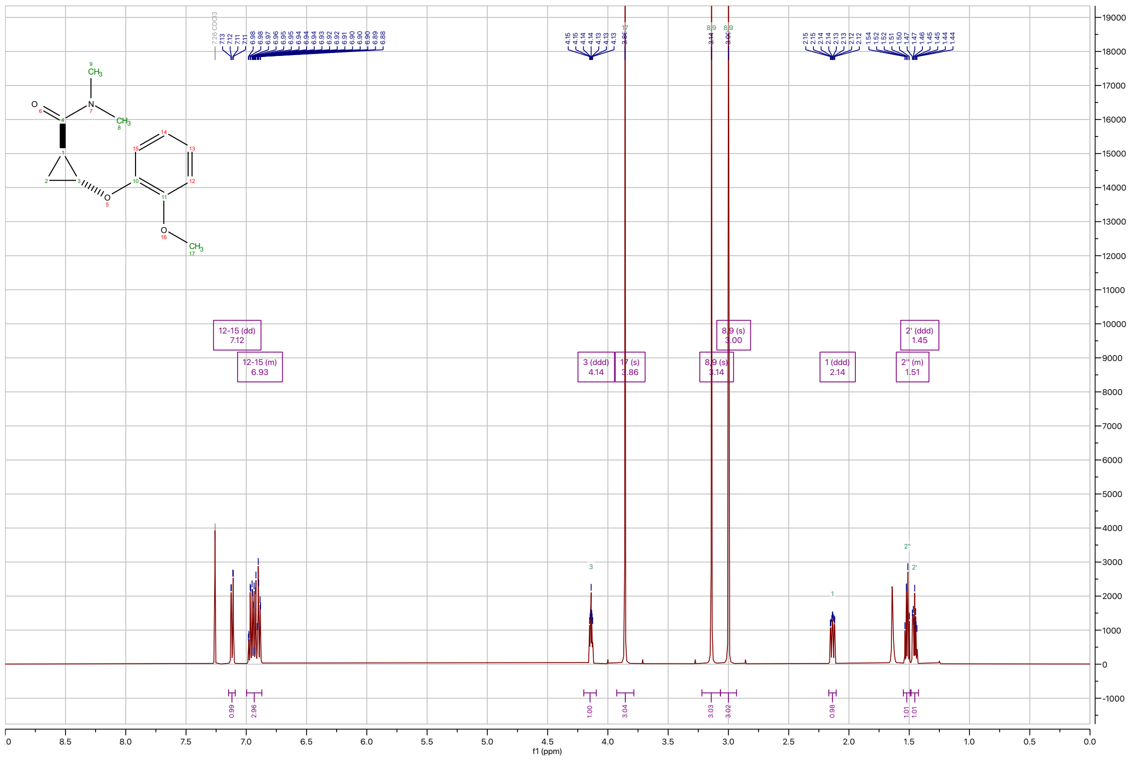

^{13}C-NMR spectrum of **7a**

^{13}C NMR (126 MHz, $CDCl_3$) δ 170.9, 149.0, 147.8, 121.9, 121.0, 113.8, 111.7, 58.1, 56.0, 37.5, 35.9, 20.0, 15.8.

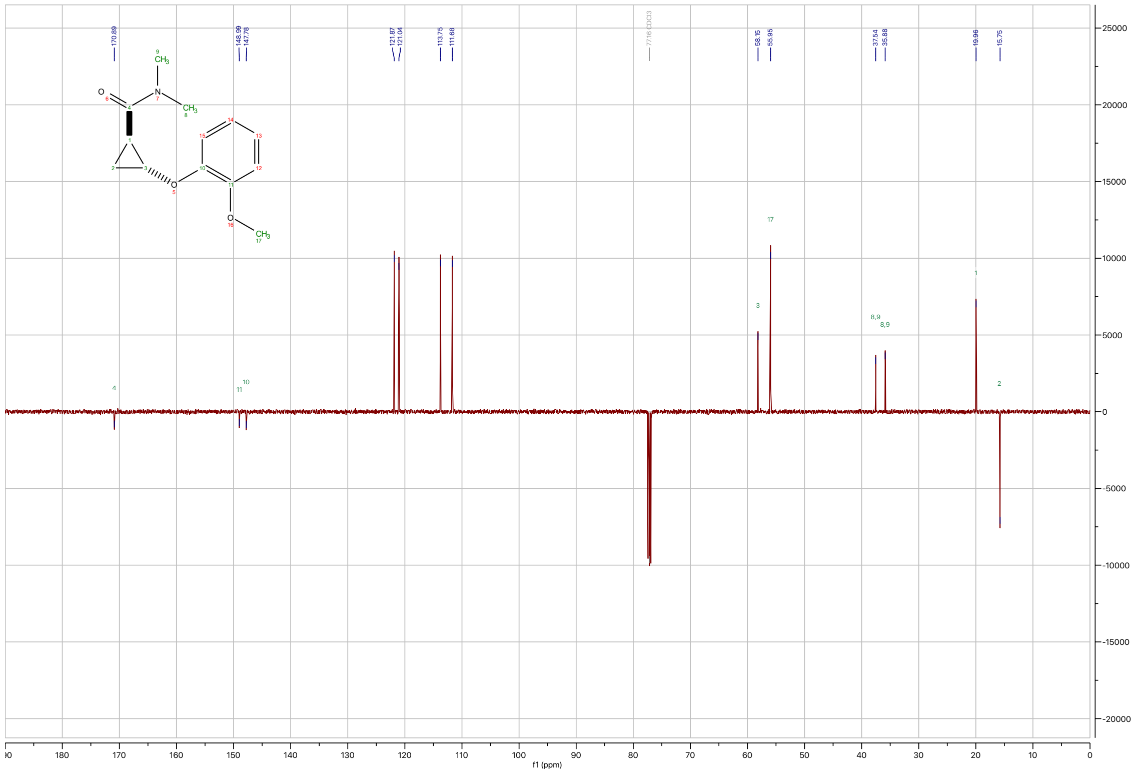

^{1}H-NMR spectrum of **7a’**

^{1}H NMR (600 MHz, $CDCl_3$) δ 7.07 – 7.03 (m, 1H), 6.95 (ddd, *J* = 7.9, 7.8, 1.7 Hz, 1H), 6.89 – 6.84 (m, 2H), 4.19 (ddd, *J* = 6.6, 6.6, 4.1 Hz, 1H), 3.81 (s, 3H), 3.09 (s, 3H), 2.92 (s, 3H), 2.00 (ddd, *J* = 9.0, 6.8, 6.8 Hz, 1H), 1.80 (ddd, *J* = 6.6, 6.5, 4.1 Hz, 1H), 1.13 (ddd, *J* = 9.0, 6.3, 6.3 Hz, 1H).

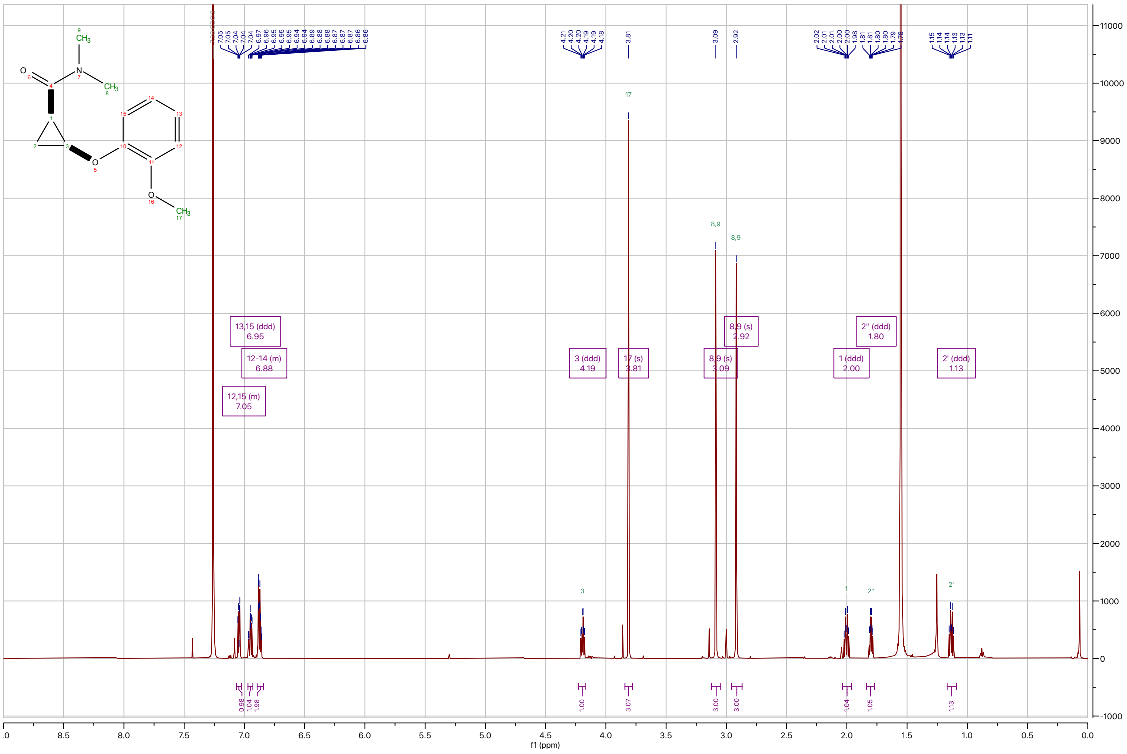

^{13}C-NMR spectrum of **7a’**

^{13}C NMR (151 MHz, $CDCl_3$) δ 122.6, 120.9, 116.5, 113.3, 56.6, 56.3, 37.3, 35.9, 21.4, 11.7. (three missing signals)

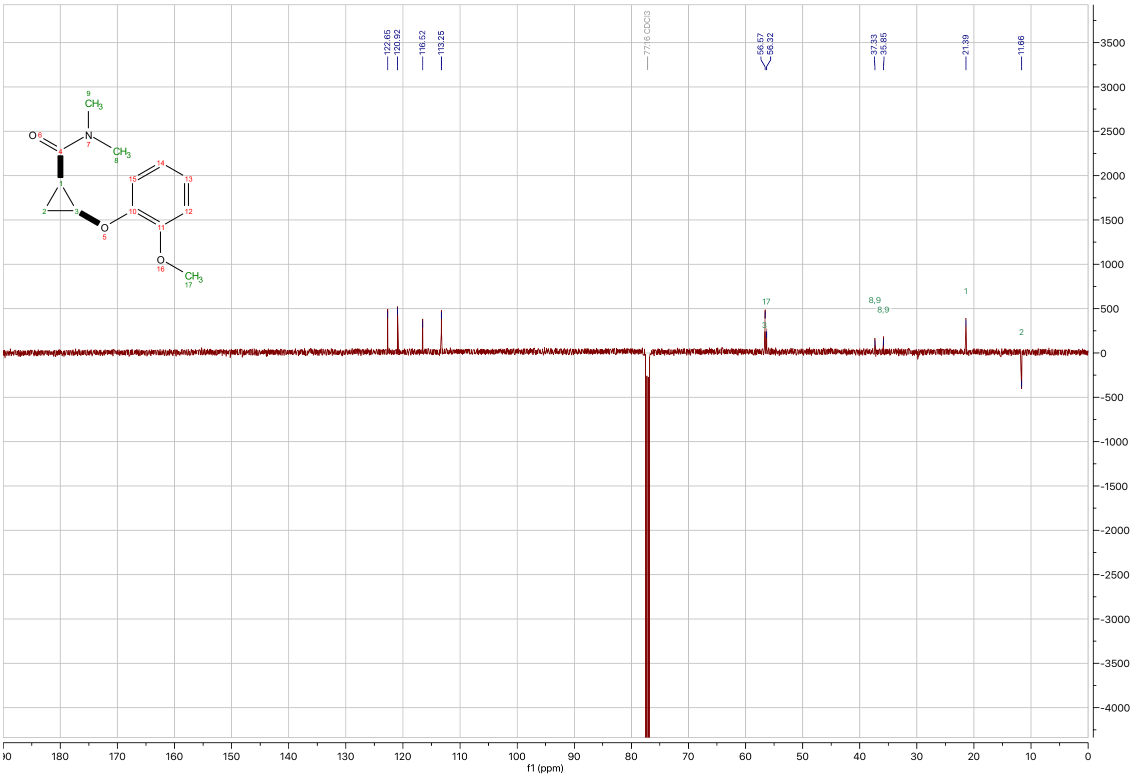

^{1}H-NMR spectrum of **7b**

^{1}H NMR (500 MHz, $CDCl_3$) δ 7.36 – 7.27 (m, 2H), 7.07 – 6.98 (m, 2H), 4.57 (d, *J* = 11.5 Hz, 1H), 4.49 (d, *J* = 11.6 Hz, 1H), 3.67 (ddd, *J* = 6.3, 3.9, 2.1 Hz, 1H), 3.08 (s, 3H), 2.94 (s, 3H), 1.91 (ddd, *J* = 9.5, 5.8, 2.1 Hz, 1H), 1.32 – 1.25 (m, 1H), 1.17 (ddd, *J* = 9.3, 5.2, 3.9 Hz, 1H).

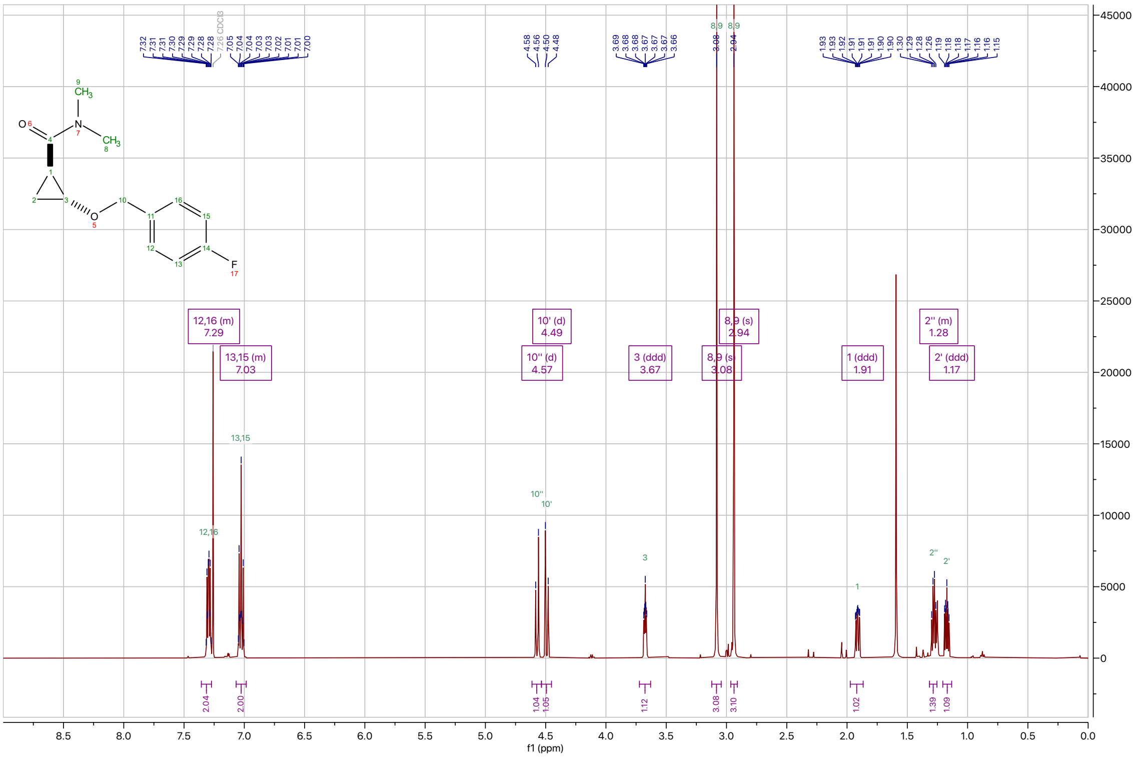

^{13}C-NMR spectrum of **7b**

^{13}C NMR (126 MHz, $CDCl_3$) δ 171.4, 162.6 (d, *J* = 246.1 Hz), 133.5 (d, *J* = 3.2 Hz), 130.0 (d, *J* = 8.2 Hz), 115.5 (d, *J* = 21.5 Hz), 72.7, 60.7, 37.4, 35.8, 19.7, 15.3.

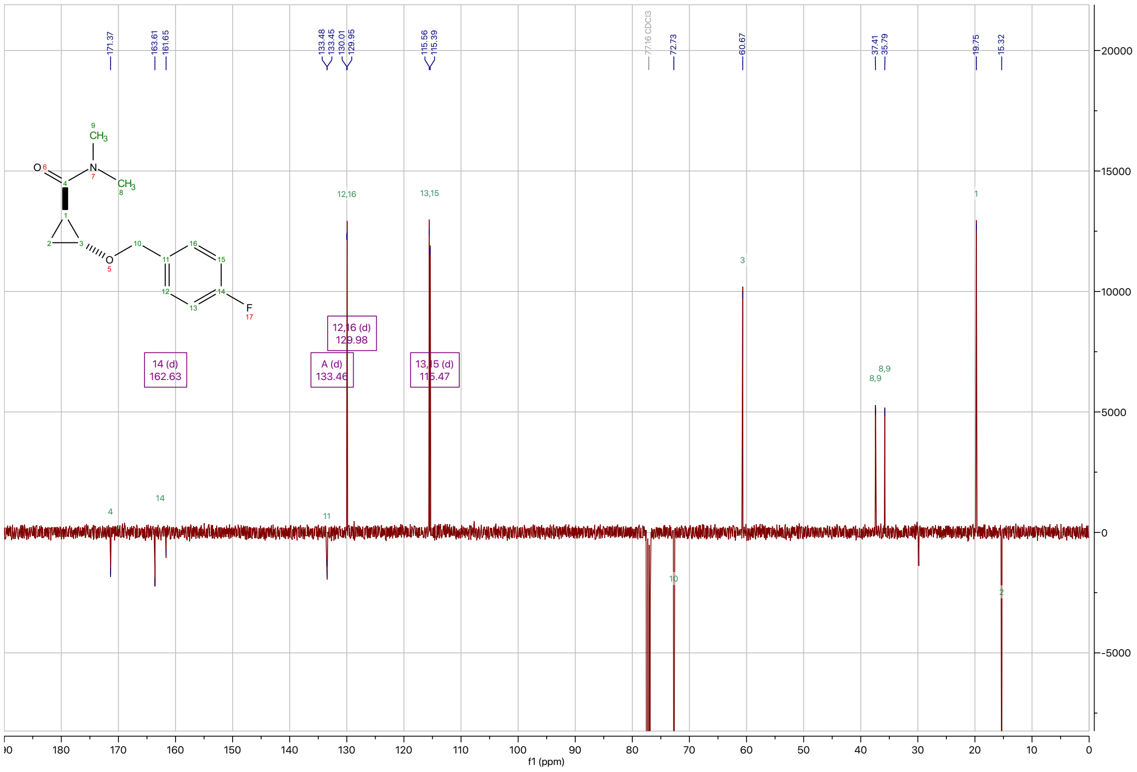

^{1}H-NMR spectrum of **7b'**

^{1}H NMR (600 MHz, $CDCl_3$) δ 7.33 – 7.27 (m, 2H), 7.05 – 6.97 (m, 2H), 4.52 (d, J = 11.7 Hz, 1H), 4.41 (d, J = 11.7 Hz, 1H), 3.58 (ddd, J = 6.6, 6.6, 4.2 Hz, 1H), 3.14 (s, 3H), 2.99 (s, 3H), 1.82 (ddd, J = 8.9, 6.7, 6.7 Hz, 1H), 1.66 – 1.63 (m, 1H), 0.91 (ddd, J = 8.9, 6.1, 6.1 Hz, 1H).

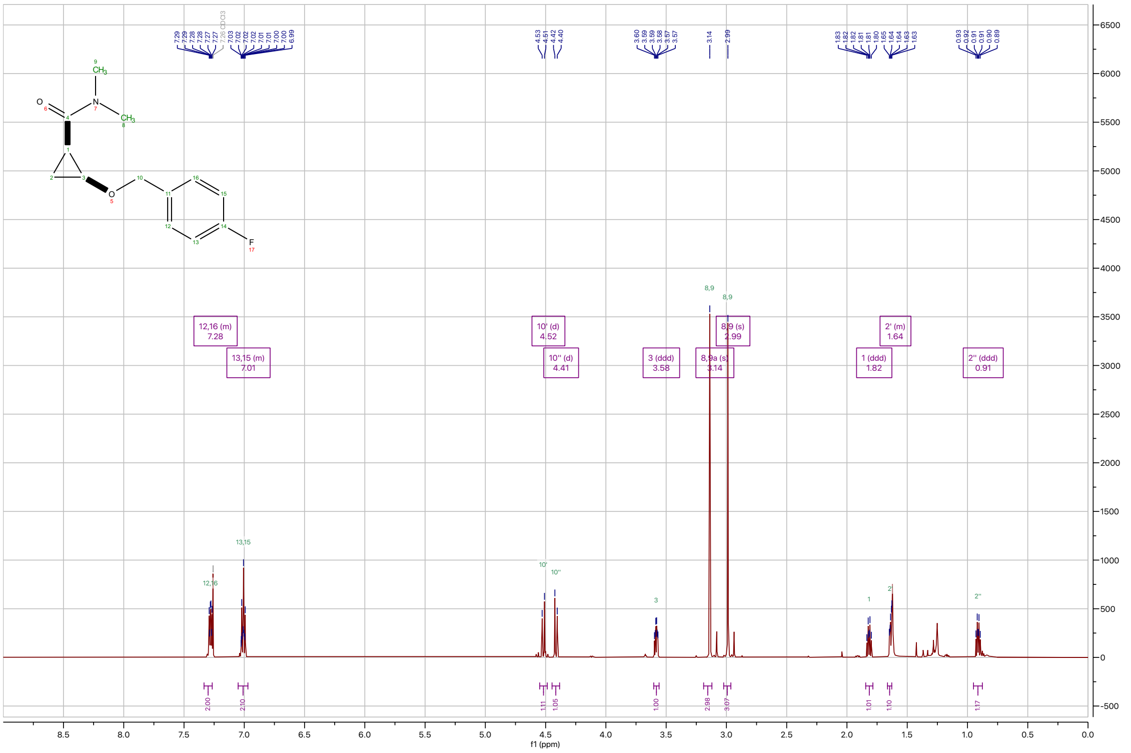

^{13}C-NMR spectrum of **7b'**

^{13}C NMR (151 MHz, $CDCl_3$) δ 168.3, 162.5 (d, J = 245.8 Hz), 133.6 (d, J = 3.2 Hz), 129.9 (d, J = 8.2 Hz), 115.3 (d, J = 21.3 Hz), 72.5, 57.3, 37.3, 35.9, 21.4, 11.2.

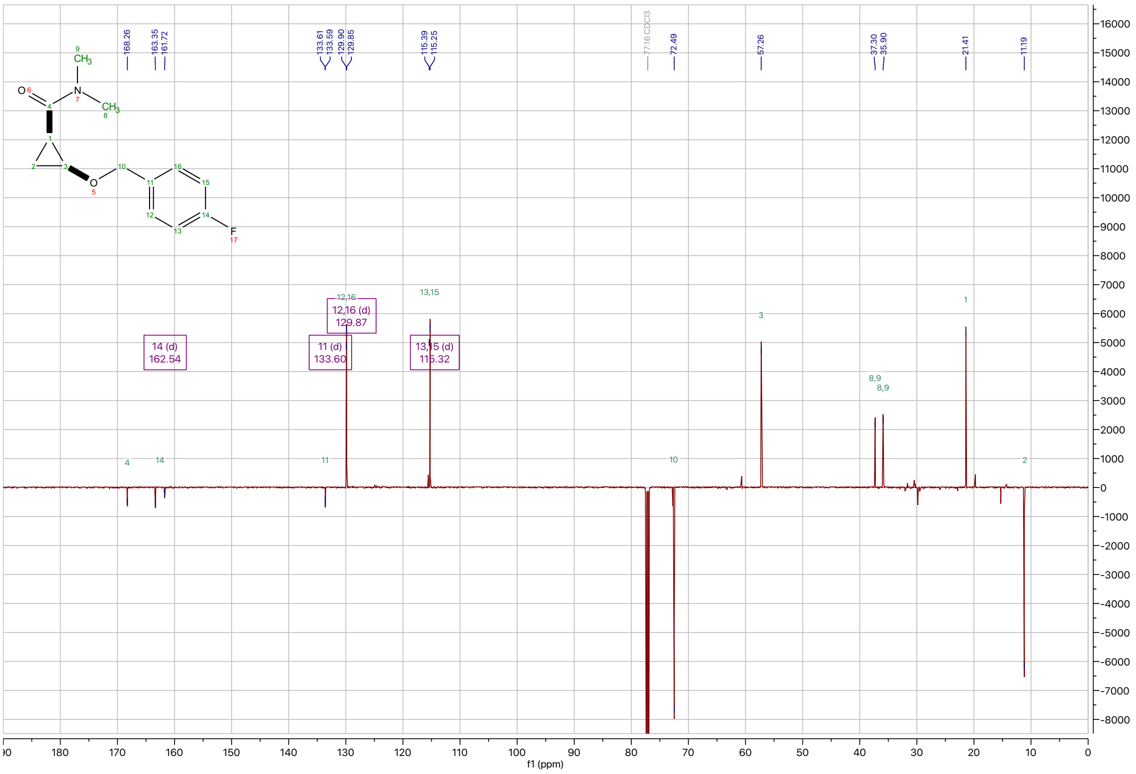

^{1}H-NMR spectrum of **7c**

^{1}H NMR (600 MHz, $CDCl_3$) δ 4.78 – 4.66 (m, 2H), 4.66 – 4.54 (m, 3H), 3.54 (ddd, *J* = 6.6, 6.6, 4.1 Hz, 1H), 3.16 (s, 3H), 2.99 (s, 3H), 1.81 (ddd, *J* = 9.0, 6.8, 6.8 Hz, 1H), 1.53 (ddd, *J* = 6.4, 6.3, 4.1 Hz, 1H), 0.88 (ddd, *J* = 8.9, 6.1, 6.1 Hz, 1H).

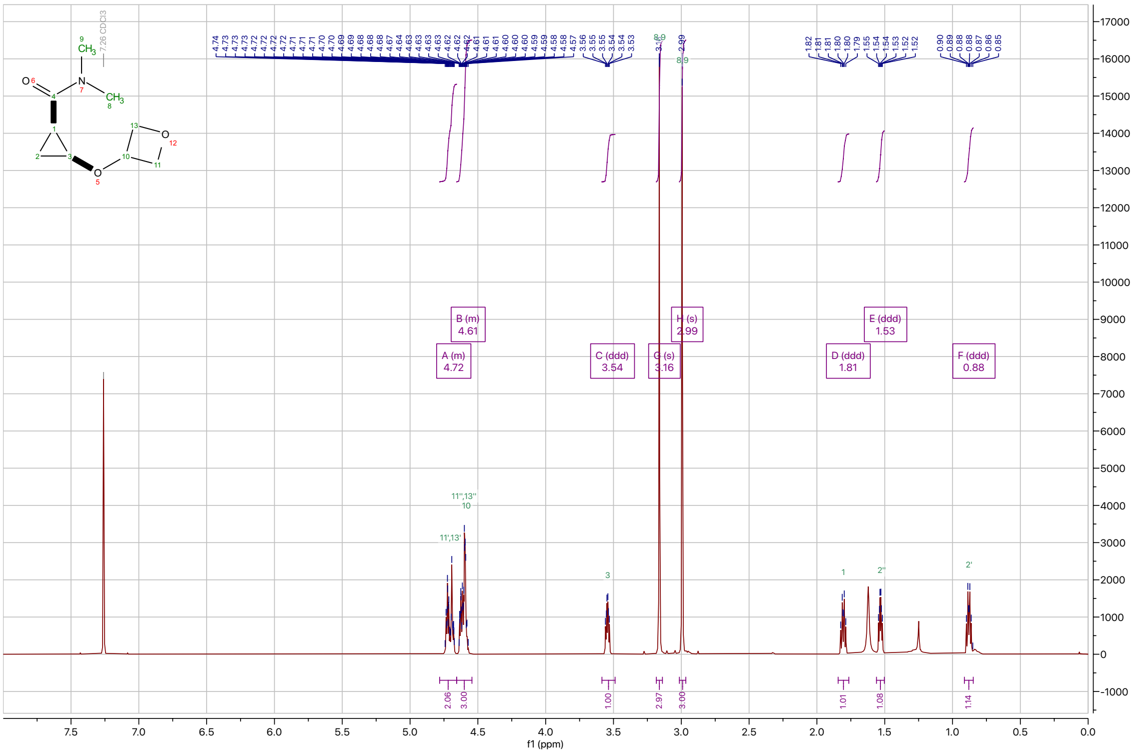

^{13}C-NMR spectrum of **7c**

^{13}C NMR (151 MHz, $CDCl_3$) δ 167.9, 78.7, 78.5, 72.7, 56.2, 37.2, 35.9, 21.1, 10.8.

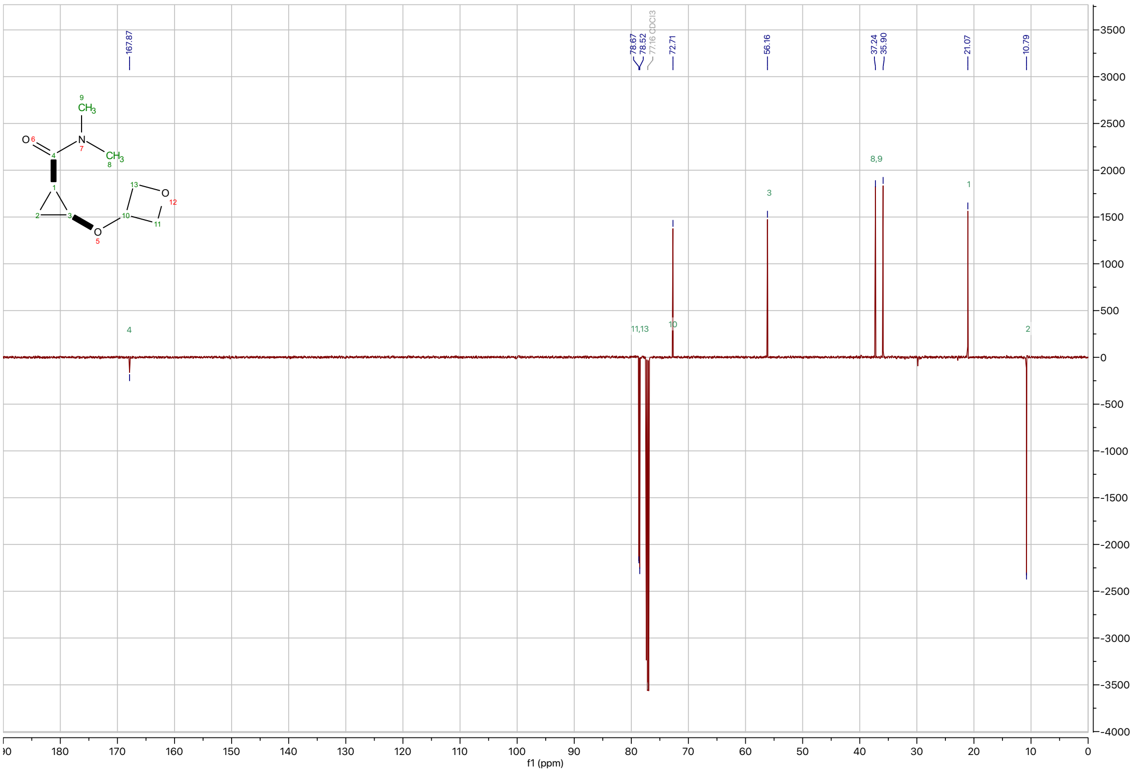

^{1}H-NMR spectrum of **7c'**

^{1}H NMR (500 MHz, $CDCl_3$) δ 4.81 – 4.74 (m, 2H), 4.70 – 4.58 (m, 3H), 3.55 (ddd, J = 6.3, 3.9, 2.1 Hz, 1H), 3.16 (s, 3H), 2.94 (s, 3H), 1.96 (ddd, J = 9.6, 5.9, 2.2 Hz, 1H), 1.28 – 1.21 (m, 1H), 1.14 (ddd, J = 9.4, 5.2, 3.9 Hz, 1H).

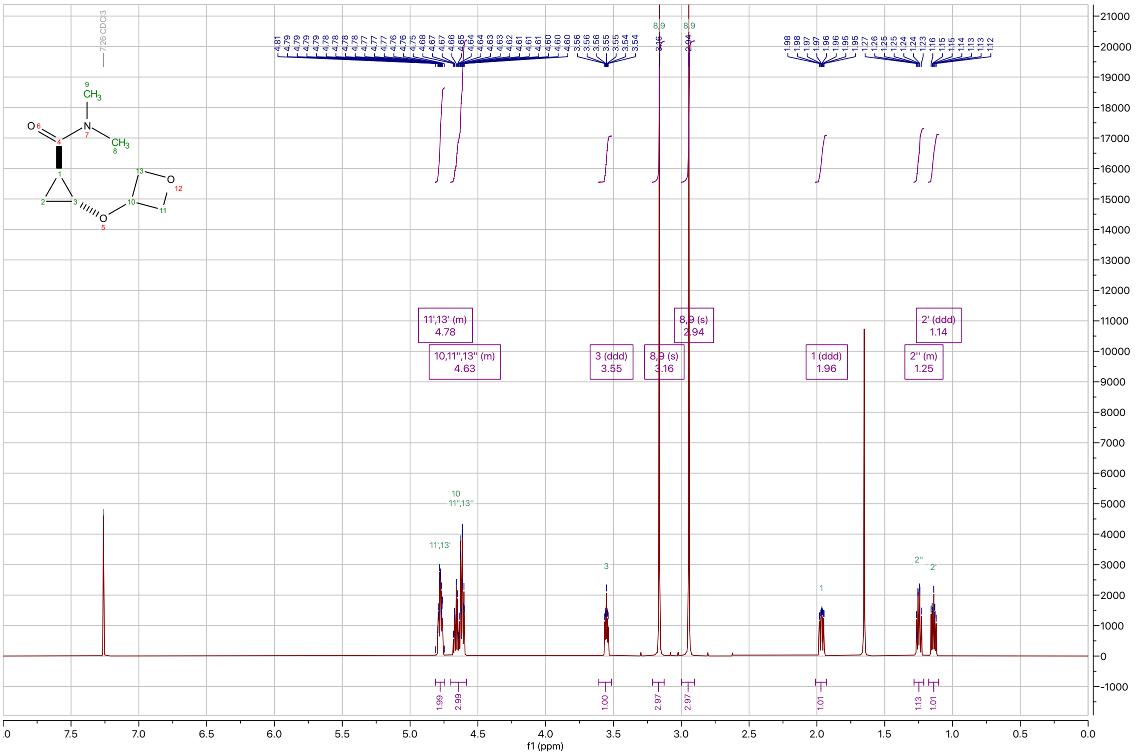

^{13}C-NMR spectrum of **7c'**

^{13}C NMR (126 MHz, $CDCl_3$) δ 171.1, 78.7, 78.5, 72.7, 59.0, 37.5, 35.8, 19.2, 15.1.

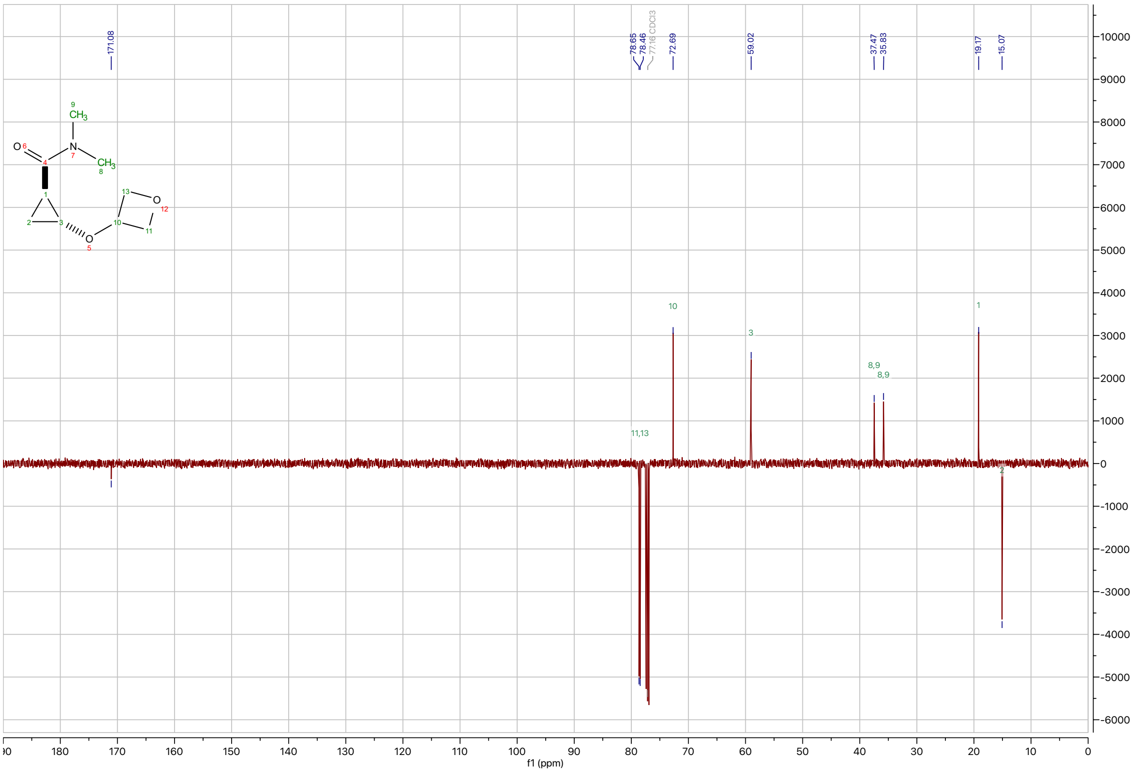

^{1}H-NMR spectrum of **7d**

^{1}H NMR (600 MHz, $CDCl_3$) δ 6.98 – 6.91 (m, 4H), 4.05 – 3.98 (m, 1H), 3.12 (s, 3H), 2.93 (s, 3H), 2.04 (ddd, J = 9.0, 6.9, 6.9 Hz, 1H), 1.73 – 1.67 (m, 1H), 1.17 (ddd, J = 9.0, 6.2, 6.2 Hz, 1H).

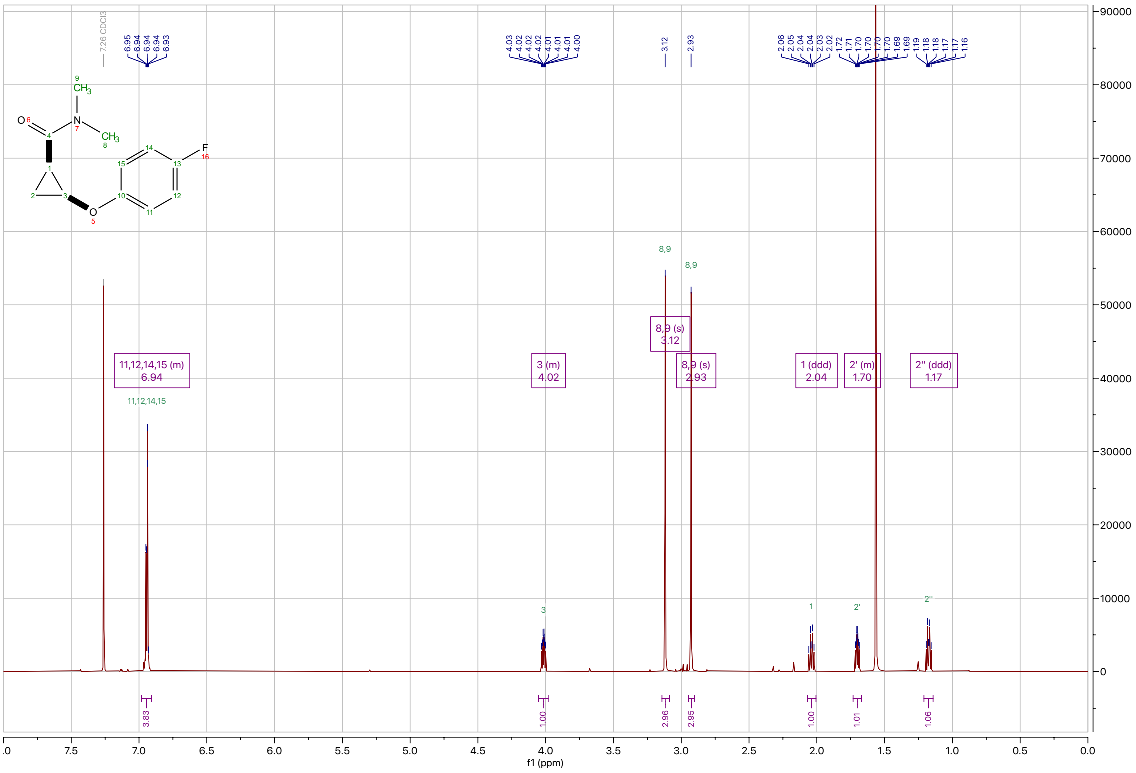

^{13}C-NMR spectrum of **7d**

^{13}C NMR (151 MHz, $CDCl_3$) δ 167.3, 157.9 (d, J = 238.9 Hz), 154.7 (d, J = 2.3 Hz), 116.7 (d, J = 8.2 Hz), 115.8 (d, J = 23.2 Hz), 55.5, 37.3, 35.9, 21.2, 11.7.

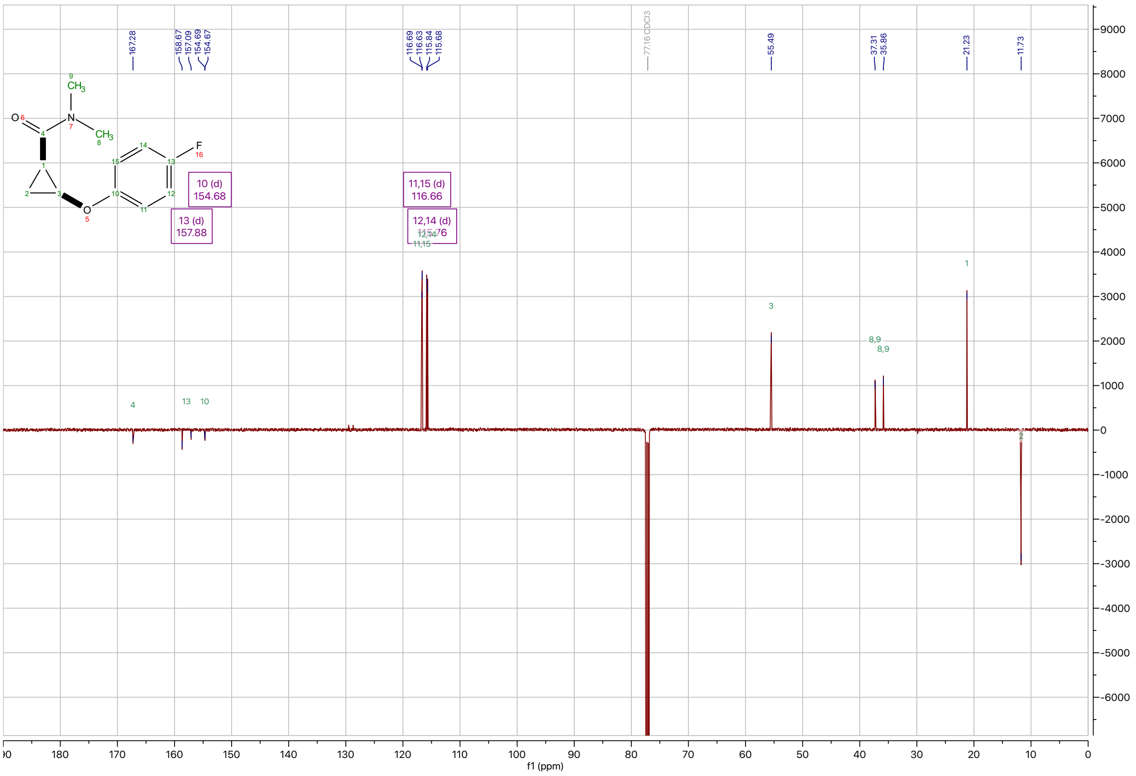

^{1}H-NMR spectrum of **7d'**

^{1}H NMR (600 MHz, $CDCl_3$) δ 7.01 – 6.94 (m, 2H), 6.94 – 6.88 (m, 2H), 4.07 (ddd, *J* = 6.3, 3.8, 2.2 Hz, 1H), 3.15 (s, 3H), 3.00 (s, 3H), 2.04 (ddd, *J* = 9.8, 6.1, 2.2 Hz, 1H), 1.50 (ddd, *J* = 6.3, 6.3, 5.5 Hz, 1H), 1.34 (ddd, *J* = 9.5, 5.5, 3.8 Hz, 1H).

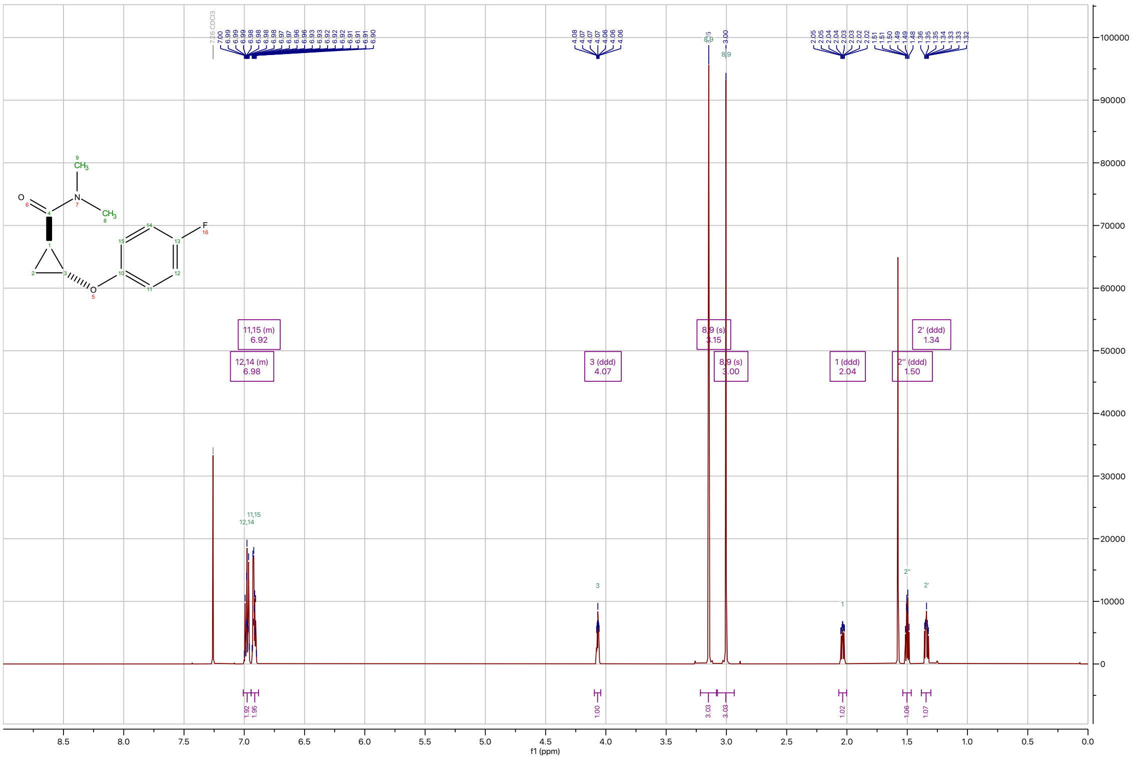

^{13}C-NMR spectrum of **7d'**

^{13}C NMR (151 MHz, $CDCl_3$) δ 170.7, 157.8 (d, *J* = 238.9 Hz), 154.5 (d, *J* = 2.1 Hz), 116.1 (d, *J* = 23.2 Hz), 115.9 (d, *J* = 8.1 Hz), 57.9, 37.5, 35.9, 20.0, 15.6.

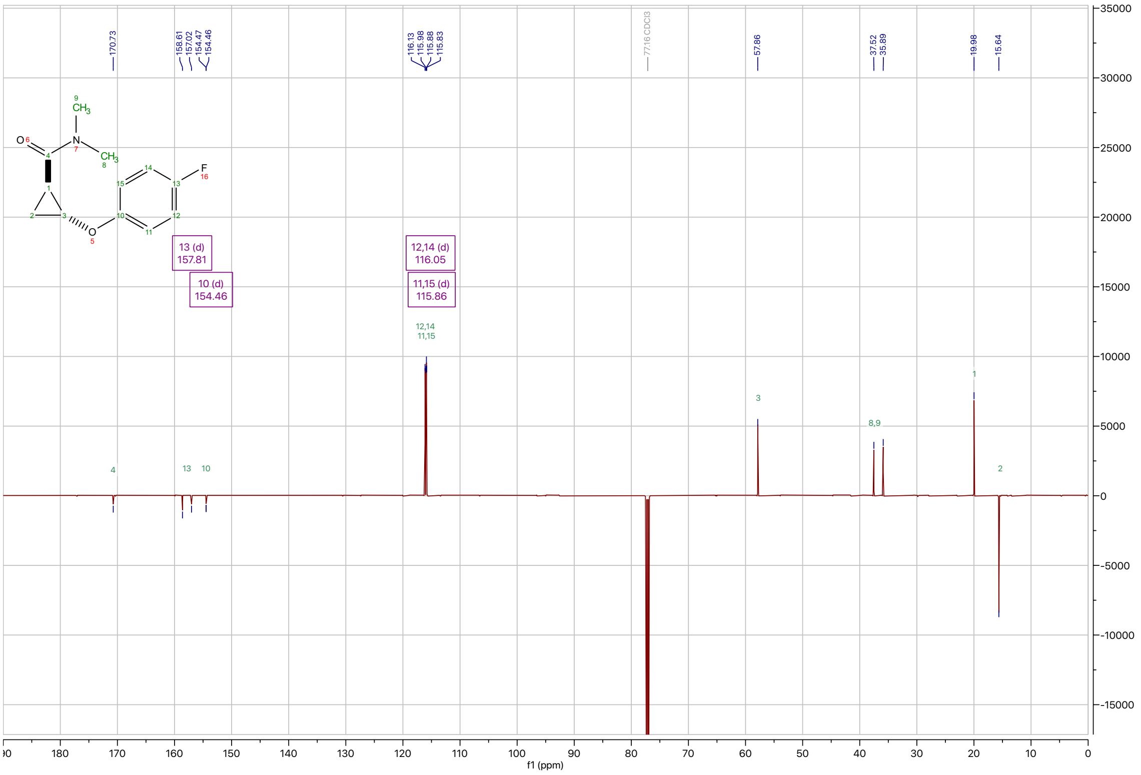

Supplement: Supplementary file 1 — ml2c00503_si_001.pdf [file ml2c00503_si_001.pdf]
